# The Toolbox for Fiber Flax Breeding: A Pipeline From Gene Expression to Fiber Quality

**DOI:** 10.3389/fgene.2020.589881

**Published:** 2020-11-12

**Authors:** Dmitry Galinousky, Natalia Mokshina, Tsimafei Padvitski, Marina Ageeva, Victor Bogdan, Alexander Kilchevsky, Tatyana Gorshkova

**Affiliations:** ^1^Laboratory of Plant Glycobiology, Kazan Institute of Biochemistry and Biophysics, FRC Kazan Scientific Center of RAS, Kazan, Russia; ^2^Laboratory of Ecological Genetics and Biotechnology, Institute of Genetics and Cytology, The National Academy of Sciences of Belarus, Minsk, Belarus; ^3^Cellular Network and Systems Biology Group, University of Cologne, CECAD, Cologne, Germany; ^4^Laboratory of Microscopy, Kazan Institute of Biochemistry and Biophysics, FRC Kazan Scientific Center of RAS, Kazan, Russia; ^5^Laboratory of Fiber Flax Breeding, Institute of Flax, Ustie, Belarus; ^6^Laboratory of Plant Cell Growth Mechanisms, Kazan Institute of Biochemistry and Biophysics, FRC Kazan Scientific Center of RAS, Kazan, Russia

**Keywords:** transcriptome-based selection, flax (*Linum* L.), gene expression, fiber quality, intrusive growth, tertiary cell wall, linear regression models

## Abstract

The goal of any plant breeding program is to improve quality of a target crop. Crop quality is a comprehensive feature largely determined by biological background. To improve the quality parameters of crops grown for the production of fiber, a functional approach was used to search for genes suitable for the effective manipulation of technical fiber quality. A key step was to identify genes with tissue and stage-specific pattern of expression in the developing fibers. In the current study, we investigated the relationship between gene expression evaluated in bast fibers of developing flax plants and the quality parameters of technical fibers measured after plant harvesting. Based on previously published transcriptomic data, two sets of genes that are upregulated in fibers during intrusive growth and tertiary cell wall deposition were selected. The expression level of the selected genes and fiber quality parameters were measured in fiber flax, linseed (oil flax) cultivars, and wild species that differ in type of yield and fiber quality parameters. Based on gene expression data, linear regression models for technical stem length, fiber tensile strength, and fiber flexibility were constructed, resulting in the identification of genes that have high potential for manipulating fiber quality. Chromosomal localization and single nucleotide polymorphism distribution in the selected genes were characterized for the efficacy of their use in conventional breeding and genome editing programs. Transcriptome-based selection is a highly targeted functional approach that could be used during the development of new cultivars of various crops.

## Introduction

For thousands of years mankind has made effective use of fiber crops for various purposes. Currently, fiber crops produce the raw materials for the textile industry, and also for eco-friendly building materials, cosmetics, car manufacturing, and medicine, etc. Interest in natural fibers is also growing due to environmental issues and new legislation that is driving the development of a market for biodegradable and recyclable materials (Smole et al., 2013).

There are numerous plant sources of natural fibers, which are often classified according to their plant tissue of origin. Accordingly, there are three main categories: (1) bast fibers of the stem (flax, hemp, jute, ramie, kenaf, etc.), (2) leaf fibers (sisal, banana, manila hemp, pineapple, etc.), and (3) seed fibers (coir, oil palm, etc.) (Jawaid and Khalil, 2011). More than eight million households worldwide are involved in the production of plant-based fibers from the above listed groups (excluding cotton). With a global production of around six million metric tons, the market share of these plant-based fibers was around 5.7% of total global fiber production volume in 2018 (based on FAOStat and Total Global Production Volumes compiled by Textile Exchange^1^). The global flax fiber and tow production in 2018 is estimated at around 780 metric tons, with 93% of flax production coming from Europe (data for the last decade^2^). Flax is grown for its cellulose-rich fibers and seeds, which can be ground into a meal or turned into linseed oil. The long bast fibers are traditionally used in the textile industry for the production of linen or mixed fiber textiles, and, together with shorter xylem fibers they are also used in the automobile and construction industries (Baley et al., 2006; Kymäläinen and Sjöberga, 2008).

Flax bast fibers are primary fibers that originate from the procambium (Esau, 1943) and are similar to a number of primary bast fibers of other plant species (hemp, ramie, and nettle) that form a thick cellulose-rich cell wall that we have named the tertiary cell wall (TCW) (Gorshkova et al., 2018a). Before cell wall thickening is initiated, the fibers undergo a specific type of cell elongation, called intrusive growth, that occurs in the depths of other stem tissues (Ageeva et al., 2005; Snegireva et al., 2010). This stage of development has a notable impact on the yield and quality of fibers, and it has been largely underestimated and poorly characterized from a molecular-genetic point of view (Snegireva et al., 2015; Mokshina et al., 2018). Structure and composition of the bast fiber TCW is similar to the G-fiber cell wall in tension wood (Gorshkova et al., 2015, 2018a), and is quite distinct from the other cell wall types. The high cellulose content (up to 90%), the presence of rhamnogalacturonan I, and absence of xylan and lignin are specific distinguishing features of the TCW. The polymer arrangement also differentiates TCW from primary and secondary cell walls; cellulose microfibrils in the TCW lie close to the axial orientation and are poorly interlaid by matrix polymers (Gorshkova et al., 2018a). Both the chemical composition and spatial organization of polymers in the cell wall supramolecular structure contribute to the natural strength, flexibility, smoothness, and linearity of flax fibers.

Fiber crop producers aim to achieve excellent fiber quality; this is a complex feature that is characterized by a number of physical parameters measured after plant harvest and partial processing. These parameters include tensile strength, density, flexibility, and color grade (Akin, 2005) and they are widely used for the evaluation of technical fibers (here and after, the term “technical fiber” is used for fiber bundles obtained from harvested plants, while the term “fiber” is used in its biological sense, meaning individual plant cell with specific characteristics).

Quality depends both on the peculiarities of fiber development *in planta* and the way in which the stems are processed after crop harvest. The importance of the biological basis of fiber quality is reflected in pronounced differences in technical fiber parameters between various crop cultivars. There are numerous studies describing the anatomical, morphological and biochemical differences between various cultivars that are related to fiber quality (Elhaak et al., 1999; Couture et al., 2002, 2004; Rennebaum et al., 2002). However, to manipulate these quality parameters it is necessary to understand their, as yet, elusive genetic basis. The comprehensive approaches of genomics and transcriptomics may help to fill this gap.

Flax is a bast fiber crop on which the most advanced molecular studies of fiber development have been conducted. The whole genome sequence of cultivated flax was assembled in 2012 (Wang et al., 2012), and was used for SNP (single nucleotide polymorphism) discovery (Kumar et al., 2012), transcriptomic profiling of different tissues (Dmitriev et al., 2016, 2017; Zhang and Deyholos, 2016) and construction of a high resolution consensus map of flax that integrated genetic and physical data (Cloutier et al., 2012). These approaches considerably advanced the earlier important studies to large-scale molecular characterization of the flax genome and transcriptome (Day et al., 2005; Roach and Deyholos, 2007; Fenart et al., 2010). Further RNA-Seq experiments revealed those genes that are highly upregulated during intrusive growth (Gorshkova et al., 2018b) and TCW deposition (Gorshkov et al., 2017), and described genes with fiber-specific expression that are barely transcribed in other tissues (Mokshina et al., 2020). These studies opened the possibility of relating the structure and expression of genes important for fiber development to fiber quality.

In the current study, we concentrated on the establishment of a pipeline that could be used to select a few genes that have high potential for manipulating fiber quality. Our approach included five key steps: (1) selection of candidate genes using previously published RNA-Seq data (Gorshkov et al., 2017, 2019; Mokshina et al., 2020); (2) evaluation of their expression in various developing flax genotypes; (3) determination of technical fiber quality parameters in these flax genotypes after plant harvest; (4) identification of the optimal sets of genes whose expression is closely related to fiber quality features by regularized regression models; and (5) analysis of SNP abundance across the coding regions of genes important for bast fiber development using RNA-Seq data obtained for several different flax genotypes. We suggest that the obtained results may help to advance conventional breeding and genome editing programs for flax and other fiber crops.

## Materials and Methods

### Plant Material and Experimental Design

Ten different flax genotypes were used as the plant objects: six cultivars of fiber flax (Aramis, Eden, Grant, Laska, Drakkar, and Mogilevsky), two cultivars of linseed flax (Lirina and Orpheus), and two wild flax species [*L. usitatissimum* ssp. *bienne* (Mill.) Stank., and *L. angustifolium* Huds.]. Cultivars of fiber flax and linseed flax belong to *L. usitatissimum*. Altogether, the analyzed genotypes were designated as the FLW group [from fiber (F), linseed (L), and wild (W) flax]. Flax plants were grown on an experimental field of the Institute of Flax of the National Academy of Sciences of Belarus (Ustie, Vitebsk region, Belarus; 54°26′02″ S, 30°21′05″ W in 2018 and 54°26′03″ S, 30°20′50″ W in 2019). The experimental plots were organized according to the type of plant collection nursery, based on guidelines for studying the collection of flax (*Linum usitatissimum* L.) (No author listed, 1988). Plant material for each genotype was collected in 2018 and 2019.

**FIGURE 1:**
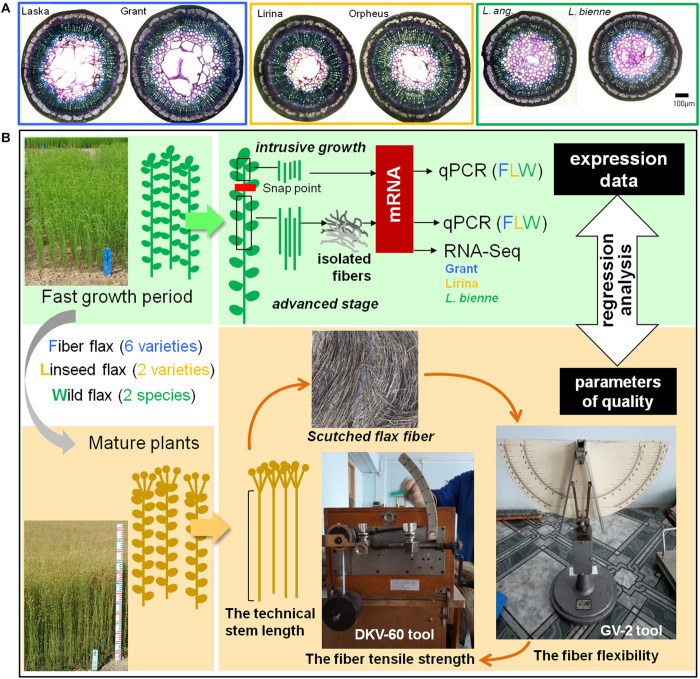
Cross-sections of the representatives of fiber, linseed flax cultivars, and wild flax species **(A)**. Scheme of sample collection for further analysis of gene expression and fiber quality **(B)**.

Samples for gene expression analysis were collected during the fast growth period of plant development (around 45 days after sowing) when both intrusive growth and TCW deposition are well pronounced. The stem segments located above the snap point, a marker of fiber developmental transition from elongation to cell wall thickening (Gorshkova et al., 2003), were used to evaluate the expression of genes whose products were involved in intrusive growth (Figure 1). For this, a 1 cm portion was removed from the top of the stem and the underlying 2 cm stem portion was collected. All leaves were removed from the collected stem fragments.

Gene expression at the advanced stage of fiber specialization was determined in fibers isolated from the stem part below the snap point. To isolate fibers at the stage of TCW deposition, a 10 cm-long stem portion, starting from 1 cm below the snap point was cut downward the stem (Figure 1). The fiber-enriched part was peeled off and the peel was washed in 80% ethanol to isolate bast fibers (Mokshina et al., 2014).

The collected stem parts used to characterize gene expression at fiber intrusive growth and isolated fibers to study TCW deposition were frozen in liquid nitrogen and used for RNA extraction. Three biological replicates at each of the analyzed developmental stages were used for RNA extraction. Each biological replicate included samples from five individual plants. Samples for the determination of technical fiber quality were obtained from mature plants (Figure 1).

### RNA Extraction and RT-qPCR

RNA was extracted using “ExtractRNA” (Evrogen, Moscow, Russia) and afterward purified using an RNeasy Mini Kit (Qiagen, Hilden, Germany). Obtained RNA samples were treated using a Turbo DNase Kit (Ambion, Austin, TX, United States). RNA concentration and integrity were tested with agarose gel electrophoresis and a NanodropND-1000 spectrophotometer (NanoDrop Technologies, Wilmington, DE, United States). Reverse transcription was performed using RevertAid H Minus Reverse Transcriptase (Thermo Fisher Scientific, Waltham, MA, United States) and Oligo (dT) 12-18 Primer (Invitrogen, Carlsbad, CA, United States) according to the manufacturer’s recommendations. Every cDNA synthesis reaction was accompanied by NRT (no reverse transcriptase control). The obtained cDNA and NRT samples were diluted 10 times and used as templates for qPCR. qPCR was performed using a 2.5× Reaction Mix for qPCR with EVA Green dye (Syntol, Moscow, Russia) and accompanied by NRT and NTC (no template control). Sequences for gene-specific primers for the analyzed genes are presented in Supplementary Table S1. *LusGAPDH*, *LusETIF1*, and *LusETIF5A* genes were used as reference genes for qPCR data normalization (Huis et al., 2010). The stability of reference gene expression was tested by the geNorm algorithm implemented in CFX Maestro Software (Bio-Rad, Hercules, CA, United States) (Vandesompele et al., 2002). We computed the ΔCq value for evaluation of gene expression and gene clustering. The ΔCq values were calculated as the difference between the geometric mean of three reference gene Cq values and the Cq values of the genes of interest. The off-scale Cq values were assigned the highest Cq value measured for this gene in other samples plus 2 (Bergkvist et al., 2010). Two technical repetitions were performed for each of three biological replicates. All obtained ΔCq values can be found in Supplementary Table S2.

### Flax Samples Used for Comparison of Transcriptome Profiles to Select Candidate Genes

Bioinformatic analysis of previously published RNA-Seq data (Mokshina et al., 2020) was performed after normalization applied to all samples simultaneously to ensure that the expression values were comparable across all samples. The procedure of normalization was described previously (Mokshina et al., 2020). Briefly, Illumina raw reads (as FASTQ files) of each sample were filtered using the BBDuk utility of BBToolsv 37.02^3^. The clean reads were mapped onto the flax genome sequence scaffolds using HISAT2 v2.1.083 (Kim et al., 2015). The aligned reads were aggregated with StringTie v2.0 (Pertea et al., 2016) using the annotation of flax genes as Lusitatissimum_200_v1.0. gff3^4^ (Wang et al., 2012) to provide total gene read (TGR) counts for each gene. The R package DESeq2 v.1.14.1 (Love et al., 2014) was used to perform the differential expression analysis of mRNA from all samples using TGR counts. The DESeq estimateSizeFactors and estimateDispersions functions (with the default options) were used to obtain normalization factors for each sample and to normalize the TGR counts. Most of the transcriptomic data were obtained for the fiber flax cultivar Mogilevsky, with the exception of SAM (shoot apical meristem), LEAF, and ROOT samples (Table 1).

**TABLE 1:** The sources of used transcriptomic data.

Sample name	Description	References	Project accession
SAM	Shoot apical meristem, 0.5 mm	Zhang and Deyholos (2016)	PRJNA229810
cPAR	Cortical parenchyma, 0.3–2.5 cm from the stem apex, the same part of stem as iFIB	Gorshkov et al. (2019)	PRJNA475325
iFIB	Intrusively growing fibers with PCW, 0.3–2.5 cm from the apical part (microdissected), average for iFIBa and iFIBb samples	Gorshkova et al. (2018b, 2019)	PRJNA475325
tFIBa	Fibers depositing tertiary cell wall, 5 cm long stem portion starting 1 cm below snap point, earlier stage	Gorshkov et al. (2019)	PRJNA475325
tFIBb	Fibers depositing tertiary cell wall, 5 cm long stem portion, at the 3 cm above cotyledon leaves, later stage	Mokshina et al. (2020)	PRJNA631357
sXYLa	Xylem part enriched in secondary cell walls (the same segment of the stem as for tFIBa)	Gorshkov et al. (2019)	PRJNA475325
sXYLb	Xylem part enriched in secondary cell walls, collected from the same stem segment as tFIBb	Mokshina et al. (2020)	PRJNA631357
LEAF	Upper leaves from individual plants under normal conditions	Dmitriev et al. (2016)	PRJNA340243
ROOT	Root tips (5 mm) of control plants	Dmitriev et al. (2017)	PRJNA412801

For the selection of genes upregulated at fiber intrusive growth, genes that had a four-fold increased level of expression in intrusively growing fibers (iFIB) compared to other tissues, and a total gene reads (TGR) ≥ 50 in iFIB were considered. Out of 28 genes that fitted these criteria, 17 genes were selected (close paralogs were excluded).

For the selection of genes upregulated at TCW deposition, genes that had four-fold increased levels of expression in fibers forming TCW (tFIBa) compared to other tissues, and a TGR ≥ 250 in tFIBa were considered. Out of 38 genes that fitted these criteria, 27 were selected. Additionally, five genes related to cellulose biosynthesis (*LusCESA1, LusCESA3-B, LusCESA4*, and *LusCESA8-A*) and chitinase-like gene (*LusCTL2*) were included into the list.

### SNP Search in Coding Regions of the Selected Genes After RNA-Seq Analysis of Several Genotypes

RNA-Seq analysis was performed for fibers at the TCW-deposition stage of development isolated from three flax genotypes: fiber flax (Grant), linseed flax (Lirina), and a wild species (*L. bienne*) harvested in 2019. Total RNA was extracted and purified from the same samples as used for qPCR. The RNA integrity test using an Agilent 2100 bioanalyzer and pair-end RNA sequencing using an Illumina HiSeq 6000 were performed at Novogene in Europe^5^. Raw data were analyzed using Illumina CASAVA 1.8, and three types of sequence reads, namely, reads with adapter, no exact base information, or low-quality reads, were filtered and removed. The genome of a medium-late-maturing oilseed flax (*Linum usitatissimum* L.) CDC Bethune (Rowland et al., 2002) was used as reference genome^4^. The processed data with sequence reads and alignments were generated based on HISAT2 software. Mapping results were provided in BAM format by Novogene. BAM-files were used for SNP calling. Variant calling (SNP and InDel searching) was performed for scaffolds from the reference genome that contained the selected 32 genes, with all the BAM-files simultaneously. We used VarScan v2.3.9 with default settings except for “Minimum read depth at a position to make a call,” which was increased to 20. Data for the analysis were pre-generated by the Samtools v1.5 mpileup command^6^. The obtained variants were annotated using RegTools to differentiate variants located in genes’ exons from others. We included in the final table only those SNPs that were identified in all three biological replicates for each sample. All identified SNPs (at least in one biological replicate) can be found in Supplementary Table S3.

We anchored the selected genes into the physical map of flax chromosomes in order to assess their potential linkage with mapped markers (Supplementary Table S4). We used chromosome-scale pseudomolecules (You et al., 2018), genetic maps (Cloutier et al., 2012), as well as gene annotation of the phytozome.gov portal^7^.

RNA-Seq data for 32 genes (TCW-upregulated) were used for validation of qPCR data (Supplementary Figure S1).

### Technical Fiber Quality Evaluation

Flax straw was subjected to dew-retting and processed on a scutching-and-breaking machine SMT-200M according to the quality standards in Belarus (STB1195, 2008; Dyagilev et al., 2016). We evaluated the technical length of the plant stem, tensile strength, and flexibility of scutched fiber. At least three independent measurements were performed for each quality feature in each biological replicate.

The technical stem length of flax is defined as the length of the stem without side branches (Diederichsen and Fu, 2006). It was measured in cm as a length from the root collar to the point where the stem splits into floral side branches.

The fiber flexibility of the scutched flax fiber was measured using the GV-2 tool (Figure 1). We took a 27-cm-long fiber bundle weighing 420 mg and placed it on the horizontal surface of the GV-2 tool. Then supporters on the horizontal surface of the tool were released and fiber sag (in mm) under natural gravity was determined. The value was measured for both sides of the sample and the mean was calculated.

The tensile strength of the scutched flax fiber was evaluated using a DKV-60 tool (modification of the stelometer that was adapted for flax fiber; Figure 1). The same sample that was used for flexibility measurement was used here. The fiber sample was fixed between clamps on the device; the distance between the clamps was 100 mm. The force applied to the clamps was then increasing until the fiber broke. The force (in Newtons) was recorded at the time of fiber rupture. The cross-sectional area of the analyzed technical fiber sample was about 1 mm^2^. Therefore, the force of tensile strength measured in Newtons was numerically equal to the tensile strength in MPa.

### Statistical Analyses

The obtained data were analyzed in R (R Core Team, 2013); *ggplot2* and *ggpubr* packages were used for data visualization, *stargazer* package was used to report regression output.

The arithmetic means were calculated for independent values of ΔCq and fiber quality features in biological replicates, and then used for computing the gene-phenotype correlation. The data obtained for technical fiber quality and relative expression were tested for normal distribution using the Shapiro–Wilk test. For data that were normally distributed, we used Pearson’s correlation coefficient (PC) and ANOVA test, and the Spearman’s correlation coefficient (SC) and the Kruskal–Wallis test, if not. When necessary, the results of statistical tests were adjusted for the false discovery rate to account for multiple testing.

Co-expression heatmaps were generated with the heatmaply v1.1.0 R package (Galili et al., 2018) using ΔCq of gene expression and Spearman’s correlation coefficient, respectively. Genes and samples (flax cultivars) were clustered using Euclidean distances and the complete-linkage hierarchical clustering method. Gene co-expression heatmaps were generated with corrplot v0.84 R package (Wei and Simko, 2017) using Pearson correlation of ΔCq expression values.

A linear regression was used to model each fiber quality parameter (dependent variable) with ΔCq of the studied gene(s) [independent variable(s)]. Simple and multiple linear regression models were constructed, and the coefficient of determination (*R*^2^), the adjusted *R*^2^, and statistical significance of the beta coefficients were used to assess the quality of obtained models. To select a few predictive features for each of the mechanical parameters we used a form of regularized regression called LASSO (Tibshirani, 1996) with stability selection (Meinshausen and Buhlmann, 2010). This machine learning approach attempts to optimize the variance-bias trade-off in a high dimensional setting, i.e., when the number of independent variables is greater than the number of observations. Alternatively, the stepwise forward selection regularization (Efroymson, 1960) was used to select larger models with better predictive performance for the price of higher bias.

## Results

### Selection of Genes to Relate Their Expression to Quality Parameters

Genes whose expression had the potential to influence fiber quality were selected on the basis of data from previously published RNA-Seq experiments. We used transcriptomes of various flax tissues (Table 1) to identify the genes important for two major stages of bast fiber development: intrusive growth and TCW deposition.

Genes whose expression was important during fiber intrusive growth were selected among the specifically and significantly upregulated genes (>4-fold) at this stage of fiber development (sample iFIB), as compared to all other analyzed samples from other plant tissues and other stages of fiber development (Table 2A). The 17 selected genes (designated as Intrusive-upregulated) encoded products with different functional specialization: transcription factors, enzymes for polysaccharide modification, hormonal response proteins, and one protein with unknown function. For example, genes for putative transcription factor *LusBZIP5* (orthologous to *AT3G49760*) and transcriptional activator *LusASML2* [orthologous to *AT3G12890* encoding activator of spomin::LUC2 that regulates the expression of at least a subset of sugar-inducible genes (Masaki et al., 2005)], and auxin-responsive GH3 family protein *Lus10003598* (orthologous to *AT5G54510* and *DFL1*) were among the selected genes. Also, a number of O-Glycosyl hydrolases belonging to family 17 (four genes) that can be involved in callose metabolism were selected, because of their specific activation during intrusive growth (Gorshkova et al., 2018b).

**TABLE 2:**
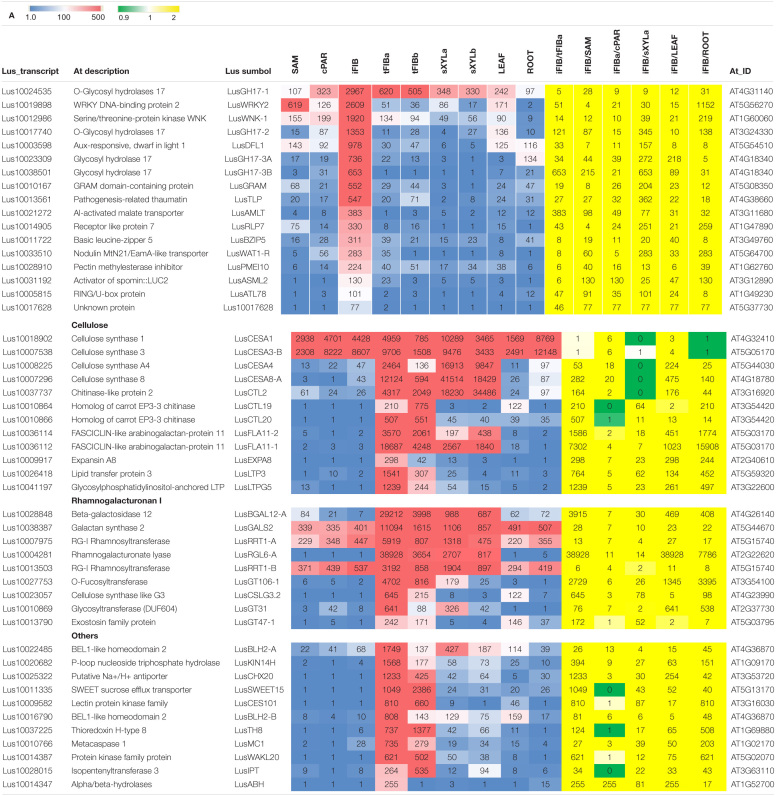
RNA-Seq data (TGR) for the selected genes upregulated in fibers in the course of intrusive growth **(A)** and during tertiary cell wall formation **(B)** in different flax tissues.

*SAM, shoot apical meristem (Zhang and Deyholos, 2016); cPAR, cortical parenchyma; iFIB, intrusively growing fibers (Gorshkova et al., 2018b); tFIBa, fibers depositing tertiary cell wall, earlier stage (Gorshkov et al., 2019); tFIBb, fibers depositing tertiary cell wall, later stage (Gorshkov et al., 2017); sXYLa, xylem part, the same segment of the stem as for tFIBa (Gorshkov et al., 2019); sXYLb, xylem part, the same segment of the stem as for tFIBb (Mokshina et al., 2020); LEAF, upper leaves from individual plants under normal conditions (Dmitriev et al., 2016); ROOT, root tips, average for control samples (Dmitriev et al., 2017). When calculating the FC, to avoid division by zero, +1 was added to all the TGR values. The blue-red color scale reflects the level of expression, the green-yellow one – the ratio between the designated samples.*

Among 32 genes encoding the products that could play a significant role during TCW formation, 27 genes designated as TCW-upregulated had higher expression levels in fibers at this stage of development as compared to intrusively growing fibers (tFIBa/iFIB) and the apical part of the stem (tFIBa/SAM, Table 2B). Furthermore, they were significantly (>4-fold) upregulated in fibers forming TCW as compared to other samples such as xylem, leaves, and roots (Table 2B). Since the TCW is known as a cellulose-rich structure, we added five genes related to cellulose biosynthesis: four cellulose synthase genes (*LusCESA1, LusCESA3-B, LusCESA4*, and *LusCESA8-A*) and the chitinase-like gene (*LusCTL2*). In ramie, the *CESA3* and *CESA8* were among the cell wall related DEGs revealed in comparative transcriptomics of stem bark samples collected from three regions associated with bast fiber development) (Xie et al., 2020); in hemp hypocotyl, the orthologs of *A. thaliana CESA4, CESA7* and *CESA8* were among the SCW-related genes upregulated at later stages of development (Behr et al., 2016). Along with cellulose, an important role in TCW formation and functioning is played by pectic RG-I, despite its relatively low content of around 7% (Gorshkova et al., 2018a). For this reason, we chose a number of genes associated with RG-I biosynthesis and modifications, such as *LusRRT1*, *LusGT106-1* (biosynthesis of backbone; shown for *Arabidopsis*, Takenaka et al., 2018), *LusGALS* (biosynthesis of galactan side chains; shown for *Arabidopsis*, Liwanag et al., 2012), *LusBGAL12* (modification of galactan side chains; Roach et al., 2011), and *LusRGL6-A* (putative modification of RG-I backbone; Mokshina et al., 2019). The list of genes for analysis also included three glycosyltransferases belonging to 47 and 31 families (*LusGT47-1* and *LusGT31*) and the gene for cellulose synthase-like protein (*LusCSLG3*). The functions of these genes, even when putative, remain unknown, but they could also refer to RG-I biosynthesis (Gorshkov et al., 2017; Galinousky et al., 2020) and were specifically upregulated in fibers at the TCW-deposition stage (Table 2B). Most of the selected genes had a much higher mRNA abundance at an earlier stage of TCW deposition as evidenced by the comparison of TGR values for tFIBa and tFIBb.

Thus, two gene sets containing 17 and 32 genes important for intrusive growth and TCW formation, respectively, were used for further analysis with qPCR.

### Expression of Selected Genes in Samples From Various Flax Genotypes

Expression of the selected genes was evaluated in samples of 10 flax genotypes belonging to three groups of flax that have different applications and technical fiber quality: six cultivars of fiber flax (Aramis, Eden, Grant, Laska, Drakkar, and Mogilevsky), two cultivars of linseed flax (Lirina and Orpheus), and two wild flax species (*L. bienne* and *L. angustifolium*). We named the three listed groups as FLW group [from fiber (F) flax, linseed (L) flax, and wild (W) flax] though intraspecific and interspecific taxonomy of the *Linum* genus is difficult and is presented differently by various authors. For instance, *L. bienne* and *L. angustifolium* are considered synonymous in several databases (The Plant List, Flora Europaea, GRIN taxonomy, and NCBI-Taxonomy). Genome analysis with chromosome and molecular markers show that *L. bienne* must be considered as a subspecies of *L. usitatissimum* rather than a separate species (Muravenko et al., 2003), while morphologically *L. angustifolium* and *L. bienne* are closely related (Kutuzova et al., 2015). Based on breeding experiments, *L. angustifolium* was suggested to be the ancestor of Colchian flax, from which the linseed, intermediate, and finally fiber forms consequently evolved; the ancestor of *L. usitatissimum* ssp. *bienne* (hereafter, *L. bienne*) could be *L. angustifolium* or Colchian flax (Kutuzova et al., 2015).

The cross-sections of the various representatives of FLW group are exemplified in Figure 1. The number of fibers on the cross-sections of FLW representatives ranged from 444 ± 90 in Orpheus to 732 ± 80 in Drakkar, being lowest in linseed cultivars, intermediate in wild species, and the highest in fiber flax (data not shown). The average length of individual fibers obtained from mature plants ranged from 14.7 ± 2.0 mm in *L. angustifolium* to 21.5 ± 1.2 mm in Drakkar. The proportion of fibers longer than 30 mm constituted over 20% in fiber flax, while in L and W genotypes it was 8% and 12%, respectively. The proportion of short fibers (below 15 mm) was especially high in *L. angustifolium* (Supplementary Figure S2).

Transcript abundance of the selected genes was measured by RT-qPCR using the reverse transcription method in stem portions containing fibers at the intrusive growth stage and in isolated fibers at the advanced stage of fiber specialization. We did not isolate fiber cells at the intrusive growth stage and extracted RNA from whole stem parts because previously we have analyzed the transcriptome of fibers isolated by laser microdissection at this stage of development and revealed genes especially abounded in fiber cell at the intrusive growth stage as compared to other tissues (Gorshkova et al., 2018b); only such genes were analyzed in the current study to characterize expression at fiber elongation (Supplementary Table S1).

Most of the selected genes had increased expression in the fiber flax cultivars compared with linseed cultivars (*LusBGAL12-A, LusRGL6-A, LusRRTs, LusEXPA8A, LusGT31, LusFLA11s, LusGT47-1, LusLTP3, LusLTPG5, LusGT106-1, LusGALS2, LusBLHs*, and *LusSWEET15*), while in wild species gene expression was, as a rule, higher than in linseed ones, but lower than in fiber flax. The expression of some genes did not differ significantly in the FLW group (*LusCESA1, LusCESA3-B, LusCTL2, LusCTL20, LusMC1*, and *LusWAKL20*) (Supplementary Figures S3, S4).

**FIGURE 2:**
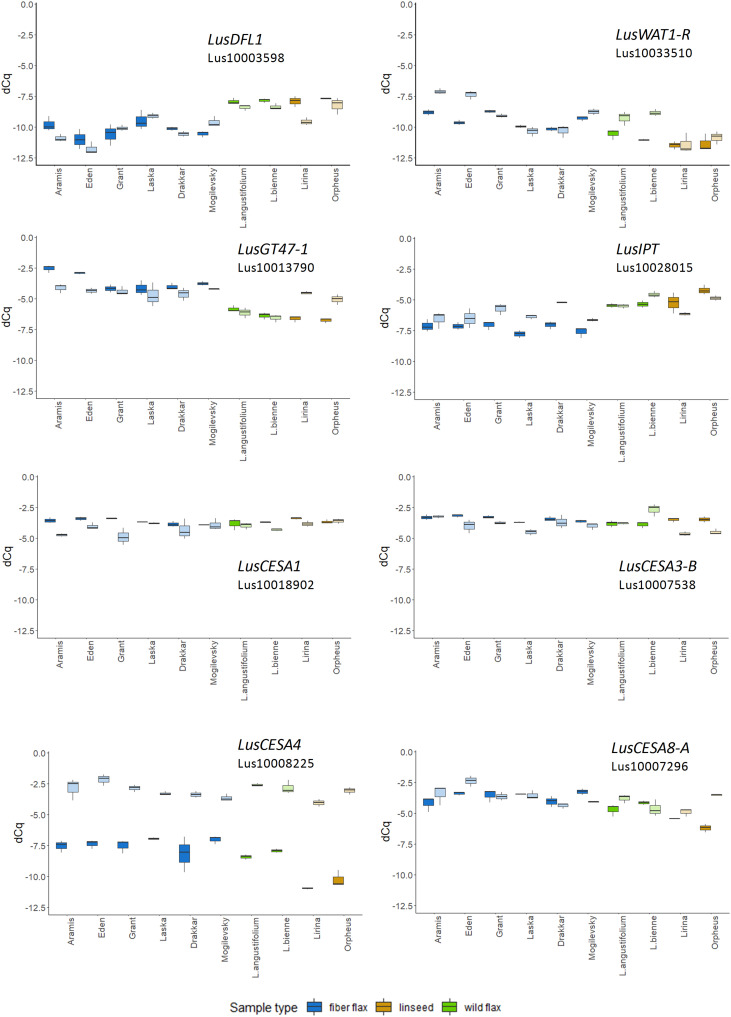
The relative level of expression (ΔCq) of *LusDFL1*, *LusWAT1-R*, *LusGT-47, LusIPT, LusCESA1, LusCESA3-B, LusCESA4*, and *LusCESA8-A* in isolated fibers of 10 flax cultivars (species) analyzed in 2018 and 2019. *LusGAPDH*, *LusETIF1*, and *LusETIF5A* genes were used as reference genes for qPCR data normalization. Fiber flax cultivars are marked in blue, linseeds in yellow, and wild species in green. Bright colors indicate samples from 2018, pale colors are those for 2019. The bottom and top of the box are the first and third quartiles, respectively; the bold line within the box is the median. The upper whisker extends from the third quartile to the largest value, but no further than 1.5 * IQR, where IQR is the interquartile range. The lower whisker extends from the first quartile to the smallest value at most 1.5 * IQR. “Outlying” points are plotted individually.

The expression of *LusGH17-1* and *LusWNK-1* genes, as with *LusASML2*, *LusWNK-1*, and *LusWRKY2*, was relatively stable and did not differ in different samples during the 2-year experimental period. Variability of *Lus10017628* and *LusAMLT* expression was especially pronounced in fiber flax cultivars analyzed during the 2 years, while several other genes (*LusBZIP5*, *LusGH17-3A*, and *LusGRAM*) showed similar tendency (differences in the expression depended on the year) in all analyzed samples. *LusDFL1* was downregulated in fiber flax cultivars compared to linseed cultivars and wild species, this was independent of the year, while *LusWAT1-R* showed the opposite tendency (Figure 2 and Supplementary Figure S3).

The expression level of some selected genes differed according to the year of fiber collecting. For example, the expression of 10 from 49 analyzed genes (*LusABH, LusCES101, LusCESA4, LusCXH20, LusCTL20, LusCTL2, LusCTL20, LusFLA11-1, LusKIN14H*, and *LusLTP3)* was different when comparing the results of 2018 and 2019. The remaining genes, on the contrary, showed a more stable level of expression (*LusCESA1* and *LusCESA3-B*; Figure 2 and Supplementary Figures S3, S4). The expression of *CESA* genes that encode various isoforms of cellulose synthases is an interesting example here. The expression of the *LusCESA4* gene that encodes glycosyltransferase involved in the synthesis of the secondary cell wall (Taylor et al., 2004), varied strongly in all studied flax samples (Figure 2), while *LusCESA1* and *LusCESA3-B* (involved in primary cell wall synthesis, Desprez et al., 2007) and *LusCESA8-A* (secondary cell wall synthesis, Taylor et al., 2004) genes did not show such differences (Figure 2 and Supplementary Figures S3, S4).

**FIGURE 3:**
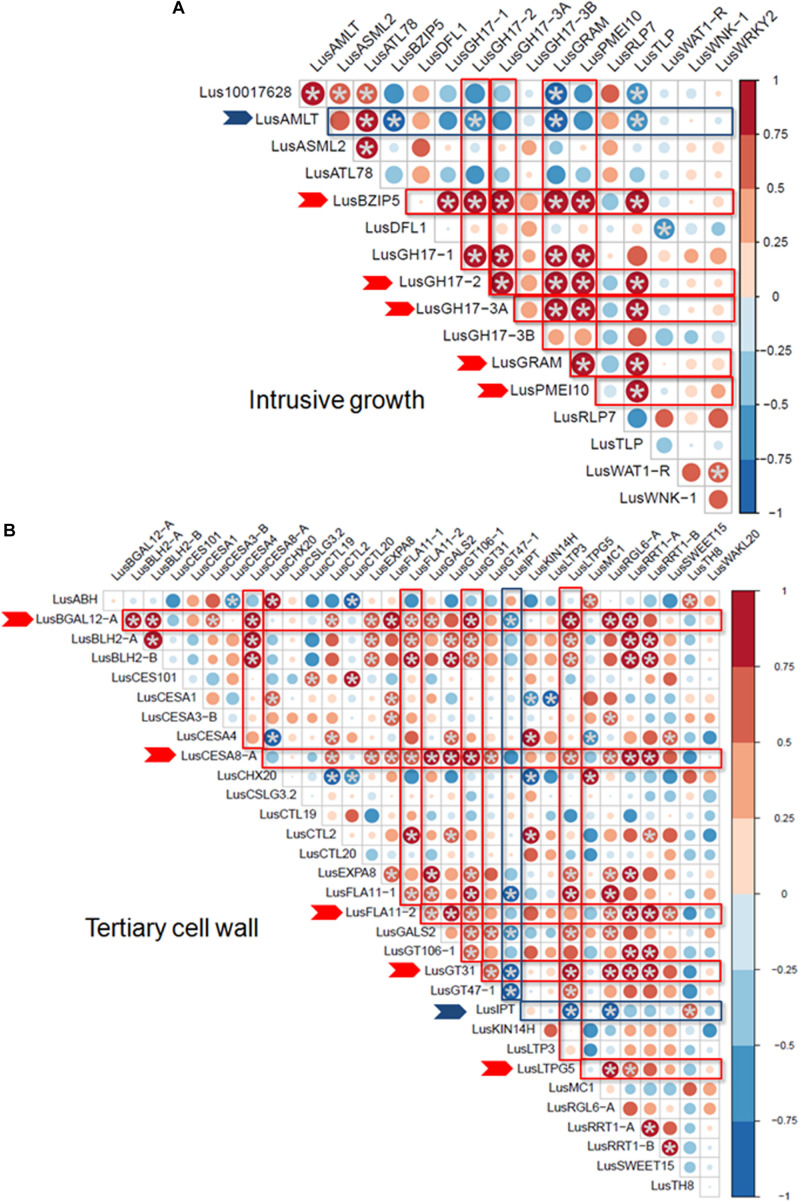
Co-expression of selected genes at the intrusive growth stage **(A)** and at TCW deposition **(B)** of flax bast fibers. Statistically significant points (*q*-value < 0.01) are marked by asterisks. Genes that are positively and negatively co-expressed with other genes are marked by red and blue arrows, respectively. The size of the dots indicates the absolute value of Pearson’s correlation coefficient.

**FIGURE 4:**
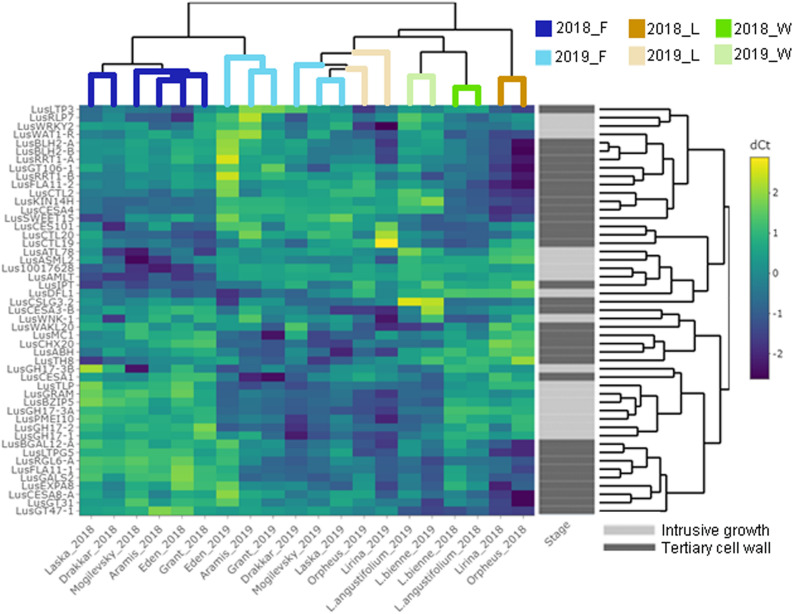
Hierarchical clustering and heatmap analysis of the genes that are specifically expressed in fibers at the intrusive stage and tertiary cell wall development. The heatmap was generated using ΔCq (normalized to the *LusGAPDH, LusETIF1*, and *LusETIF5A* genes) and scaled by rows. The upper dendrogram reflects the result of the sample clustering depending on the gene expression profiles. Blue lines = fiber flax (F), brown lines = linseed flax cultivars (L), green lines = wild species of flax (W). Bright colors indicate samples from 2018, pale colors are those for 2019. The dendrogram on the right represents gene clustering calculated based on their expression similarity in fibers isolated from different flax samples and at the two analyzed stages of development – intrusive growth and formation of the tertiary cell wall (designated by pale and bright gray colors, respectively).

Some of the selected genes were co-expressed (Figure 3). Among the genes upregulated during intrusive growth, *LusBZIP5, LusGH17s, LusGRAM*, and *LusPMEI10* were positively co-expressed with each other, while all of them were negatively co-expressed with *LusALMT12* (Figure 3A). *LusBGAL12-A, LusCESA8-A, LusFLA11-2, LusGT31*, and *LusLTPG5*, all genes that were upregulated during TCW formation, exhibited positive co-expression between each other and with other genes; all of them were negatively co-expressed with *LusIPT* (Figure 3B). Upon clustering of the samples on the base of gene expression, they were mainly combined in accordance with the FLW group (fiber, linseed, wild; Figure 4). Two major clusters were revealed independently from the year of sampling: almost all fiber flax cultivars were grouped together, while another cluster was formed by linseed cultivars with wild species and three fiber flax cultivars (Drakkar, Mogilevsky, and Laska) sampled in 2019. Notably, most of the samples collected through the 2 years were grouped together.

### Analysis of Parameters of Technical Fiber Quality in Members of the FLW Group

**FIGURE 5:**
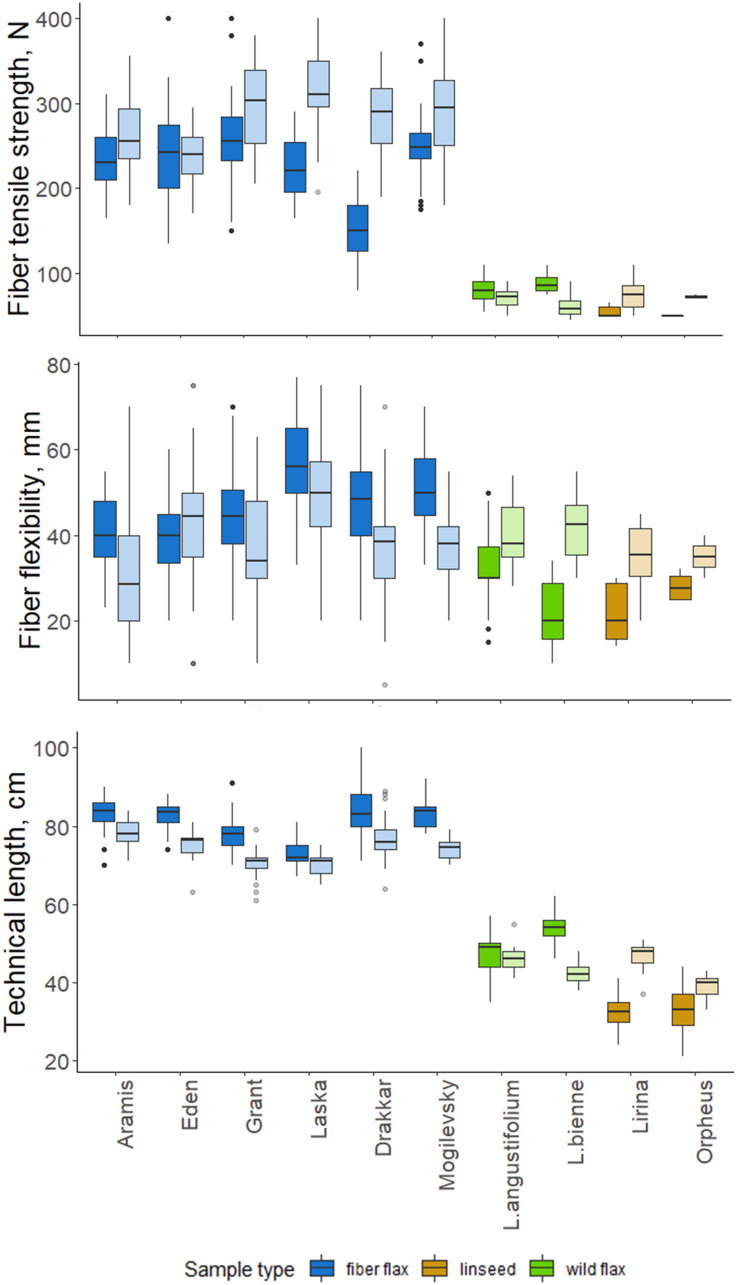
Quality features of scutched fibers for analyzed flax samples. The bottom and top of the box are the first and third quartiles, respectively; the bold line within the box is the median. The upper whisker extends from the third quartile to the largest value, but no further than 1.5 * IQR (interquartile range). The lower whisker extends from the first quartile to the smallest value at most 1.5 * IQR. “Outlying” points are plotted individually. Bright colors indicate samples from 2018, pale colors are those for 2019.

We evaluated the quality of FLW technical fibers harvested in 2018 and 2019. The fiber flax group differed significantly from other groups; the pronounced differences were also often detected between samples of the same cultivar collected in different years (*p*-value < 0.05; Figure 5).

Technical fibers scutched from fiber flax cultivars were notably stronger than those from linseed and wild flax (Figure 5). The strongest technical fibers were obtained from Laska and Grant in 2019 –315 and 298 N, respectively; while in linseed and wild species it was below 100 N. In 2019, the fiber tensile strength was higher than in 2018 in all members of the FLW group, except Eden and *L. angustifolium* which showed no difference.

Flexibility was less variable among FLW representatives than the tensile strength. It had a multidirectional trend change over the two experimental years in the different sample groups. Fiber flax cultivars produced more flexible fiber in 2018, and the remainder of the flax samples did so in 2019. The changes in Orpheus’s flexibility were statistically not significant (*p*-value = 0.081; Figure 5). The most flexible technical fiber was isolated from the stems of Laska cultivar in 2018 (56.2 mm).

The technical stem length was longer in 2018 than in 2019 for most fiber flax cultivars and *L. bienne* (Figure 5). Laska and *L. angustifolium* had a similar stem technical length in both experimental years. The technical stem length difference over the two experimental years can largely be accounted for different soil moisture levels during the period of fast growth. Soil moisture was assessed as sufficiently humid during the period of the fast growth of flax plants in 2018, and dry in 2019: Selyaninov Hydrothermal Coefficient (Taparauskienë and Miseckaite, 2017) was, respectively, 1.03 and 0.47 during the fast growth period as was determined by the Institute of Flax.

Altogether, a considerable range of fiber quality parameters was demonstrated in various representatives of the FLW group and in plants grown under different weather conditions. This range was further related to selected gene expression values.

### Regression Analysis

**FIGURE 6:**
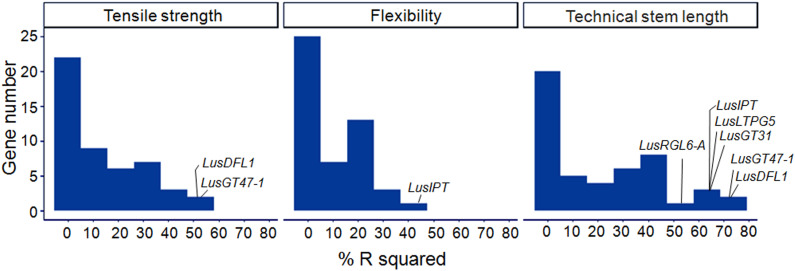
Distribution of *R*^2^ (%) in single gene regression models relating the fiber quality parameters (dependent variable) with ΔCq of the studied genes (independent variables). The genes with maximum scores are labeled for each quality parameter (tensile strength, flexibility, and technical stem length).

A linear regression was used to model each of the fiber quality parameters (dependent variable) with ΔCq of the studied genes (independent variables). The R-squared of the single-gene models ranged from 0.00 to 0.75 (Figure 6). The highest scores were obtained for *LusDFL1*, *LusGT47-1*, and *LusTH8* in relation to fiber tensile strength; for *LusIPT* in relation to fiber flexibility; and for eight genes, *LusGT47-1*, *LusDFL1*, *LusIPT*, *LusLTPG5*, *LusGT3*, *LusRGL6-A*, *LusBGAL12-A*, and *LusBLH2-A* in relation to technical stem length (Supplementary Figure S5). In general, the studied genes were able to explain the technical length of stem and fiber tensile strength better than the flexibility (Figure 6).

Two alternative regularization methods were used to produce multiple regression models of the fiber quality features. The first one is the LASSO method that performs selection of a small set of highly predictive features. The second one is the stepwise forward-selection, which uses more features and fits a more predictive model on the expense of higher bias (Hastie et al., 2017). LASSO models of “tensile strength” and “technical stem length” included two independent variables – *LusGT47-1* and *LusDFL1*, together accounting for 58 and 80% of the variability of the respective parameters (Table 3). The expression level of the *LusTH8* gene also showed relatively high value of the determination coefficient in single-gene regression models (Supplementary Figures S5H,K), but did not meet the specified statistical parameters of the LASSO-analysis (Supplementary Figure S6). The model of the tensile strength obtained by stepwise forward selection contained twelve independent variables (Table 3 and Supplementary Figure S7) and could explain almost 97% of variation in the tensile strength. The stepwise forward selection model of “technical stem length” included five independent variances and could explain 94% of the technical length variation (Table 3 and Supplementary Figure S7). *LusIPT* was the only gene included in the LASSO model of “flexibility” (Supplementary Figure S6). The stepwise forward-selection model of this parameter included four explanatory genes (Table 3), which together explained 60% of the flexibility dispersion.

**TABLE 3:** Linear regressions of scutched fiber quality features on studied gene relative expression levels.

Quality feature	Independent variable	Adjusted *R*^2^	*p*-Value	Method of regression regularization
Tensile strength	*LusDFL1, LusGT47-1*	0.58	2.3E-04	LASSO
	*LusDFL1, LusTH8, LusWNK-1, LusSWEET15, LusCESA4, LusLTP3, LusCTL2, LusABH, LusCTL19, LusCESA8-A, LusFLA11-2, LusCESA1*	0.97	8.8E-06	Stepwise
Flexibility	*LusIPT*	0.42	2.1E-03	LASSO
	*LusIPT, LusKIN14H, LusAMLT, LusDFL1*	0.58	1.6E-03	Stepwise
Technical stem length	*LusDFL1, LusGT47-1*	0.80	4.2E-07	LASSO
	*LusGT47-1, LusWRKY2, LusLTP3, LusWAKL20, LusLTPG5*	0.94	5.9E-09	Stepwise

### Chromosome Localization of the Studied Genes and Polymorphism in Their Coding Sequences

Polymorphic variants of genes can be valuable source material for flax breeding. To check for the presence of polymorphism in coding regions of genes expressed in fibers, RNA-Seq analysis of transcriptomes was performed for fibers isolated from the members of each FLW group, namely fiber flax Grant, linseed Lirina, and wild flax *L. bienne*. We analyzed transcriptomes of isolated fibers collected at the advanced stage of fiber specialization and therefore compared coding sequences (CDS) of the selected 32 TCW-upregulated genes with the reference flax genome. We did not consider genes that were upregulated during fiber intrusive growth, because they are poorly expressed at this stage of fiber development.

We found 176 SNPs for which at least one of the replicates showed variation from the reference (Supplementary Table S3). The CDS of eight genes (*LusMC1, LusCTL19, LusCTL20, LusGT31, LusWAKL20, LusFLA11-1, LusFLA11-2*, and *LusLTPG5*) were identical to the reference genome. At least one SNP in at least one biological replicate was present in the sequences of the remaining genes. These variations could arise both from the SNPs of the individual plants and from the inherent errors of the reverse transcriptase reaction (Ji and Loeb, 1992). Only genes that met the SNP-calling criteria in all three biological replicates of each sample were considered as polymorphic (Table 4). Ten such genes were revealed having 42 SNPs in total. The distribution of SNPs between the genes was not uniform. The largest number of SNPs was identified in *LusCESA3-B* (11 SNPs, CDS length 3279 bp) and *LusCSLG3.2* (9 SNPs, CDS length 2232 bp). One SNP per gene was found in *LusCES101* (2250 bp), *LusGT47-1* (1716 bp), *LusBLH2-A* (2229 bp), and *LusCTL2* (993 bp). Thus, the level of CDS polymorphisms in analyzed genes varied from 3.4 SNPs per 1 Kbp in *LusCESA3-B* to zero in the eight genes listed above.

**TABLE 4:** SNPs detected in coding sequences of TCW-upregulated genes.

Gene name	Gene ID	Scaffold	Scaffold position	SNP	Samples with SNP	CDS position	Triplet substitution	AA substitution
*LusCESA8-A*	Lus10007296.g	scaffold859	119591	t>c	*L. bienne*, Grant	c.A2920G	auu>guu	Ile>Val
			120290	g>a	*L. bienne*, Grant	c.C2322T	auc>auu	Silent
			121671	a>g	*L. bienne*, Grant	c.T1308C	ggu>ggc	Silent
			121731	g>a	*L. bienne*, Grant	c.C1248T	cgc>cgu	Silent
			122013	c>t	*L. bienne*, Grant	c.G966A	ccg>cca	Silent
			122508	a>c	*L. bienne*, Grant	c.T546G	guu>gug	Silent
			123246	g>a	*L. bienne*, Grant	c.C165T	agc>agu	Silent
*LusCESA3-B*	Lus10007538.g	scaffold259	26161	c>t	*L. bienne*, Grant, Lirina	c.C162T	agc>agu	Silent
			26194	t>a	*L. bienne*, Grant, Lirina	c.T195A	ccu>cca	Silent
			28415	g>a	*L. bienne*, Grant, Lirina	c.G1467A	gag>gaa	Silent
			28850	g>a	*L. bienne*, Grant, Lirina	c.G1699A	ccg>cca	Silent
			28964	c>t	*L. bienne*, Grant, Lirina	c.C1812T	uuc>uuu	Silent
			29573	t>c	*L. bienne*, Grant, Lirina	c.T2151C	uuu>uuc	Silent
			29938	c>t	*L. bienne*, Grant, Lirina	c.C2361T	ggc>ggu	Silent
			29983	c>t	*L. bienne*, Grant, Lirina	c.C2406T	cgc>cgu	Silent
			30043	t>c	*L. bienne*, Grant, Lirina	c.T2466C	auu>auc	Silent
			30163	a>g	Lirina	c.A2586G	aga>agg	Silent
			30166	t>c	*L. bienne*, Grant, Lirina	c.T2589C	uuu>uuc	Silent
*LusCES101*	Lus10009582.g	scaffold1331	314229	t>c	*L. bienne*, Grant, Lirina	c.A1879G	aau>gau	Asn>Asp
*LusGT47-1*	Lus10013790.g	scaffold1168	492105	g>t	*L. bienne*, Grant, Lirina	c.G1108T	guc>uuc	Val>Phe
*LusBLH2-B*	Lus10016790.g	scaffold903	514884	g>a	*L. bienne*, Grant, Lirina	c.C948T	gac>gau	Silent
			514893	g>c	*L. bienne*, Grant, Lirina	c.C939G	ggc>ggg	Silent
			515143	t>g	*L. bienne*, Grant, Lirina	c.A689C	gac>gcc	Asp>Ala
			515193	t>a	*L. bienne*, Grant, Lirina	c.A639T	gca>gcu	Silent
*LusKIN14H*	Lus10020682.g	scaffold303	171179	c>a	Lirina	c.G2446T	gcu>ucu	Ala>Ser
			171294	c>g	*L. bienne*, Grant, Lirina	c.G2331C	ugg>ugc	Trp>Cys
			171309	t>c	*L. bienne*, Grant, Lirina	c.A2316G	aca>acg	Silent
			171607	a>g	*L. bienne*, Grant, Lirina	c.T2130C	cau>cac	Silent
			172853	a>g	Lirina	c.T1432C	uug>cug	Silent
*LusBLH2-A*	Lus10022485.g	scaffold38	778141	a>g	*L. bienne*, Grant	c.T1726C	ucg>ccg	Ser>Pro
*LusCSLG3.2*	Lus10023057.g	scaffold325	175435	g>t	*L. bienne*, Grant	c.C1774A	ccc>acc	Pro>Thr
			176191	t>a	*L. bienne*, Grant	c.A1473T	aua>auu	Silent
			177347	a>t	*L. bienne*, Grant, Lirina	c.T963A	gcu>gca	Silent
			177784	t>c	*L. bienne*, Grant, Lirina	c.A738G	caa>cag	Silent
			177823	g>a	*L. bienne*, Grant, Lirina	c.C699T	aac>aau	Silent
			177865	t>g	*L. bienne*, Grant, Lirina	c.A657C	aga>agc	Arg>Ser
			179212	t>g	Grant, Lirina	c.A50C	aag>acg	Lys>Thr
			179226	g>a	Grant, Lirina	c.C36T	ccc>ucu	Pro>Ser
			179228	g>a	Grant, Lirina	c.C34T		
*LusCTL2*	Lus10037737.g	scaffold196	1324196	c>t	*L. bienne*, Grant, Lirina	c.G136A	gug>aug	Val>Met
*LusGALS2*	Lus10038387.g	scaffold28	1041978	t>c	*L. bienne*, Grant, Lirina	c.835G	acc>gcc	Thr>Ala
			1042130	t>g	*L. bienne*	c.A683C	caa>cca	Gln>Pro

Members of the FLW group had various degrees of SNP specificity (Table 4). Three specific SNPs (2 SNPs in *LusKIN14H* and 1 SNP in *LusCESA3-B*) were found in Lirina, and one specific SNP (in *LusGALS2*) in *L. bienne*. Specific SNPs were not found for Grant: all polymorphisms found in the CDS of Grant cultivar genes were also found in *L. bienne* (5 SNPs in *LusCESA8-A*, 2 SNPs in *LusCSLG3.2*, and 1 SNP in *LusBLH2-A*), Lirina (3 SNPs in *LusCSLG3.2*), or *L. bienne* and Lirina combined (25 SNPs) (Table 4).

Most discovered point mutations did not cause amino acid substitutions in the primary peptide structure due to redundancy in the genetic code. For instance, all SNPs identified in the *LusCESA3-B* gene were silent mutations. The largest number of missense mutations (four SNPs) was found in the *LusCSLG3.2* gene (Table 4). The *in silico* prediction of functional effect of sequence variants with SNAP2 tool^8^ indicated that non-synonymous SNPs (P592T, K17T, P12S; data not shown) found in the protein encoded by *LusCSLG3.2* are most likely not deleterious. According to another *in silico* prediction tool^9^, these three amino acids are likely to be buried rather than exposed on the protein surface, meaning that these residues are more likely to affect the protein conformation but not the binding with other proteins or ligands. One missense mutation was identified in each of *LusCESA8-A* and *LusGT47-1* (Table 4). This fact may be of practical interest since the expression levels of these genes are included as independent variables in regression models for fiber quality feature.

Next, we anchored the selected genes into the physical map of flax chromosomes in order to assess their potential linkage with mapped markers (Supplementary Table S4). We used chromosome-scale pseudomolecules (You et al., 2018), as well as gene annotation from the phytozome.gov portal. The empirically established genome wide ratio of 239 Kb/cM was used to translate physical distance (in bp) into genetic distance (in cM) (Cloutier et al., 2012). We took into account the gene sequence orientation inside the scaffold, and also scaffold orientation inside the chromosome pseudomolecules when physical distance was calculated. We also corrected physical distance for genes lying on scaffolds split into several parts (You et al., 2018; Supplementary Table S4).

The largest number of studied genes (seven genes) were anchored onto the third chromosome, no genes were mapped onto chromosome 5 and 10 (Supplementary Table S4). The distance between the markers and the mapped genes varied from 0.007 cM (*LusWAKL20*) to 7.437 cM (*LusSWEET15*) owing to the uneven marker saturation of the genetic map. The *LusSWEET15* and *LusASML* genes were located in chromosomes sites least covered by markers. The distances to the nearest mapped marker were 7.437 and 4.609 cM, respectively. The closest to each other genes, *LusGT31*, *LusCTL20*, and *LusCTL19*, were located on the eighth chromosome (Supplementary Table S4).

## Discussion

### Approaches to Find Candidate Genes for Manipulation of Fiber Quality Parameters

Breeders are urgently tasked to clarify molecular links between genetic background and economically valuable crop traits. Bast fiber quality is a complex feature that includes a number of mechanical and physical parameters of fiber bundles that are measured after crop harvest. These parameters are influenced by the post-harvest processing of stems, but are largely determined during the course of plant development by the reached length of individual fibers, number of fibers and their bundles on a stem cross-section, and the width and composition of the cell wall (Mokshina et al., 2018). These biological parameters are determined by a complex combination of environmental biotic and abiotic factors as well as the genetic status of the plant. Differences in final fiber properties between flax cultivars and species are largely determined by genetic background; this creates a need to identify important genetic elements that can be used in crop quality improvement programs.

Genetic data have been used during different approaches toward improving industrial crops. One of these is the identification of quantitative trait loci (QTL) (Collard et al., 2005). QTLs associated with lint percentage have been identified and mapped for cotton, resulting in the development of simple sequence repeat (SSR) markers for the control of their inheritance (Abdurakhmonov et al., 2007). A promising example of QTL utilization for flax breeding is the model of pasmo (a fungal disease) resistance prediction (He et al., 2019). The developed model includes 500 QTLs as independent variables. Although the determination coefficient of individual QTLs does not exceed 0.25, the model itself has a very high accuracy of pasmo resistance prediction (He et al., 2019). For linseed, SSR markers related to parameters of oil production have been revealed (Soto-Cerda et al., 2014). However, QTL mapping has not yet been successfully used in the identification of flax fiber quality and yield traits. A significant association has been established with the “plant height” trait for two molecular markers in flax: *LuFAD3B* (encodes desaturases capable of desaturating linoleic acid) (*R*^2^ = 0.117, *p* < 0.01) and *CV8824* (encodes leucyl aminopeptidase) (*R*^2^ = 0.095, *p* < 0.01) (Nandy, 2012). Low coefficients of the determination indicate that these markers explain a small part of the variation in the phenotypic “plant height” trait. Genome-Wide Association Study (GWAS) has also not been very effective in fiber flax agronomic studies since few genes could be associated with important agronomic traits of fiber flax (Xie et al., 2018). *Lus10016354* that encodes a xanthoxin dehydrogenase potentially involved in the synthesis of abscisic acid has been suggested as a causal gene for fiber percentage in flax species and *Lus10016125* encoding ABC transporter (ATP-binding cassette transporters) is speculated to be the candidate gene for flax plant height. The candidate genes from the detected two loci related to technical length could not be determined under the applied GWAS approach (Xie et al., 2018).

The current paper presents a more targeted functional approach based on the analysis of gene expression during specific stages of bast fiber development. The whole pipeline includes the identification of temporal and spatial localization of fiber intrusive elongation and TCW deposition in the stems of developing flax plants (Gorshkova et al., 2003, 2004), transcriptomic analysis of fibers at certain developmental stage (Gorshkov et al., 2017; Gorshkova et al., 2018b), identification of genes with fiber-specific expression by determination of tau score (Mokshina et al., 2020), and the relation of fiber quality parameters to the expression of genes important at key stages of bast fiber development (current paper). The identification of genes contributing to fiber intrusive elongation and TCW deposition is an important step in this pipeline because it reduces information noise in the analysis and determines the effectiveness of the subsequent steps.

Fully understanding that gene expression may not have a direct relation to the activity of the encoded protein owing to various post-translational modifications and the other means of enzyme activity modulation, we checked for the presence of genes with expression levels in developing plants that were highly related to final crop traits. As an argument for such an approach, comparison of various flax genotypes indicated that some of the selected genes kept a relative level of expression (normalized to the reference genes) in accordance to the FLW group despite the different conditions throughout the 2-year experimental period (Figure 2 and Supplementary Figure S3). This was confirmed by clusterization performed for the expression data of all analyzed genes obtained for the 2 years of observation (Figure 4).

Studies that attempt to find a correlation between gene expression and plant traits have been stimulated by the development of transcriptome technology. For example, correlation analysis of growing leaf transcriptome with mature leaf parameters has been performed in a maize recombinant inbred line population by Baute et al. (2015). Hundreds of transcripts putatively linked with 17 traits of potato, representing both well-known and novel associations were described based on gene expression level (Alexandersson et al., 2020). In a study devoted to cotton fiber (trichome) quality, the expression of some genes was evaluated by qPCR. Inbred lines of *Gossypium* species of higher and lower quality showed a correlation between the expression of a number of genes and fiber (trichome) quality: the expression of the *cinnamate-4-hydroxylase* gene was correlated with length uniformity index (*R*^2^ = 0.73), the expression of the *Lim domain* gene was correlated with mean length (*R*^2^ = 0.68), and the expression of the *pectin methylesterase* gene was correlated with micronaire (resistance to airflow per unit mass of fiber) (*R*^2^ = 0.55) (Al-Ghazi et al., 2009). Our approach helped to effectively identify flax genes whose expression level highly correlated to bast fiber strength, flexibility, and technical length.

### Genes Whose Expression Levels Are Highly Related to the Fiber Quality Parameters: Possible Functions of the Encoded Products

The set of genes whose expression is highly related to quality parameters includes those genes upregulated both at fiber intrusive elongation and TCW deposition stages, with both positive and negative correlation coefficients (Supplementary Figure S5). Genes included in the multiple regression models (Table 3) belonged to different co-expression groups (Figure 3). This may indicate the participation of these gene products in various biochemical processes that are important for the formation of high-quality fiber.

The optimal model of the tensile strength obtained by the stepwise method contained 12 genes (Table 3 and Supplementary Figure S7): *LusDFL1, LusTH8, LusWNK-1, LusSWEET15, LusCESA4, LusLTP3, LusCTL2, LusABH, LusCTL19, LusCESA8-A, LusFLA11-2, LusCESA1*. Three of them are genes for cellulose synthases, including *CESA1* that is considered as specific for primary cell wall and those for secondary cell wall (*CESA8, CESA4*). All these genes are activated in fibers during TCW deposition (Mokshina et al., 2017). Also, several potential co-factors (or closely related proteins) for cellulose synthase complex activity are in the list, such as *LusLTP3, LusCTL2*, *LusFLA11-2; LusCTL19.* The products of these genes can add specific details to the process of cellulose deposition during formation of TCW in phloem fibers. The precise role of FLA proteins and CTL proteins in cellulose biosynthesis is not established yet, but several studies confirmed that some isoforms of these proteins are involved in cell wall biosynthesis (MacMillan et al., 2010; Sánchez-Rodríguez et al., 2012; Huang et al., 2013; Guerriero et al., 2017). Flax LusLTP3 is predicted to be a 119 amino acid protein; it shows a high homology with the AtLTP3 protein encoded by *AT5G59320*. A common feature of LTP is their ability to bind and transfer lipids (Finkina et al., 2016), and individual isoforms likely play specific or multiple roles in important biological processes (Yeats and Rose, 2008). LTPs have been found to impact cell wall loosening and promotion of cell extension under tension in tobacco and wheat plants (Nieuwland et al., 2005). LTP3 of cotton (GenBank accession #AF228333) is reported to interact with the zinc-binding domain of cellulose-synthase GhCESA1 (Doblin et al., 2002). Cell wall localization (presence of signal peptide) was predicted for LusLTP3 by TargetP^10^. In hemp, *LTP3* gene was also activated in the stem region, containing fibers forming TCW as compared to the upper part of the stem with fibers at an earlier stage of development (Guerriero et al., 2017). The role of *LusWNK-1* [serine/threonine-protein kinase WNK (with no lysine)-like protein, ortholog to AT1G60060] from the group of genes upregulated at fiber intrusive growth is difficult to predict, since such protein kinases modulate a wide spectrum of cellular processes through phosphorylating downstream protein substrates (Cao-Pham et al., 2018).

*LusDFL1* (*Lus10003598*), expressed at the stage of fiber intrusive growth, was included as the independent variable in regression models that explained each of three studied fiber quality parameters. *LusDFL1* is the ortholog of the *AtDFL1* gene (*DFL1*, *DWARF IN LIGHT 1*; *GH3.6, Gretchen Hagen 3.6*, *AT5G54510*) that encodes an IAA-amido synthetase, which conjugates Ala, Asp, Phe, and Trp to auxin (Staswick et al., 2005). AtDFL1 is involved in auxin signal transduction and inhibits shoot and hypocotyl cell elongation and lateral root cell differentiation under light (Nakazawa et al., 2001). *Arabidopsis* lines overexpressing this gene accumulate IAA-Asp and are hypersensitive to several auxins. Over-expression of antisense *DFL1* results in larger shoots and an increase in the number of lateral roots. *LusDFL1* has increased expression in oilseed flax cultivars, as well as in wild flax species; this may cause a higher activity of IAA-amido synthetase and lead to faster inactivation of phytohormones in the bast fibers of these genotypes. Lower auxin conjugation may result in longer fibers in fiber flax cultivars, as compared to the remainder of the FLW group (Supplementary Figure S2), leading to higher scutched fiber strength. Of note, putative IAA-amido synthetases GH3.9 (four isoforms) were activated in the fiber-containing bark peeled from elongating internode of ramie, as compared to fully elongated internode (Xie et al., 2020). Enhanced expression of *GH3* genes in Arabidopsis leads to dwarf plants and de-etiolation in darkness (Hagen and Guilfoyle, 2002). According to study devoted to expression of *GH3* genes in cotton, expression of some *GhGH3* genes may play a role in cotton seed hair initiation and development by regulating the IAA content of ovules (Yu et al., 2018). One more potentially hormone-related gene in the list was *LusABH* (ortholog to *AT1G52700*) encoding alpha/beta hydrolase that belongs to the one of the largest groups of enzymes with diverse catalytic function (Holmquist, 2000); In various species, some ABH domain proteins have been characterized and shown to play roles in lipid homeostasis (Lord et al., 2013). Among other functions, it was suggested that ABH fold serves as the core structure for hormone receptors in the gibberellin, strigolactone, and karrikin signaling pathways in plants (Mindrebo et al., 2016).

Co-expression analysis showed the significant negative correlation of *LusDFL1* with *LusWAT1-R* (walls are thin1-related proteins) (Figure 3A). *AtWAT1* encodes a tonoplast-localized auxin transporter, which facilitates auxin export from vacuoles to the cytoplasm (Ranocha et al., 2013). In *Arabidopsis*, it is required for secondary wall formation in fibers (Ranocha et al., 2010). Gene for WAT1 was also up-regulated in hemp tissues, at the advanced stage of fiber development (Guerriero et al., 2017). In ramie stem, several genes for WAT1-related proteins were up-regulated in the bark of elongating internode as compared to non-elongating one (Xie et al., 2020). Thus, two genes encoding proteins involved in keeping auxin sub-cellular homeostasis and specifically upregulated at flax fiber intrusive elongation are in the list of genes whose expression is highly related to the quality of technical fibers.

The *LusSWEET15* gene was related with the tensile strength, it is the orthologous gene of *ATSWEET15* (*AT5G13170*, known as *SAG29*). The SWEET family (Sugar Will Eventually be Exported Transporters) includes plant transmembrane proteins that bidirectionally transport mono- and disaccharides across the membrane along the substrate concentration gradient (Jeena et al., 2019). These transporters are known as key proteins for improving crop stress tolerance and yield through their involvement in carbohydrate partitioning (Wei et al., 2019). Upregulation of *SWEET15* has been demonstrated for seeds and flowers in several plant species (Chen et al., 2015; Wang et al., 2019), as well as in *Arabidopsis* shoots under osmotic stress (Durand et al., 2018). Flax phloem fibers are living cells with quite active photosynthesis, as indicated by chloroplast functional ultrastructure (Salnikov et al., 2008) and enrichment with transcripts associated with photosynthesis (Gorshkov et al., 2017). At the same time, they are powerful sinks of assimilates due to the active deposition of the cell wall. The activation of sugar transporters may be critical for carbohydrate turnover, balancing of assimilates, and maintenance of osmotic status, all of which accompany TCW deposition in bast fibers. The analysis of cell wall composition and transcriptomics in stem tissues of stinging nettle (*Urtica dioica* L.) revealed up-regulation of *SWEET15* gene in the cortical tissues sampled at the bottom; it was proposed that product of this gene could be involved in the transfer of sucrose to the thickening bast fibers (Xu et al., 2019).

*LusTH8* (ortholog to *AT1G69880*) could be considered as potential marker of fiber quality; its expression has a high negative correlation with fiber quality parameters (Supplementary Figures S5H,K). In accordance, fiber flax cultivars had a lower expression level of *LusTH8* compared to linseed cultivars and wild species (Supplementary Figure S4). Thioredoxins are small and powerful disulfide reductases (Holmgren, 1985), they act as hydrogen donors for redox enzymes (Laurent et al., 1964) through regulating a growing number of biological processes. Through the reduction of intramolecular disulfide bonds, thioredoxin H has been shown to promote the degradation of reserve substances during seed development (Yano et al., 2001). Suppression of *Trx h9* expression in wheat promotes retardation of germination and delayed or decreased expression of the associated enzymes, leading to suppression of preharvest sprouting (Li et al., 2009). It may be surmised that *LusTH8* upregulation in fibers of linseed cultivars and wild species can promote maturation of these cells, while in fiber flax cultivars their prolonged development is necessary for thicker cell wall formation. Another possible role of thioredoxins may be associated with signal transduction and induction of TCW formation. In poplar stems, 36% of identified proteins that were differentially expressed after gravistimulation (i.e., induction of TCW deposition in xylem fibers) have been reported as potential thioredoxin targets; the involvement of thioredoxin H in the first events of signal transduction in inclined poplar stems has been suggested (Azri et al., 2013). Though the expression level of *LusTH8* is demonstrated to be highly related to technical fiber quality, the exact function of *LusTH8* in bast fibers is still unknown.

A list of genes that showed the high value of the adjusted coefficient of determination in the model using technical stem length as the dependent variable included *LusGT47-1, LusWRKY2, LusLTP3, LusWAKL20, LusLTPG5*. The LusGT47-1 gene encodes a glycosyltransferase, which is an enzyme that could potentially be associated with RG-I synthesis (Gorshkov et al., 2017), and it is included in a co-expression network with genes that are likely also related to RG-I biosynthesis and modification (*LusGT31, LusGALS2, LusBGAL12-A, LusRRT1-A*, and *LusRRT1-B*; Figure 3B). Probable glycosyltransferase homologous to *At5g03795* (*GT47*) was activated in ramie bark from fully elongated internode region comparing to bark from elongating internode), as well as a number of galactosidases *BGALs* (Xie et al., 2020). *LusWRKY2* is ortholog to *At5g56270* that encodes WRKY transcription factor 2, a zinc-finger protein (Chen et al., 2017). Interestingly, the ortholog of WRKY2 in poplar was activated in tension wood samples that formed TCW in xylem fibers (Andersson-Gunnerås et al., 2006). Using transgenic tobacco plants constitutively over-expressing the *Vitis vinifera* L. transcription factor VvWRKY2 it was demonstrated that WRKY2 can modulate expression of genes involved in lignin biosynthesis pathway and cell wall formation (Guillaumie et al., 2010). Thus, this transcription factor could be prominent candidate for regulator involved in thickening of fiber cell wall. LusWAKL20, cell wall-associated receptor kinase-like 20, serine/threonine-protein kinase that may function as a signaling receptor of extracellular matrix component (Verica and He, 2002), but exact function in TCW needs to be elucidated in further.

The expression level of the *LusIPT* gene (*Lus10028015*) was related to the fiber flexibility and also had a high score in the stepwise regression model for technical stem length (Table 3 and Supplementary Figures S5C,O). This gene (orthologous to *AT3G63110*, *IPT3*) encodes the adenylate isopentenyltransferase (cytokinin synthase), which is involved in cytokinin biosynthesis (Takei et al., 2001). Among *Arabidopsis IPTs*, *AtIPT3* is expressed in the vasculature throughout the plant (Miyawaki et al., 2004). The promoter activity of *AtIPT3* is high in the phloem but not in the cambium during secondary growth in *Arabidopsis* plants (Matsumoto-Kitano et al., 2008). For hemp fibers forming TCW, it was speculated that cytokinins regulate the initiation of lignin deposition in secondary tissues, while later lignification is tuned by other factors (Behr et al., 2016). According to our data, *IPT3* was activated in flax fibers during deposition of TCW that lacks lignin; the role of cytokinin in fiber development needs to be better characterized.

Several described genes (*LusTH8, LusLTP3*, and *LusIPT*) are specifically expressed in developing bast fibers as indicated by a high tau-score (Mokshina et al., 2020). This means that manipulation of their activity through conventional breeding or genome editing would have limited effect on cells other than fibers.

Based on the regression analysis and the biological function investigation we may provide a short list of genes with high potential to influence the technical fiber quality (Table 5).

**TABLE 5:** The most promising genes regarding the manipulation of technical fiber quality as revealed in the current study.

Gene name	Gene ID	Preferential expression	Biological function	Impact on technical fibers properties	Product localization*
*LusDFL1* IAA-amido synthetase	Lus10003598	Intrusively growing fiber	Phytohormone biosynthesis and signal transduction	Tensile strength; technical stem length	Cytoplasm, expected accuracy 84%
*LusIPT* Isopentenyl transferase	Lus10028015	TCW depositing fiber	Phytohormone biosynthesis and signal transduction	Flexibility	Chloroplast, expected accuracy 89%
*LusGT47-1* Glycosyl transferase GT47	Lus10013790	TCW depositing fiber	Rhamnogalacturonan I biosynthesis	Tensile strength; technical stem length	Golgi apparatus membrane, expected accuracy 88%

*According to LocTree3^11^ (Goldberg et al., 2014).

### Analysis of SNPs Using RNA-Seq Reveals a Highly Variable Level of Polymorphism in Coding Sequences of Genes Important for Fiber Development

To incorporate those genes in which expression is highly related to fiber quality into breeding programs, it is important to understand their polymorphism in various flax genotypes. Genome-wide SNP analysis in flax through next generation sequencing was performed by Kumar et al. (2012). In the latter study, SNP discovery was performed through *in silico* analysis of the sequencing data for eight flax genotypes against the whole genome shotgun sequence assembly of flax genotype CDC Bethune. It was shown that 25% of the found SNPs were present in genic regions, of which only 8% were present in coding regions (Kumar et al., 2012).

According to the current data, 25% of the studied genes were totally devoid of polymorphism when compared to the reference genome. In 31% of the analyzed genes, nucleotide sequence variations were reliably identified as present in all three replicates. The identified SNPs were irregularly distributed through the CDS of the studied genes. The highest SNP range was revealed in *LusCESA3-B* CDS (3.4 SNPs per 1 Kbp). Such SNP frequency was significantly higher than in the genome-wide average; the latter varies, according to different investigations, from 0.17 SNP/Kbp (Kumar et al., 2012) to 1.2 SNP/Kbp (Xie et al., 2018). The average frequency of SNPs detected in CDS of all analyzed genes and present in all three replicates was 0.86 SNP/Kbp. Of note was the absence of polymorphism sites specific for the fiber flax cultivar (Table 4). Higher polymorphism in linseed supports the idea that flax was first domesticated for oil, rather than fiber (Allaby et al., 2005).

Many of the revealed CDS variations were not reflected in protein sequences due to redundancy in the genetic code. Those that do result in amino acid substitutions are most likely not deleterious since they were found in phenotypically normal plants. Unfortunately, detailed analysis of the effects of the reported SNPs on the proteins is not possible due to a lack of resolved protein structures and, in some cases, even full amino acid sequences. It can also be hypothesized that the differences in fiber quality related to gene expression in the FLW group are determined by regulation of gene expression via specific transcription factors and other regulatory mechanisms (such as miRNA or methylation sites) rather than by the structure of the encoded proteins. A search for the regulatory elements will be the focus of future studies.

## Conclusion

Modern plant breeding has embraced many aspects of biological engineering, and it is ready to adopt modern genetic knowledge and approaches. New super-domesticated cultivars that could not be selected under natural environmental conditions or developed by common breeding techniques are now gaining a footing in the agricultural industry (Pérez-de-Castro et al., 2012). This paper proposed an approach that establishes a link between genetic background and valuable crop traits, the key step of which was the identification of genes with tissue and stage characters of expression. Identification was performed using transcriptome data analysis, which allowed the identification of flax genes that were highly correlated to bast fiber strength, flexibility, and technical length.

Based on linear regression modeling of quality traits with gene expression, genes that could be considered as potential markers for flax fiber quality were identified; among them, genes for products, involved in phytohormone biosynthesis and signal transduction (*LusDFL1, LusABH, LusIPT*, and *LusTH8*), lipid binding and transport (*LusLTP3* and *LusLTPG5*), rhamnogalacturonan I biosynthesis and modification (*LusGT47-1*), cellulose deposition *(LusCESA1, LusCESA4*, *LusCESA8-A, LusFLA11-2, LusCTL2*, and *LusCTL19*), carbohydrate (*LusSWEET15*) and malat (*LusAMLT*) transport, protein phosphorylation and cell signaling (*LusWRKY2, LusWAKL20*, and *LusWNK-1*) were revealed. Taking into account that these genes are specifically expressed in fibers, their active manipulation through conventional breeding or genome editing could impact bast fiber quality without affecting other tissues. Two missense point mutations were detected in genes included in the regression models (c.A2920G of *LusCESA8-A* and c.G1108T of *LusGT47-1*); these can be useful for flax breeding purposes. Thus, the algorithm to reveal and characterize those genes whose expression is important for certain fiber quality parameters was established, and this may be used in future breeding and genome editing programs.

## Data Availability Statement

RNA seq data were deposited in the NCBI Sequence Read Archive (SRA) under accession number: PRJNA658125.

## Author Contributions

DG, NM, and TG planned and designed the experiments. DG, TP, and MA implemented the study and acquired the data. DG, TP, and NM prepared figures and tables. DG, NM, TP, and TG drafted the manuscript. VB and AK helped to revise the manuscript. All authors read and approved the final manuscript.

## Conflict of Interest

The authors declare that the research was conducted in the absence of any commercial or financial relationships that could be construed as a potential conflict of interest.

## Footnotes

1 https://textileexchange.org/

2 http://www.fao.org/home/en/

3 https://sourceforge.net/projects/bbmap

4 https://genome.jgi.doe.gov/portal/pages/dynamicOrganismDownload.jsf?organism=Lusitatissimum

5 https://en.novogene.com/

6 http://dkoboldt.github.io/varscan/using-varscan.html

7 https://phytozome.jgi.doe.gov/pz/portal.html

8 https://rostlab.org/services/snap2web/

9 https://predictprotein.org/

10 http://www.cbs.dtu.dk/services/TargetP/

11 https://rostlab.org/services/loctree3/results.php?id=29faa499-389e-4436-91e7-c938ee161b60

## Supplementary Material

Supplementary Figure 1 Validation of qPCR data for 32 genes (TCW-upregulated) using RNA-Seq data.

Supplementary Figure 2 The distribution of elementary fiber length **(A)** and average length of individual fibers **(B)** in representatives of FLW groups.

Supplementary Figure 3 The relative expression levels (qPCR) of each studied gene (in alphabetical order) in all analyzed flax genotypes.

Supplementary Figure 4 The relative expression level of each studied gene (qPCR), averaged for three groups of flax genotypes (fiber cultivars, linseed cultivars, wild species).

Supplementary Figure 5 The relation between the technical stem length, the technical fiber strength, and flexibility and the expression level (ΔCq-value) of the selected genes in members of FLW groups.

Supplementary Figure 6 Dependence modeling between tensile strength, flexibility, and technical length (used as dependent variable) and relative expression level (ΔCq-value) of Intrusive-upregulated and TCW-upregulated genes (used as independent variables) using LASSO method.

Supplementary Figure 7 Linear regression of flax phenotypic traits on relative expression level (ΔCq-value).

Supplementary Table 1 The primers used for qPCR in this study and gene sequences according to Phytozome DB.

Supplementary Table 2 ΔCq values of each studied gene in all analyzed flax samples.

Supplementary Table 3 Single-nucleotide polymorphisms (SNPs) found in the studied genes (TCW-upregulated).

Supplementary Table 4 Mapping of analyzed genes (scaffolds, chromosomes, and position on the physical and genetic maps).
