# Supplementary material for: The Toolbox for Fiber Flax Breeding: A Pipeline From Gene Expression to Fiber Quality
Source: Front Genet. 2020 Nov 12;11:589881. doi: 10.3389/fgene.2020.589881 (PMC7690631; doi:10.3389/fgene.2020.589881)
Supplement: Supplementary Figure 1 — Validation of qPCR data for 32 genes (TCW-upregulated) using RNA-Seq data. [file Data_Sheet_1.PDF]

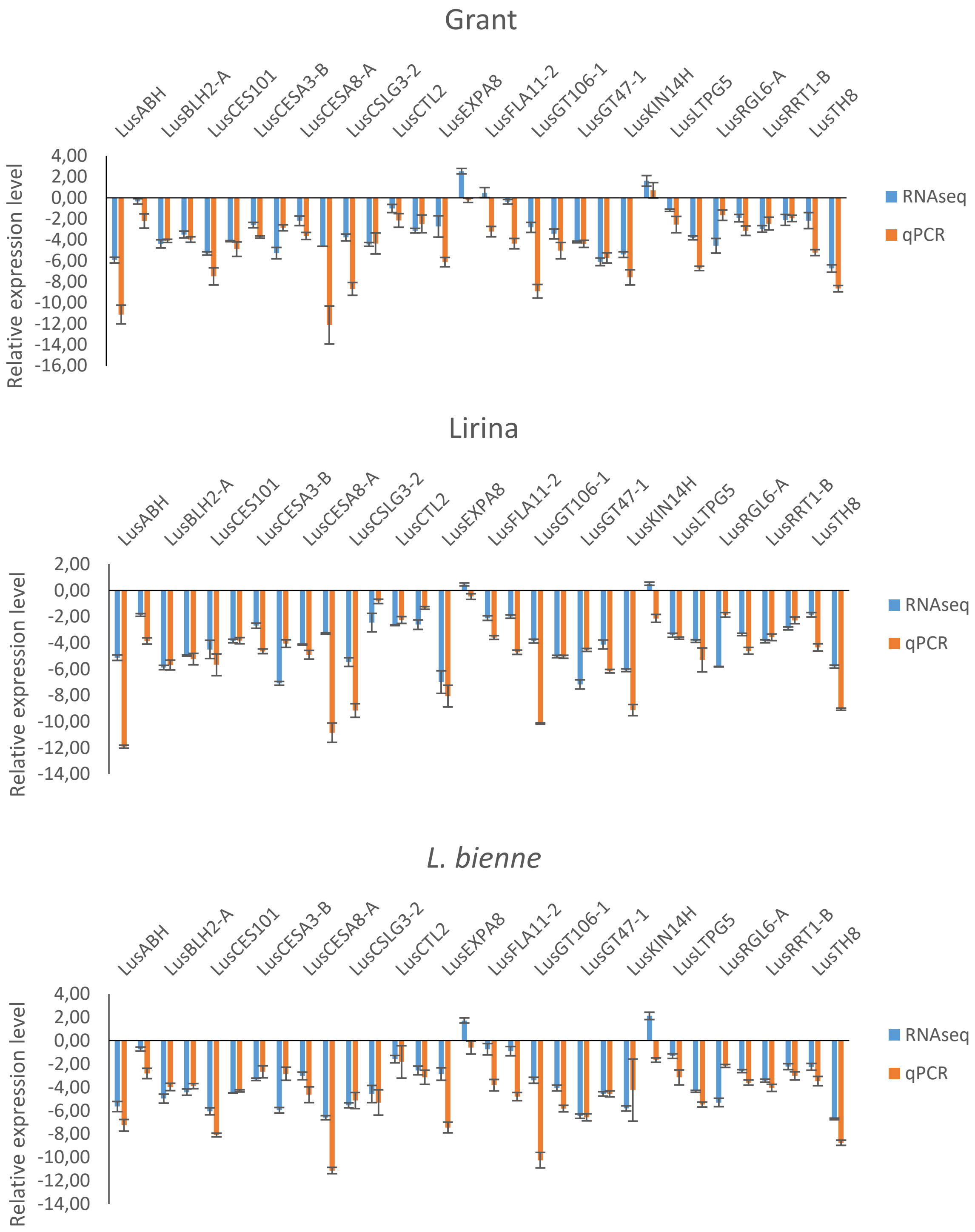

Figure S1. Validation of qPCR data for 32 genes (TCW-upregulated) using RNA-Seq data. The relative expression levels (log2 – scale) obtained by qPCR (orange columns) and RNAseq (blue columns) for TCW up-regulated genes.
