# Supplementary material for: The Toolbox for Fiber Flax Breeding: A Pipeline From Gene Expression to Fiber Quality
Source: Front Genet. 2020 Nov 12;11:589881. doi: 10.3389/fgene.2020.589881 (PMC7690631; doi:10.3389/fgene.2020.589881)
Supplement: Supplementary Figure 2 — The distribution of elementary fiber length (A) and average length of individual fibers (B) in representatives of FLW groups. [file Data_Sheet_2.pdf]

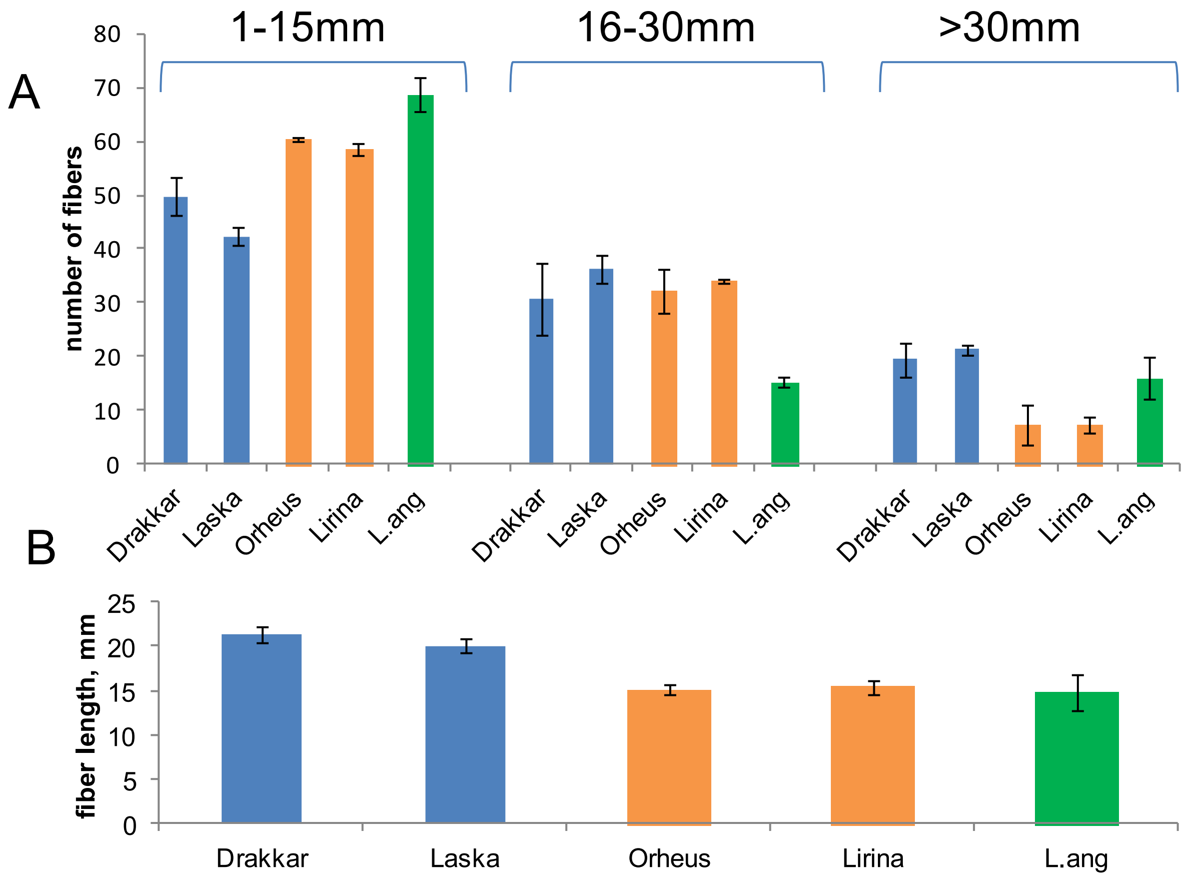

Figure S2. The distribution of elementary fiber length (A) and average length of individual fibers (B) in representatives of FLW groups. Blue, yellow, and green backgrounds correspond to fiber flax cultivars, linseed flax cultivars, and wild type, respectively. To determine the length of the fibers, 8 cm segments of dry mature flax stems were taken (located at the similar stem heights as the stem parts that were earlier used for intrusive-specific gene expression analysis). Stem segments were treated with 1% pectinase solution (Pectinase from *Aspergillus niger*, Sigma, Germany) overnight at room temperature. Using dissecting needles, a fiber bundle was isolated from the macerated segments and completely separated into individual fibers. Cell integrity was checked under an Axio light microscope (Zeiss, Germany) and fiber length was measured. On average, 130 individual fibers sampling from 2 plants were measured for each cultivar (subspecies). Data performed for flax harvested in 2018.
