# Supplementary material for: The Toolbox for Fiber Flax Breeding: A Pipeline From Gene Expression to Fiber Quality
Source: Front Genet. 2020 Nov 12;11:589881. doi: 10.3389/fgene.2020.589881 (PMC7690631; doi:10.3389/fgene.2020.589881)
Supplement: Supplementary Figure 3 — The relative expression levels (qPCR) of each studied gene (in alphabetical order) in all analyzed flax genotypes. [file Data_Sheet_3.pdf]

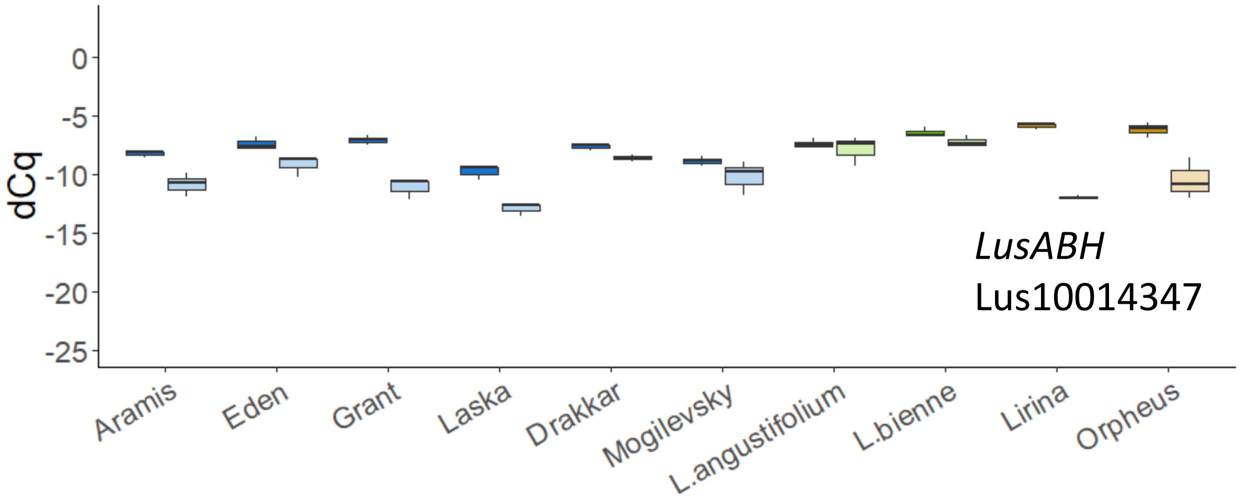
0
-5
-10
-15
-20
-25
dCq
LusABH
Lus10014347
Aramis
Eden
Grant
Laska
Drakkar
Mogilevsky
L.angustifolium
L.bienne
Lirina
Orpheus

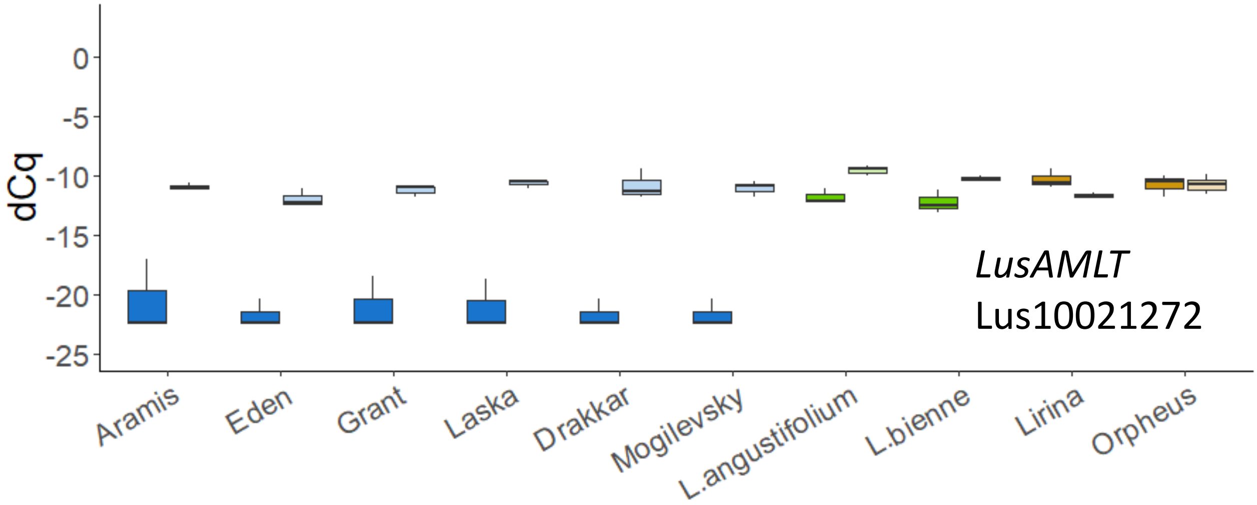
0
-5
-10
-15
-20
-25
dCq
LusAMLT
Lus10021272
Aramis
Eden
Grant
Laska
Drakkar
Mogilevsky
L.angustifolium
L.bienne
Lirina
Orpheus

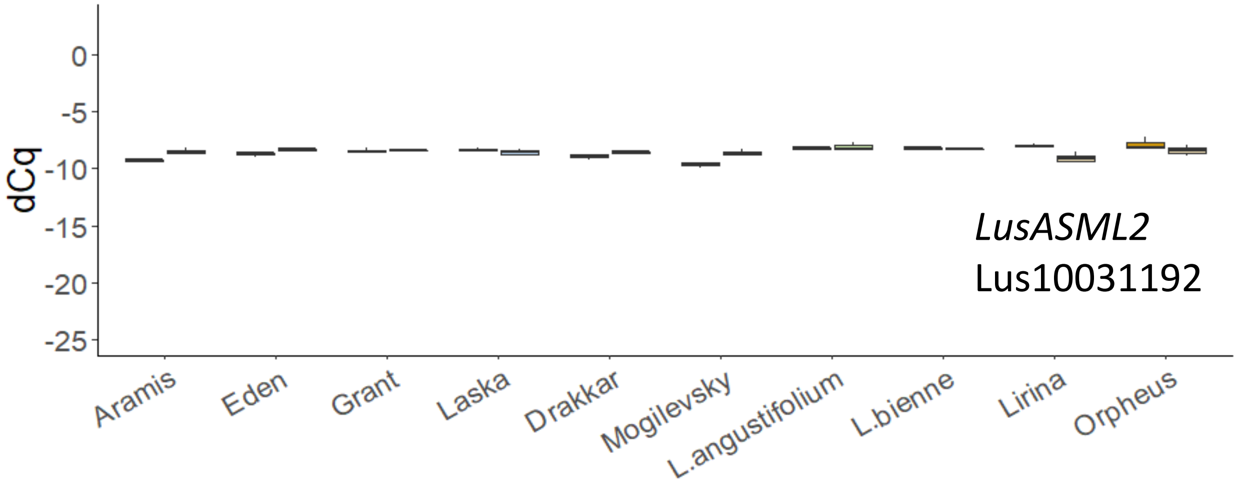
0
-5
-10
-15
-20
-25
dCq
LusASML2
Lus10031192
Aramis
Eden
Grant
Laska
Drakkar
Mogilevsky
L.angustifolium
L.bienne
Lirina
Orpheus

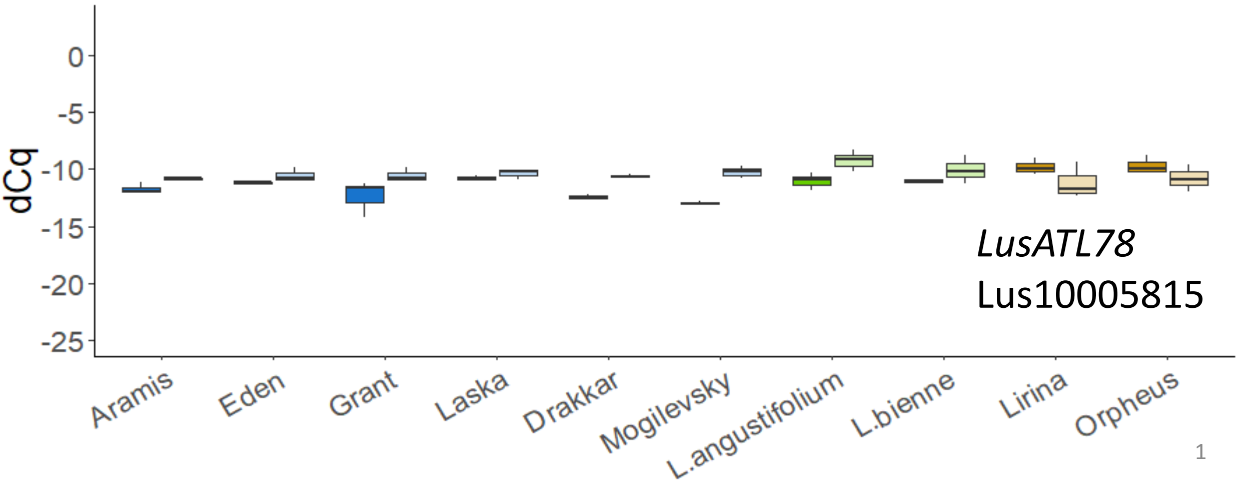
0
-5
-10
-15
-20
-25
dCq
LusATL78
Lus10005815
Aramis
Eden
Grant
Laska
Drakkar
Mogilevsky
L.angustifolium
L.bienne
Lirina
Orpheus

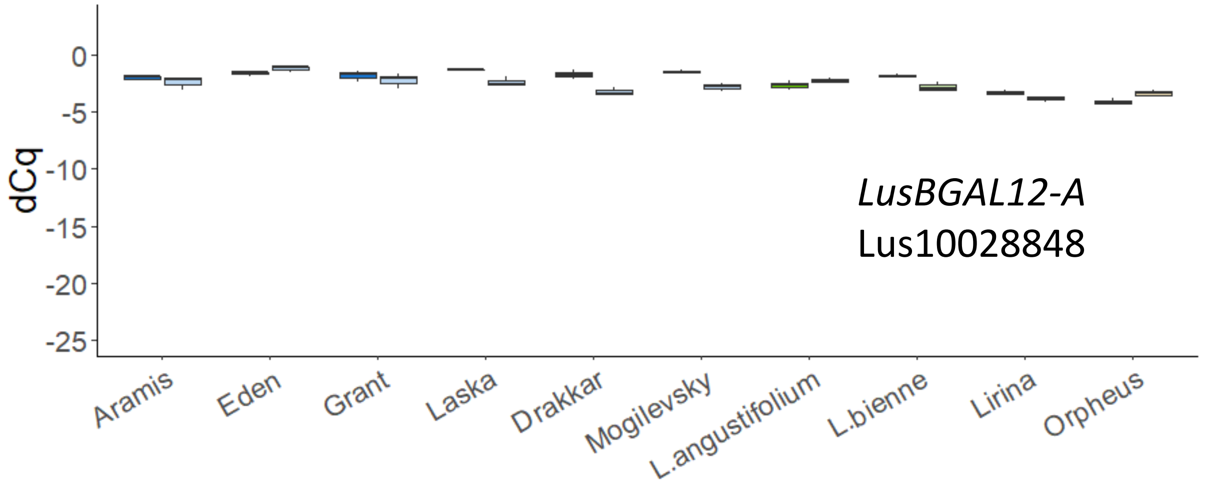
0
-5
-10
-15
-20
-25
dCq
LusBGAL12-A
Lus10028848
Aramis
Eden
Grant
Laska
Drakkar
Mogilevsky
L.angustifolium
L.bienne
Lirina
Orpheus

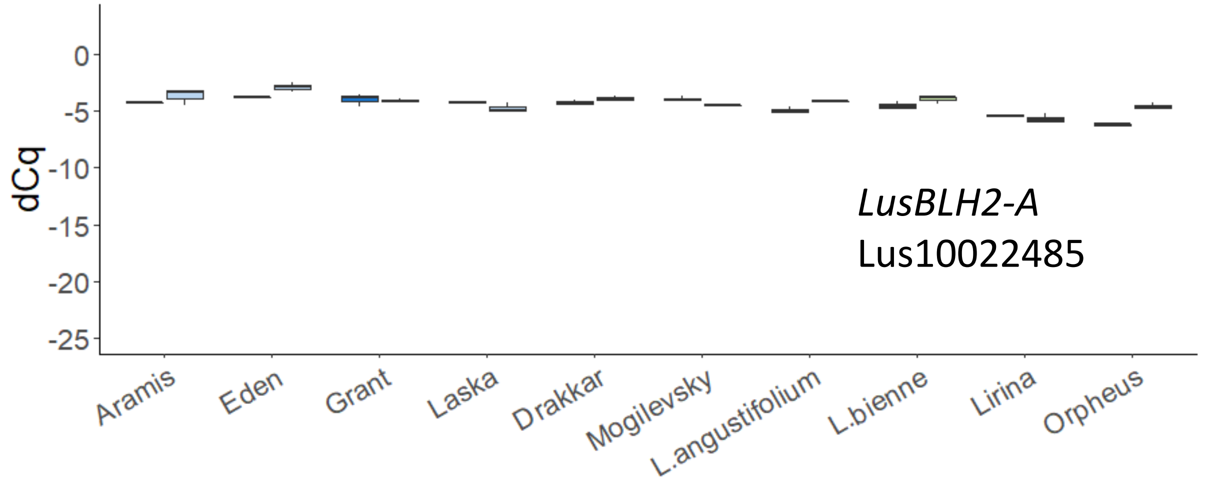
0
-5
-10
-15
-20
-25
dCq
LusBLH2-A
Lus10022485
Aramis
Eden
Grant
Laska
Drakkar
Mogilevsky
L.angustifolium
L.bienne
Lirina
Orpheus

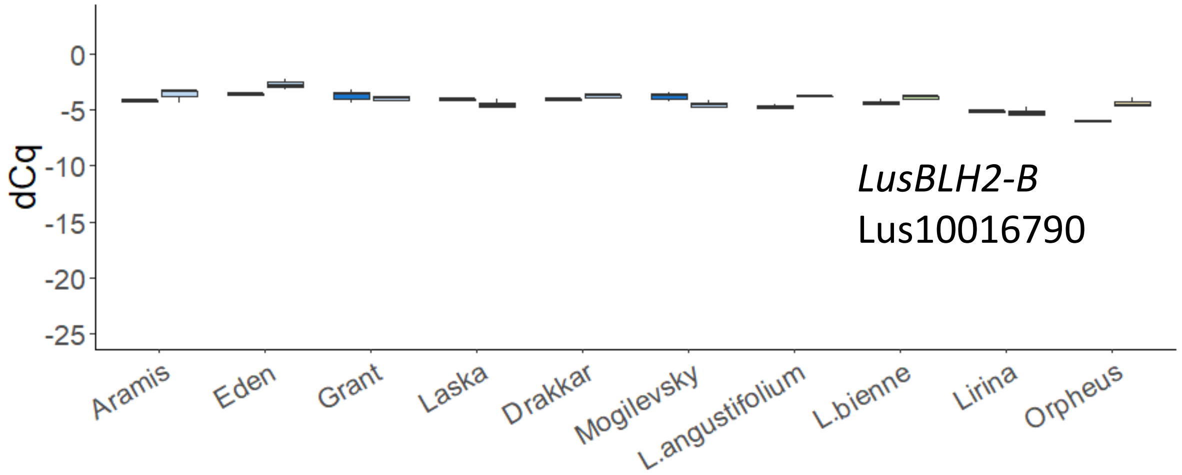
0
-5
-10
-15
-20
-25
dCq
LusBLH2-B
Lus10016790
Aramis
Eden
Grant
Laska
Drakkar
Mogilevsky
L.angustifolium
L.bienne
Lirina
Orpheus

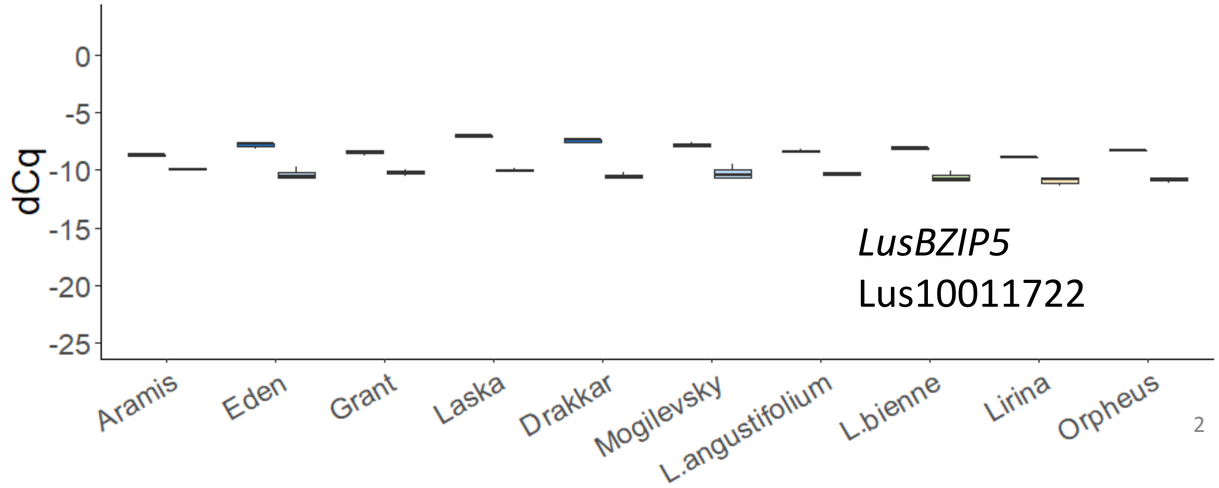
0
-5
-10
-15
-20
-25
dCq
LusBZIP5
Lus10011722
Aramis
Eden
Grant
Laska
Drakkar
Mogilevsky
L.angustifolium
L.bienne
Lirina
Orpheus

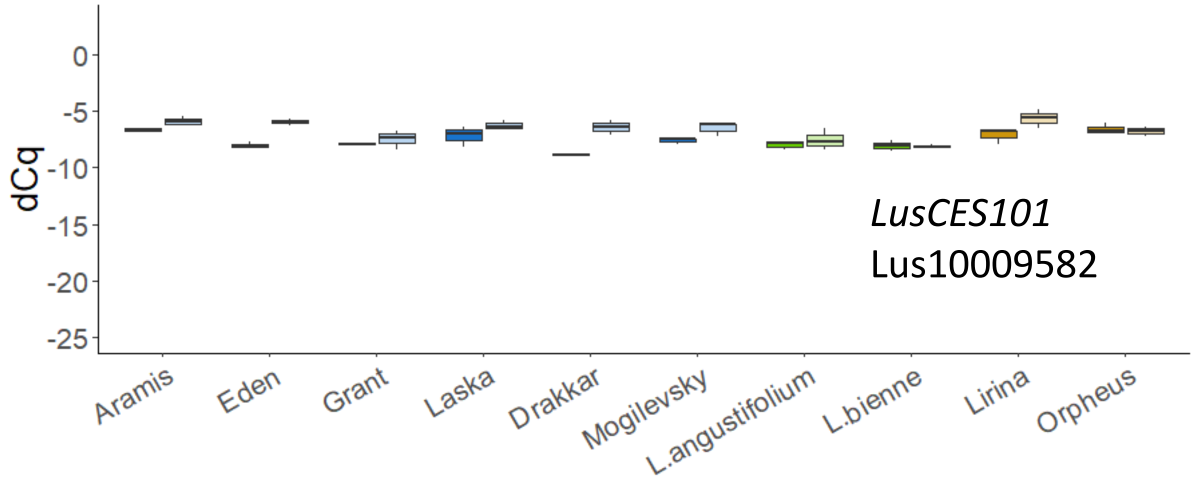
dCq
0
-5
-10
-15
-20
-25
LusCES101
Lus10009582
Aramis
Eden
Grant
Laska
Drakkar
Mogilevsky
L.angustifolium
L.bienne
Lirina
Orpheus

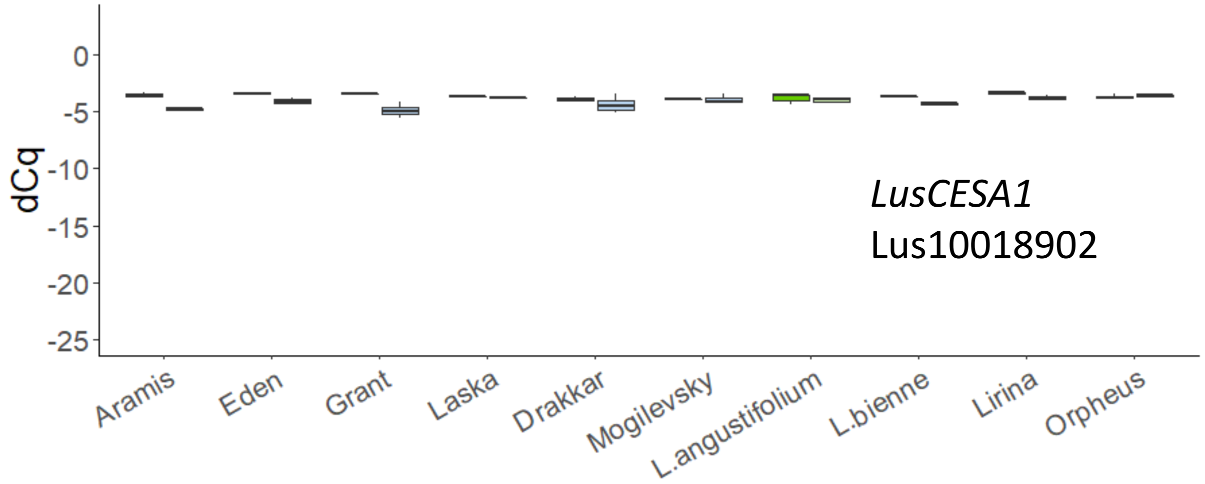
dCq
0
-5
-10
-15
-20
-25
LusCESA1
Lus10018902
Aramis
Eden
Grant
Laska
Drakkar
Mogilevsky
L.angustifolium
L.bienne
Lirina
Orpheus

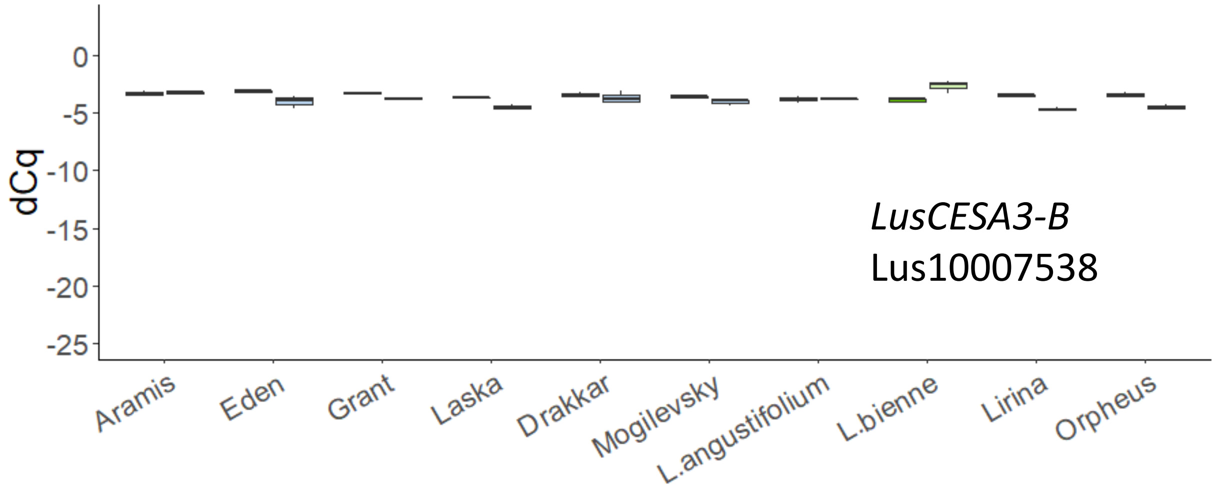
dCq
0
-5
-10
-15
-20
-25
LusCESA3-B
Lus10007538
Aramis
Eden
Grant
Laska
Drakkar
Mogilevsky
L.angustifolium
L.bienne
Lirina
Orpheus

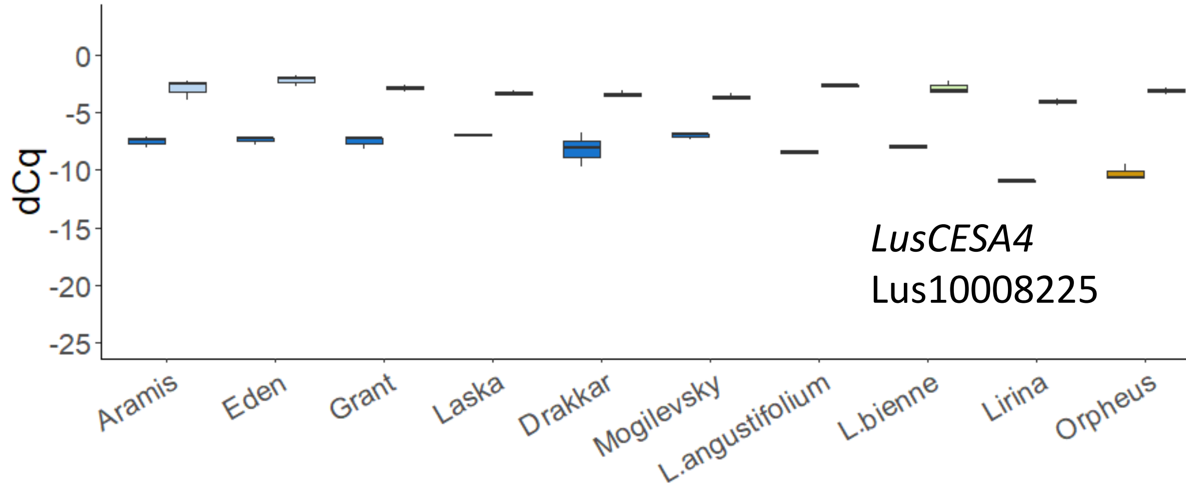
dCq
0
-5
-10
-15
-20
-25
LusCESA4
Lus10008225
Aramis
Eden
Grant
Laska
Drakkar
Mogilevsky
L.angustifolium
L.bienne
Lirina
Orpheus

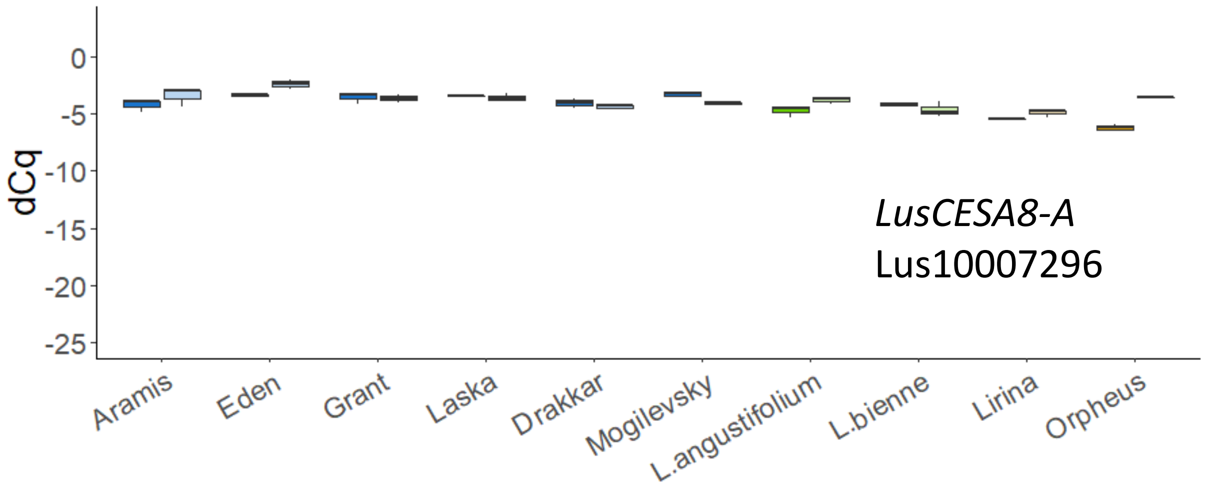
dCq
0
-5
-10
-15
-20
-25
LusCESA8-A
Lus10007296
Aramis
Eden
Grant
Laska
Drakkar
Mogilevsky
L.angustifolium
L.bienne
Lirina
Orpheus

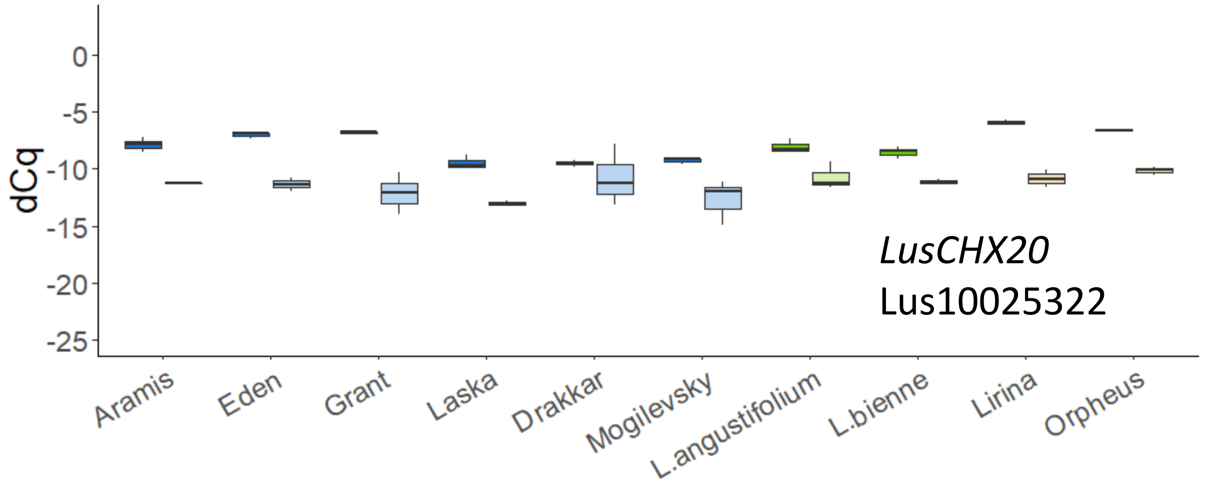
dCq
0
-5
-10
-15
-20
-25
LusCHX20
Lus10025322
Aramis
Eden
Grant
Laska
Drakkar
Mogilevsky
L.angustifolium
L.bienne
Lirina
Orpheus

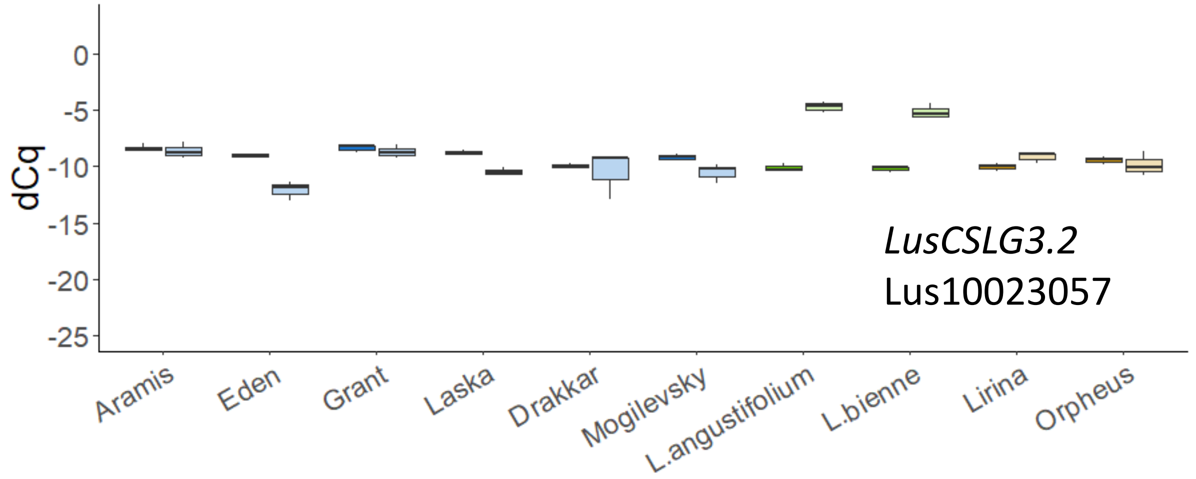
dCq
0
-5
-10
-15
-20
-25
LusCSLG3.2
Lus10023057
Aramis
Eden
Grant
Laska
Drakkar
Mogilevsky
L.angustifolium
L.bienne
Lirina
Orpheus

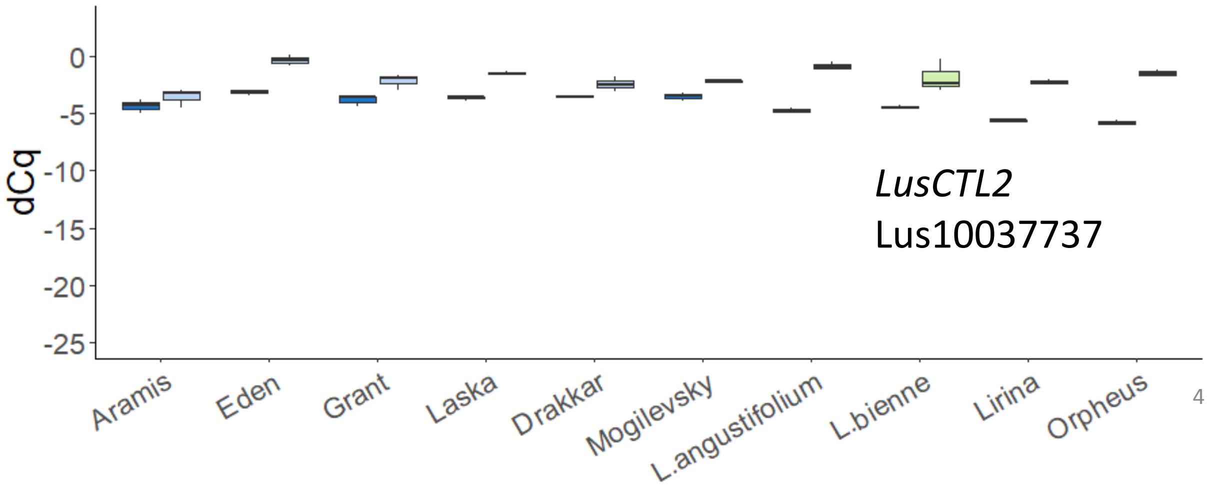
dCq
0
-5
-10
-15
-20
-25
LusCTL2
Lus10037737
Aramis
Eden
Grant
Laska
Drakkar
Mogilevsky
L.angustifolium
L.bienne
Lirina
Orpheus

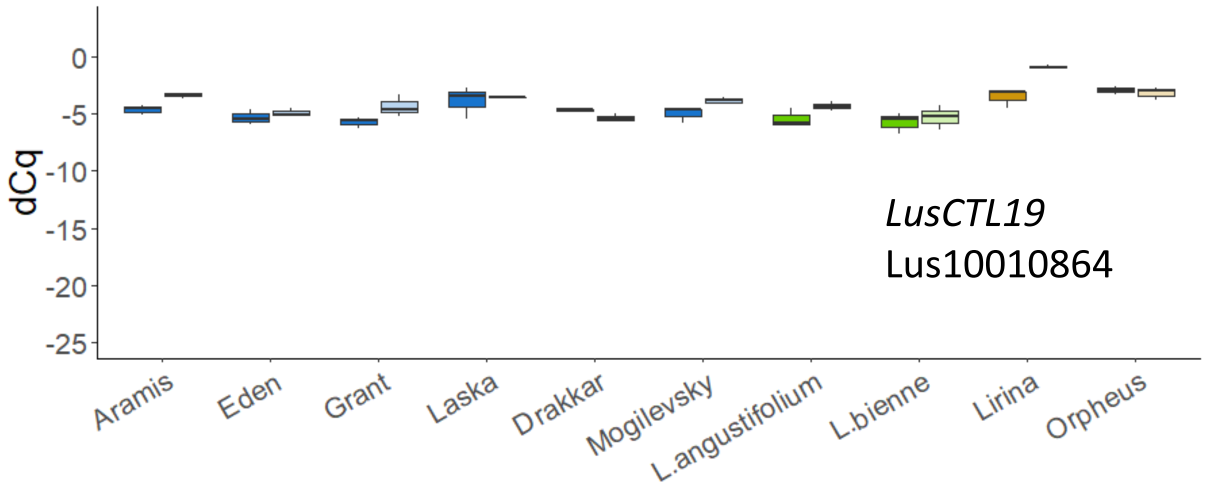
0
-5
-10
-15
-20
-25
dCq
LusCTL19
Lus10010864
Aramis
Eden
Grant
Laska
Drakkar
Mogilevsky
L.angustifolium
L.bienne
Lirina
Orpheus

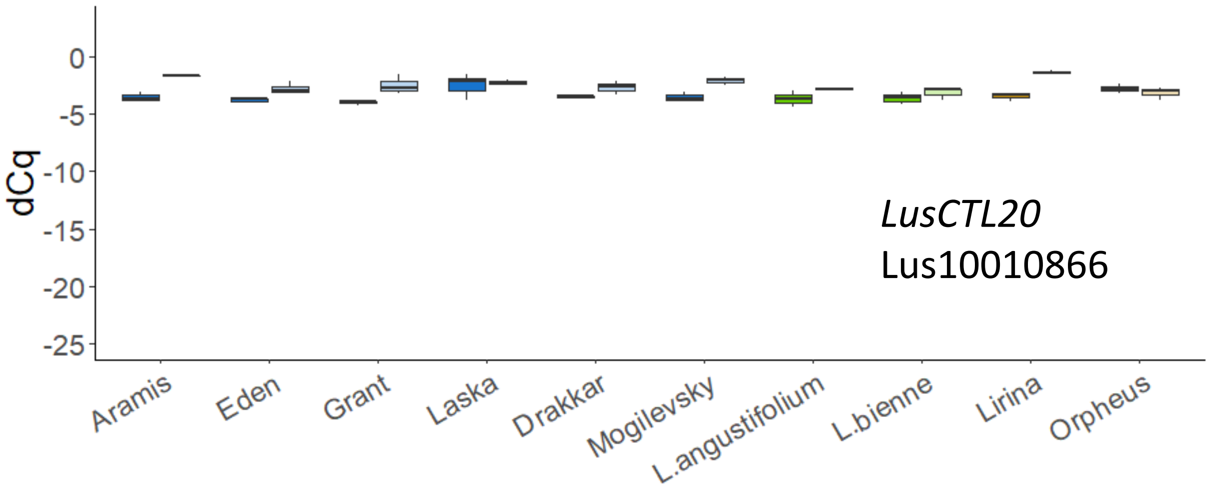
0
-5
-10
-15
-20
-25
dCq
LusCTL20
Lus10010866
Aramis
Eden
Grant
Laska
Drakkar
Mogilevsky
L.angustifolium
L.bienne
Lirina
Orpheus

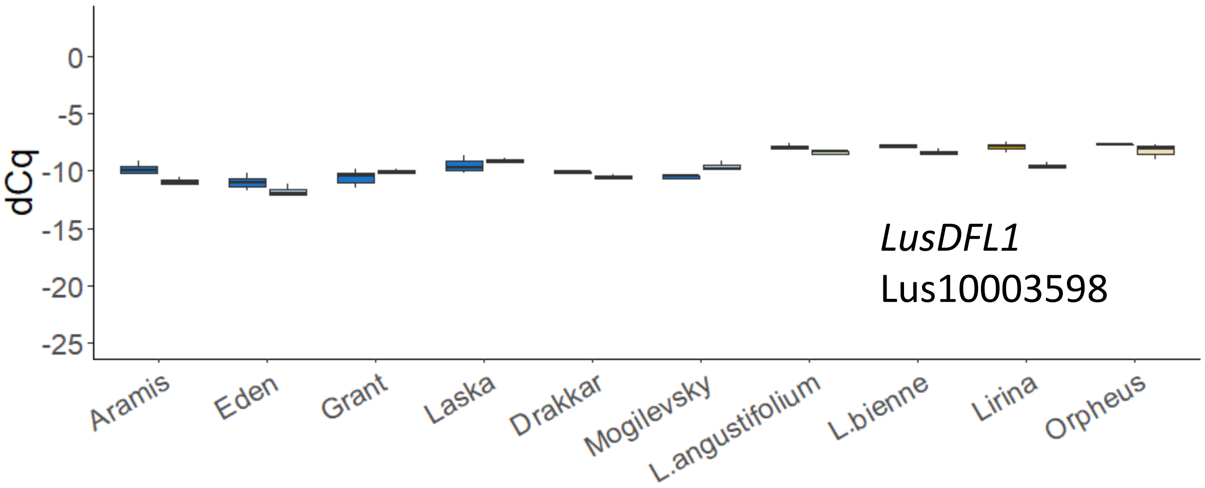
0
-5
-10
-15
-20
-25
dCq
LusDFL1
Lus10003598
Aramis
Eden
Grant
Laska
Drakkar
Mogilevsky
L.angustifolium
L.bienne
Lirina
Orpheus

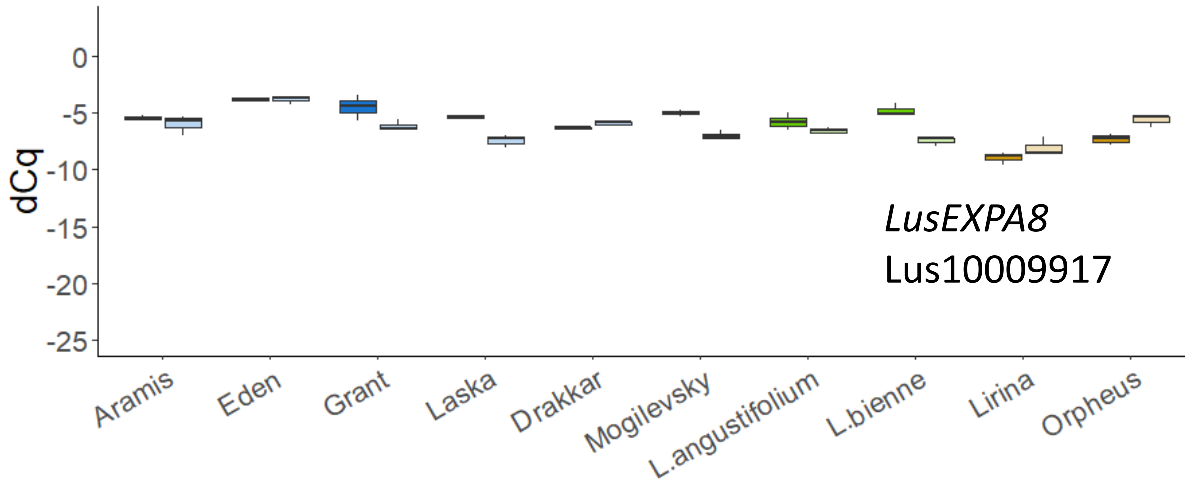
0
-5
-10
-15
-20
-25
dCq
LusEXPA8
Lus10009917
Aramis
Eden
Grant
Laska
Drakkar
Mogilevsky
L.angustifolium
L.bienne
Lirina
Orpheus

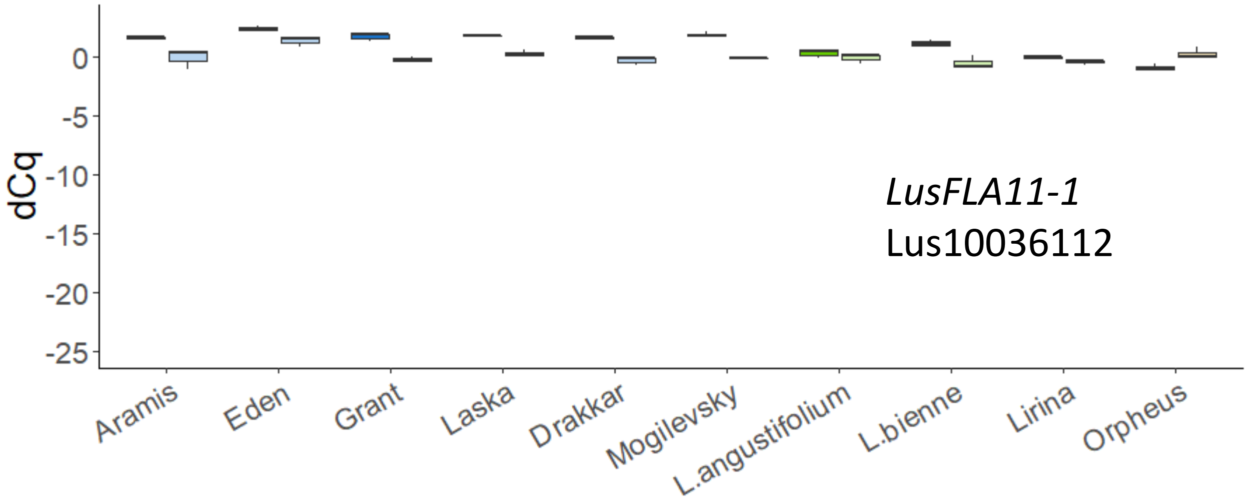

0
-5
-10
-15
-20
-25
dCq
LusFLA11-1
Lus10036112
Aramis
Eden
Grant
Laska
Drakkar
Mogilevsky
L.angustifolium
L.bienne
Lirina
Orpheus

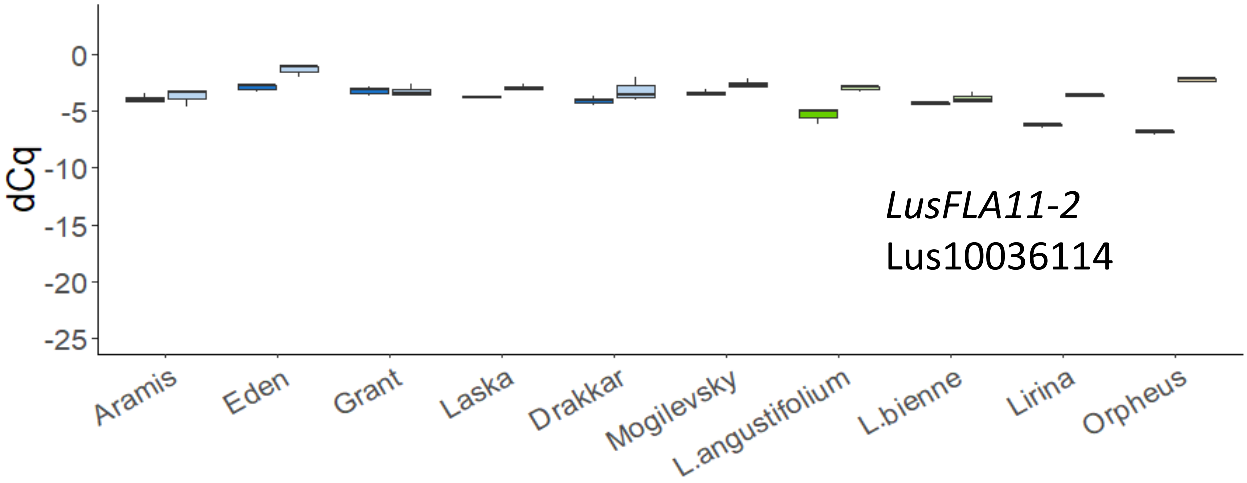

0
-5
-10
-15
-20
-25
dCq
LusFLA11-2
Lus10036114
Aramis
Eden
Grant
Laska
Drakkar
Mogilevsky
L.angustifolium
L.bienne
Lirina
Orpheus

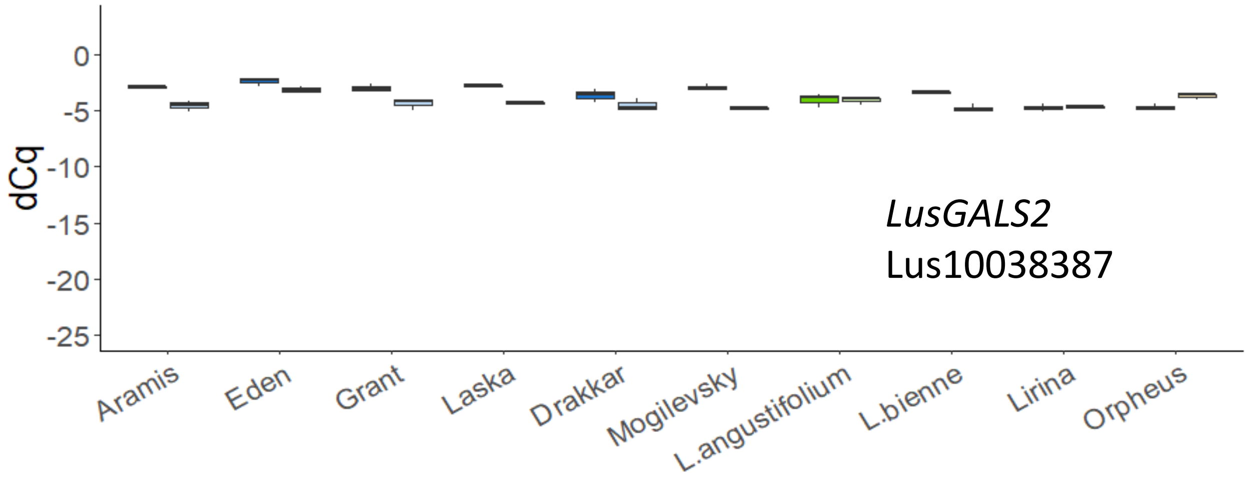

0
-5
-10
-15
-20
-25
dCq
LusGALS2
Lus10038387
Aramis
Eden
Grant
Laska
Drakkar
Mogilevsky
L.angustifolium
L.bienne
Lirina
Orpheus

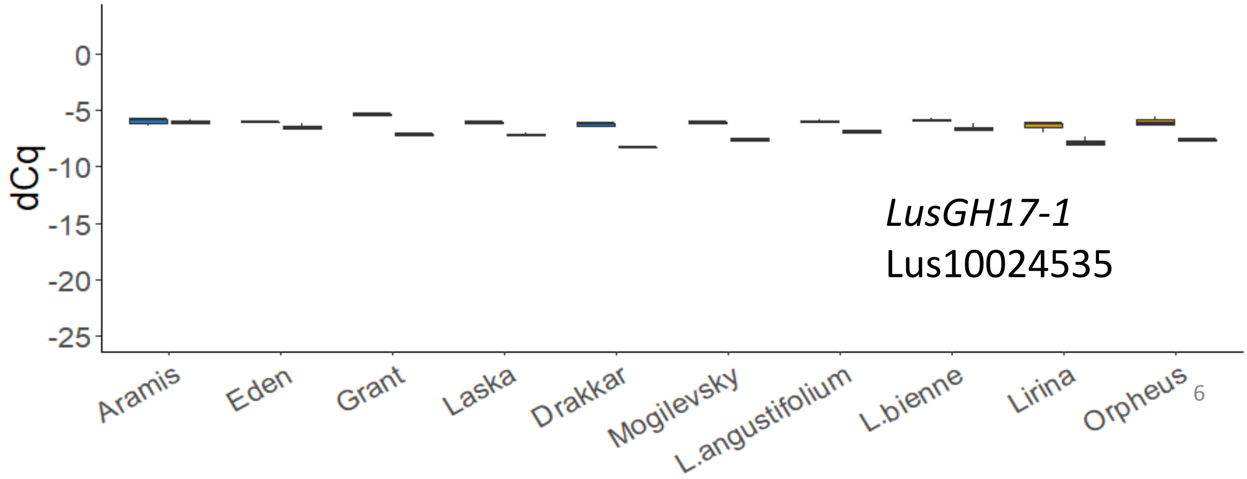

0
-5
-10
-15
-20
-25
dCq
LusGH17-1
Lus10024535
Aramis
Eden
Grant
Laska
Drakkar
Mogilevsky
L.angustifolium
L.bienne
Lirina
Orpheus

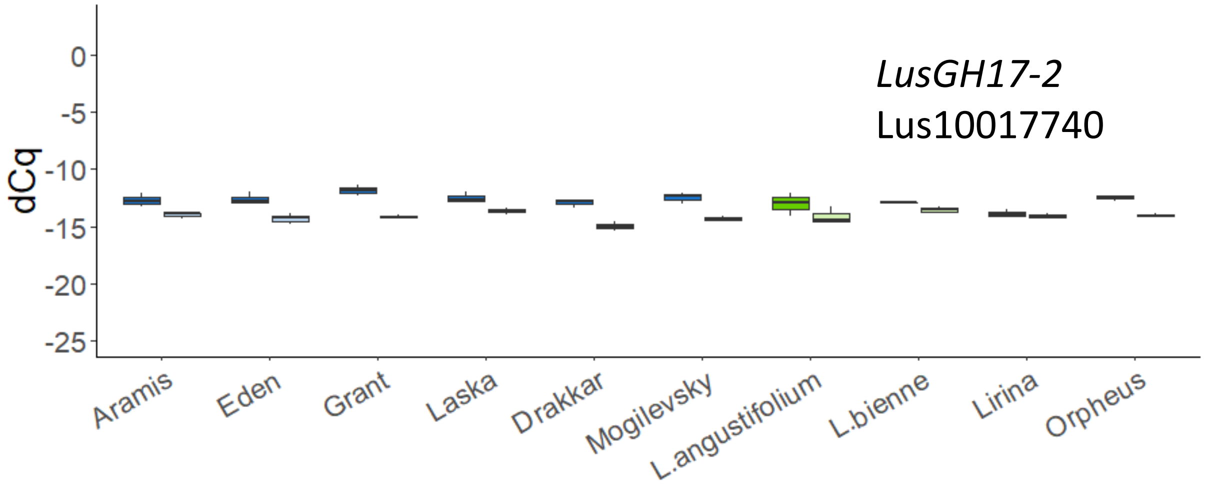
LusGH17-2
Lus10017740
dCq
0
-5
-10
-15
-20
-25
Aramis
Eden
Grant
Laska
Drakkar
Mogilevsky
L.angustifolium
L.bienne
Lirina
Orpheus

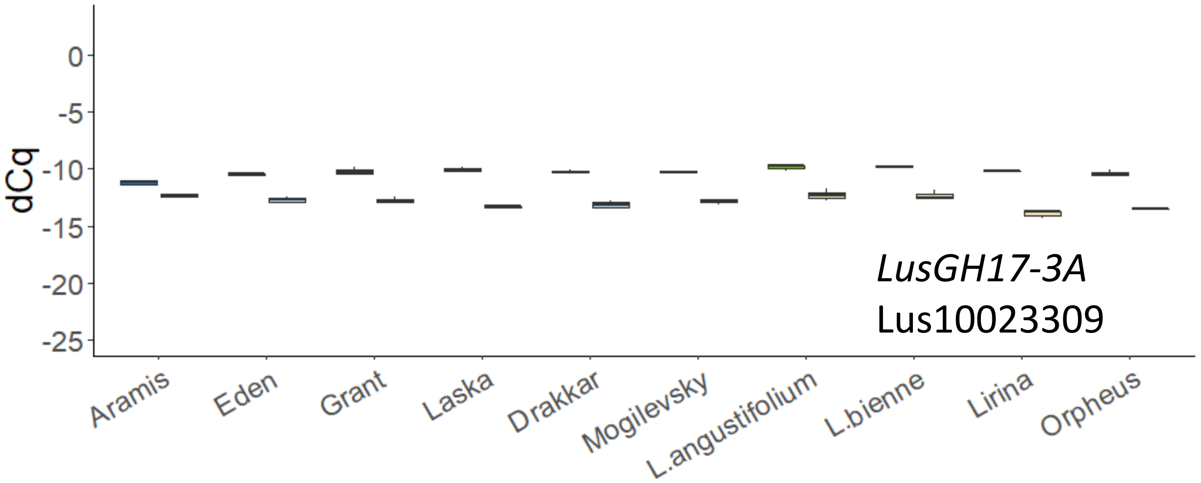
LusGH17-3A
Lus10023309
dCq
0
-5
-10
-15
-20
-25
Aramis
Eden
Grant
Laska
Drakkar
Mogilevsky
L.angustifolium
L.bienne
Lirina
Orpheus

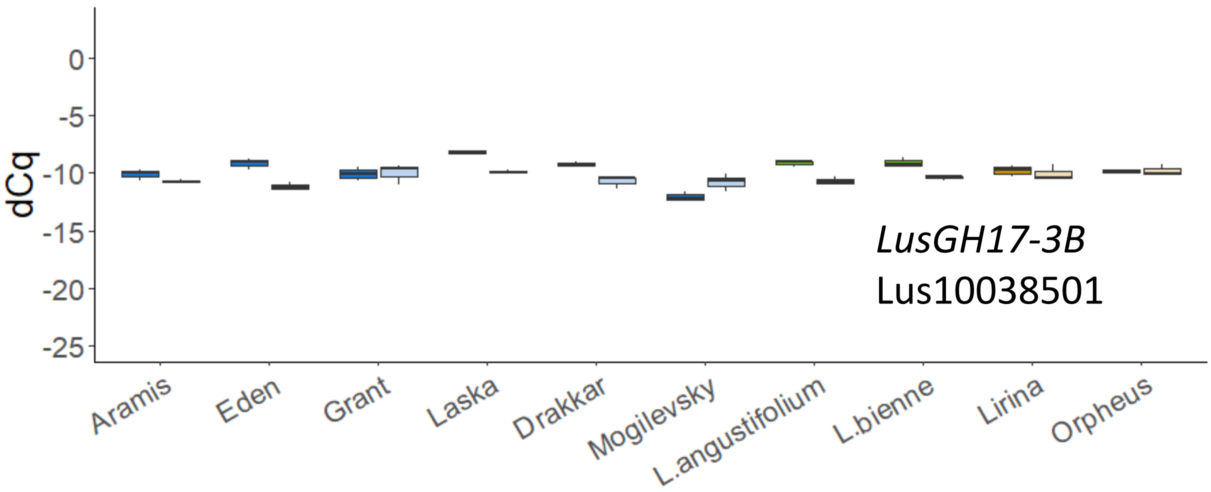
LusGH17-3B
Lus10038501
dCq
0
-5
-10
-15
-20
-25
Aramis
Eden
Grant
Laska
Drakkar
Mogilevsky
L.angustifolium
L.bienne
Lirina
Orpheus

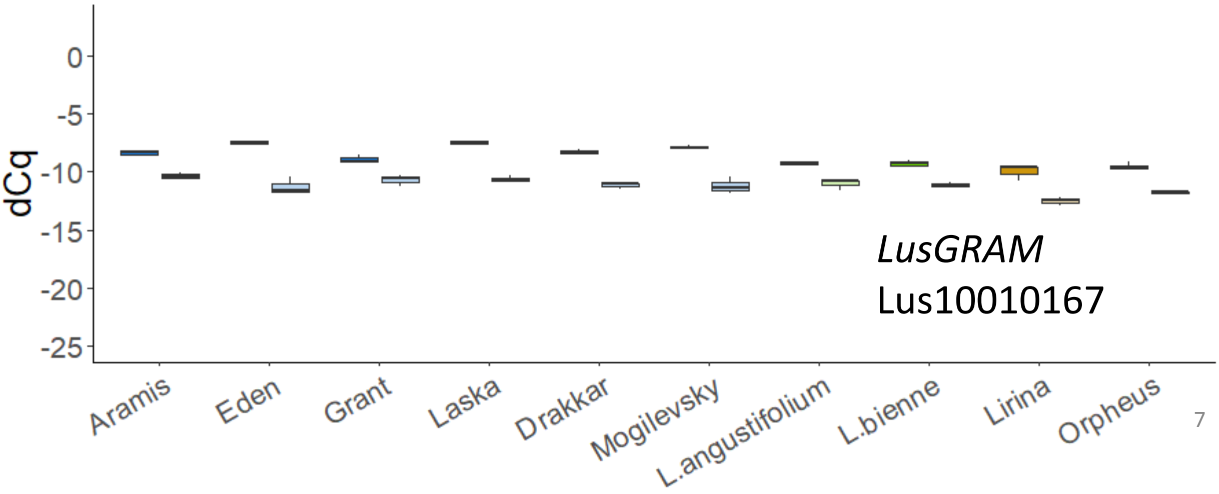
LusGRAM
Lus10010167
dCq
0
-5
-10
-15
-20
-25
Aramis
Eden
Grant
Laska
Drakkar
Mogilevsky
L.angustifolium
L.bienne
Lirina
Orpheus

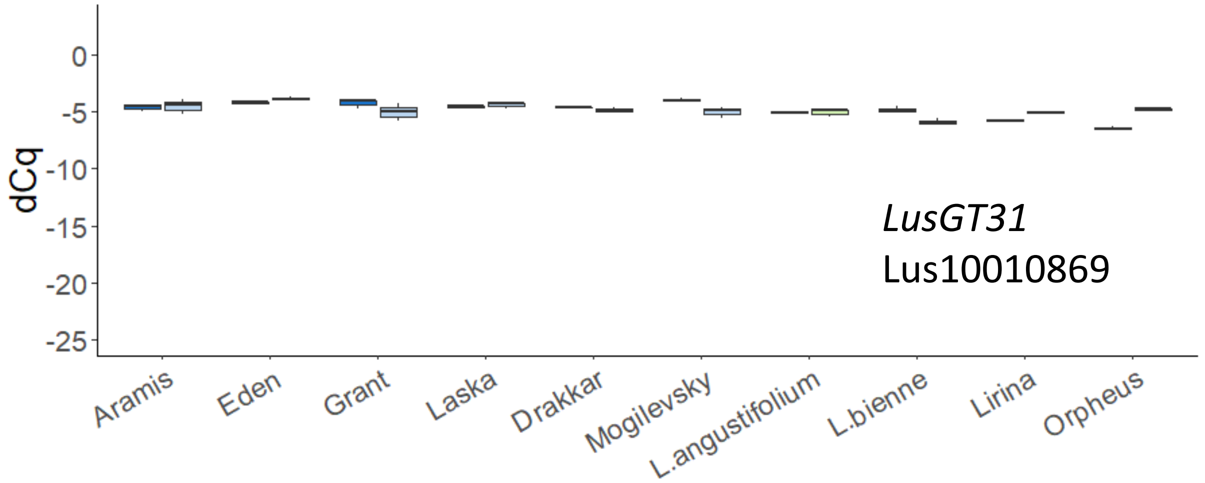
0
-5
-10
-15
-20
-25
dCq
LusGT31
Lus10010869
Aramis
Eden
Grant
Laska
Drakkar
Mogilevsky
L.angustifolium
L.bienne
Lirina
Orpheus

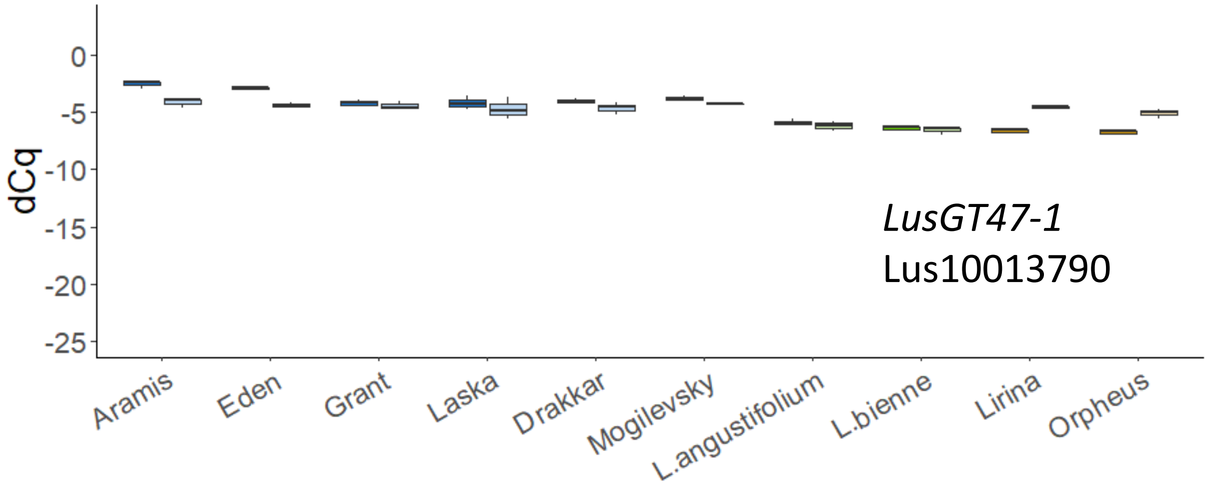
0
-5
-10
-15
-20
-25
dCq
LusGT47-1
Lus10013790
Aramis
Eden
Grant
Laska
Drakkar
Mogilevsky
L.angustifolium
L.bienne
Lirina
Orpheus

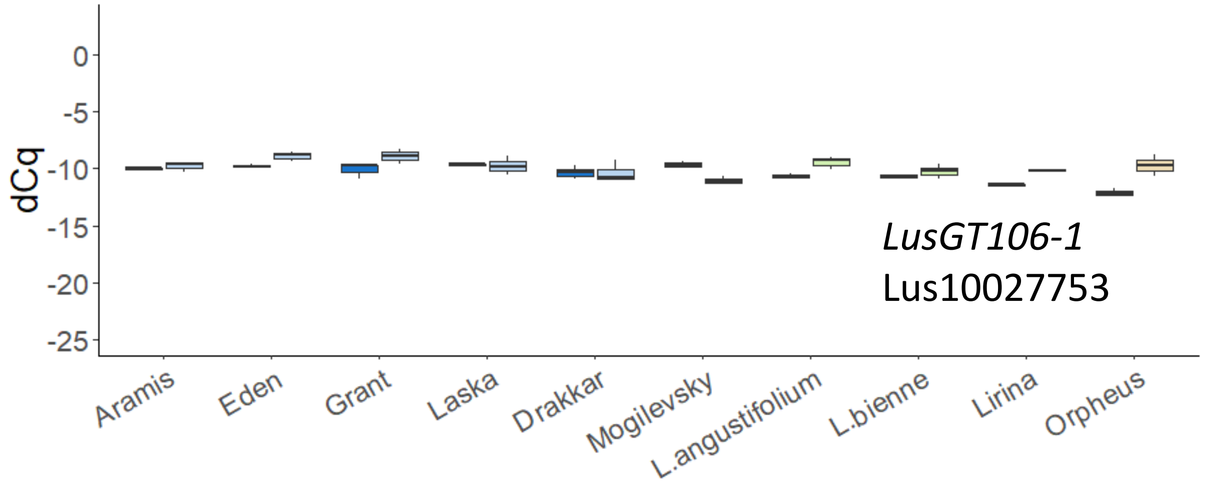
0
-5
-10
-15
-20
-25
dCq
LusGT106-1
Lus10027753
Aramis
Eden
Grant
Laska
Drakkar
Mogilevsky
L.angustifolium
L.bienne
Lirina
Orpheus

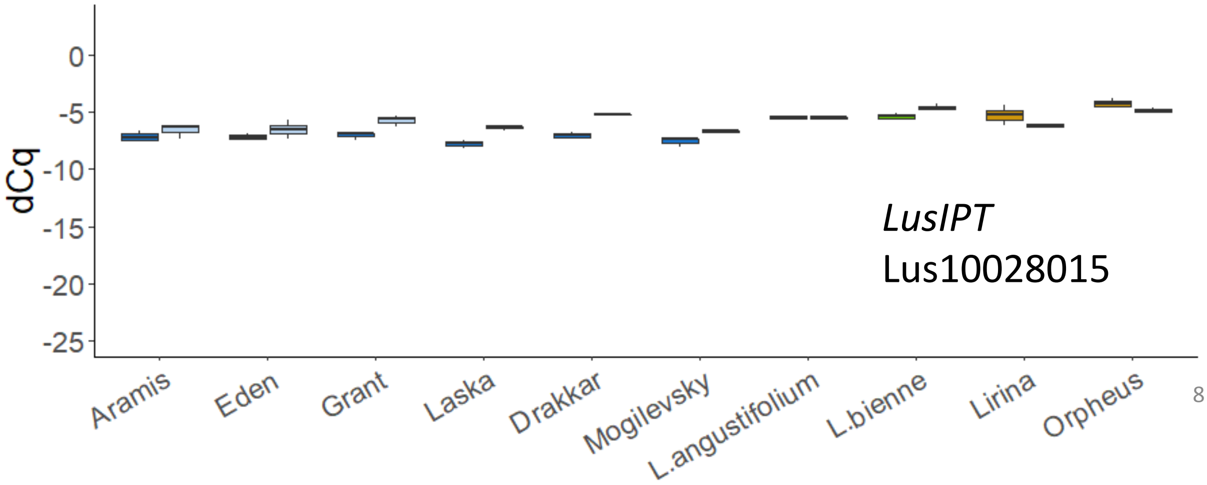
0
-5
-10
-15
-20
-25
dCq
LusIPT
Lus10028015
Aramis
Eden
Grant
Laska
Drakkar
Mogilevsky
L.angustifolium
L.bienne
Lirina
Orpheus

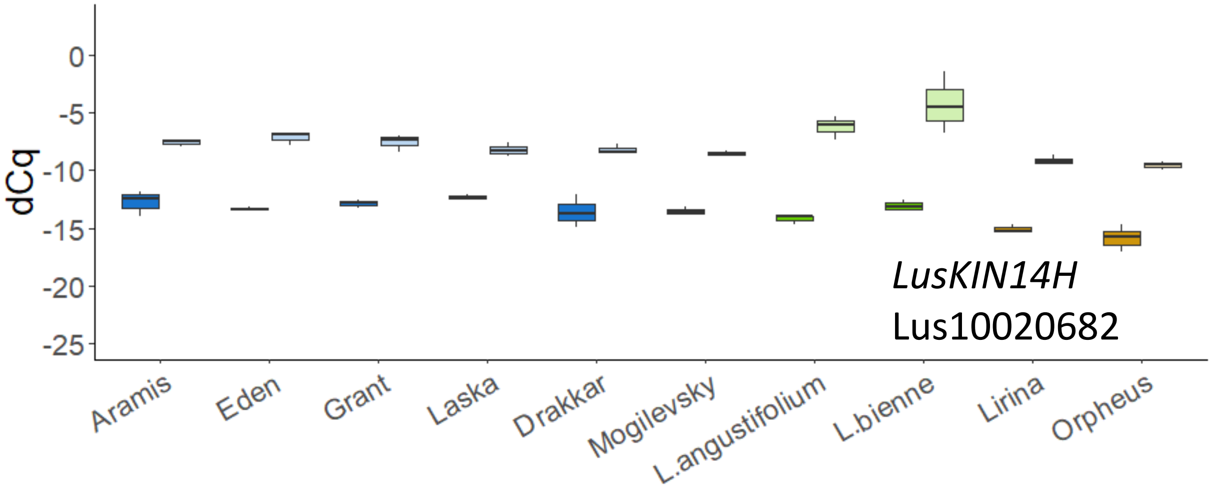
dCq
0
-5
-10
-15
-20
-25
Aramis
Eden
Grant
Laska
Drakkar
Mogilevsky
L.angustifolium
L.bienne
Lirina
Orpheus
LusKIN14H
Lus10020682

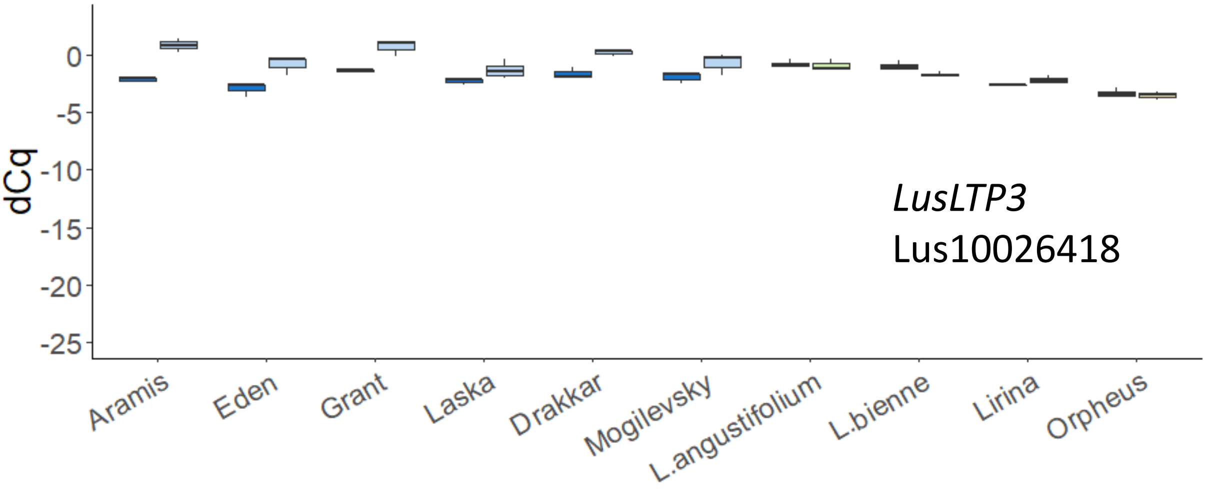
dCq
0
-5
-10
-15
-20
-25
Aramis
Eden
Grant
Laska
Drakkar
Mogilevsky
L.angustifolium
L.bienne
Lirina
Orpheus
LusLTP3
Lus10026418

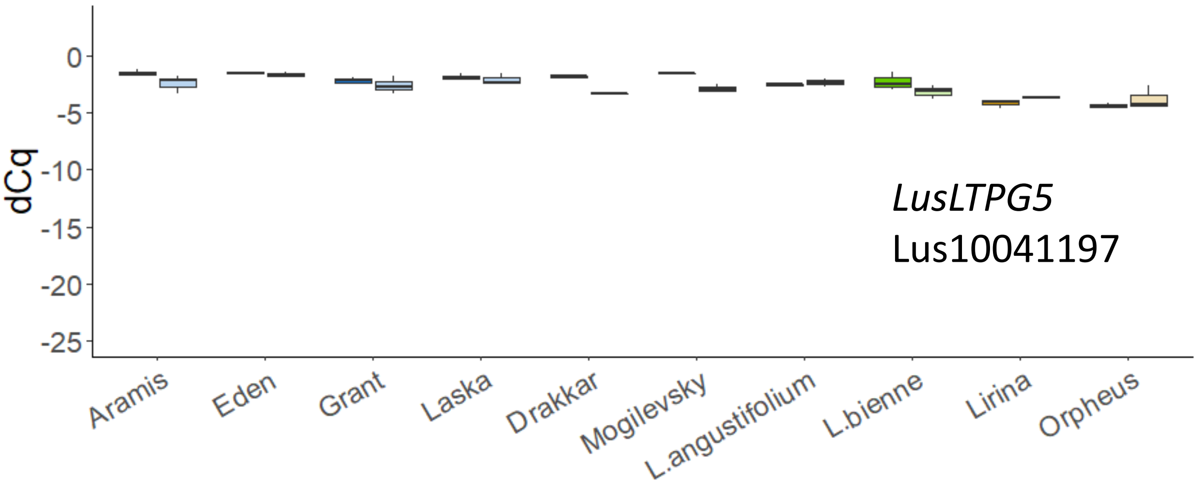
dCq
0
-5
-10
-15
-20
-25
Aramis
Eden
Grant
Laska
Drakkar
Mogilevsky
L.angustifolium
L.bienne
Lirina
Orpheus
LusLTPG5
Lus10041197

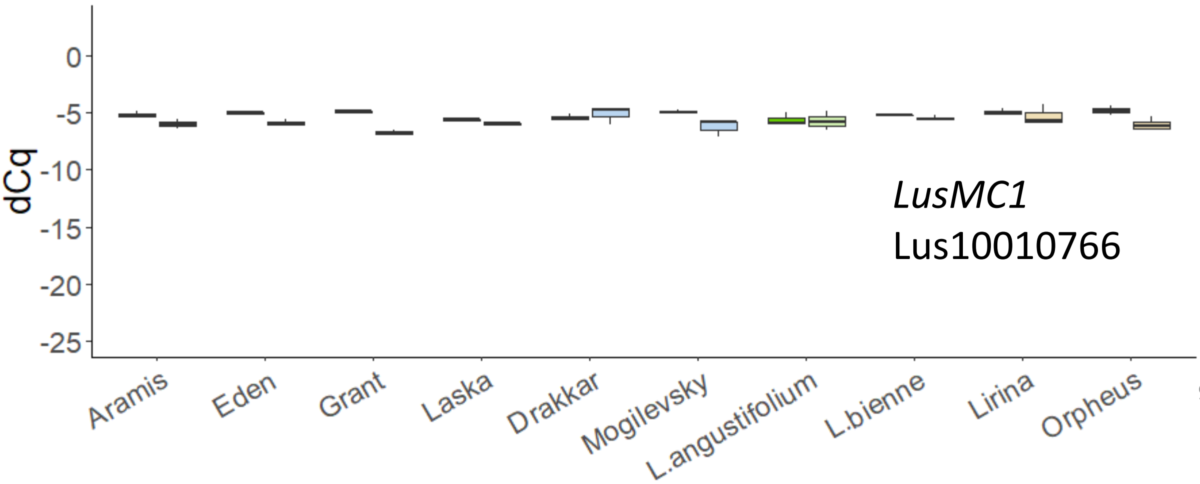
dCq
0
-5
-10
-15
-20
-25
Aramis
Eden
Grant
Laska
Drakkar
Mogilevsky
L.angustifolium
L.bienne
Lirina
Orpheus
LusMC1
Lus10010766

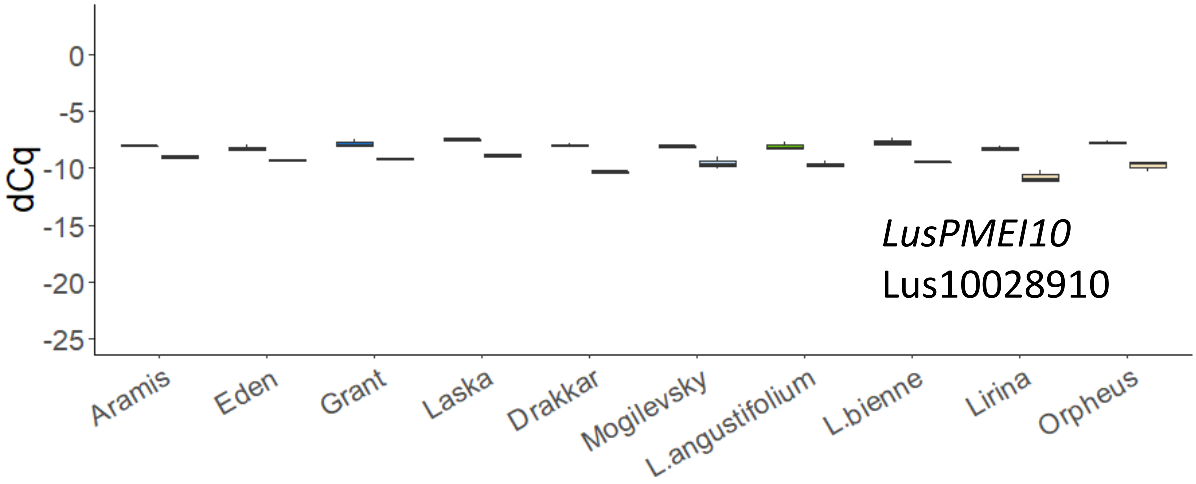
dCq
0
-5
-10
-15
-20
-25
LusPMEI10
Lus10028910
Aramis
Eden
Grant
Laska
Drakkar
Mogilevsky
L.angustifolium
L.bienne
Lirina
Orpheus

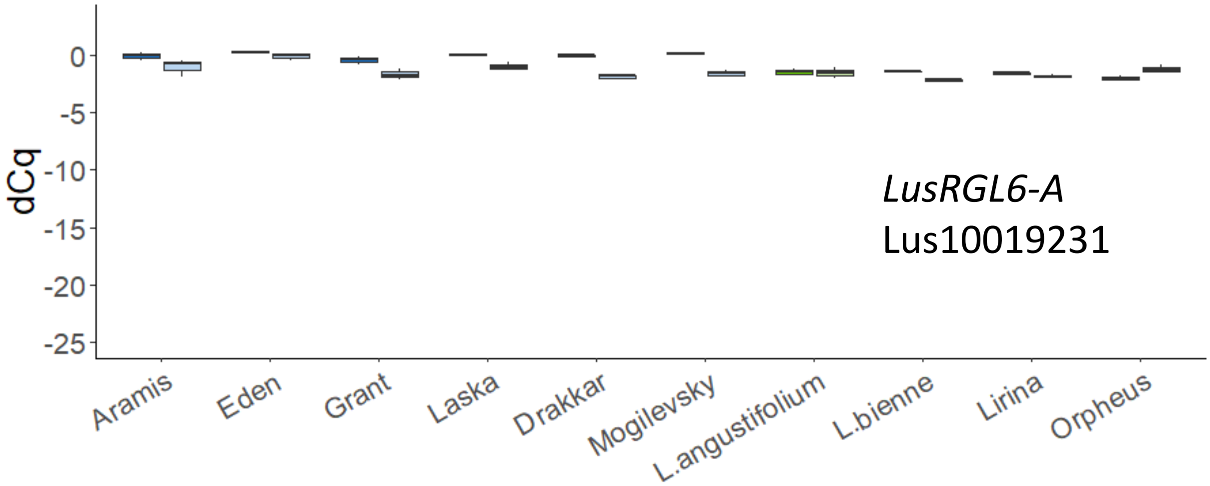
dCq
0
-5
-10
-15
-20
-25
LusRGL6-A
Lus10019231
Aramis
Eden
Grant
Laska
Drakkar
Mogilevsky
L.angustifolium
L.bienne
Lirina
Orpheus

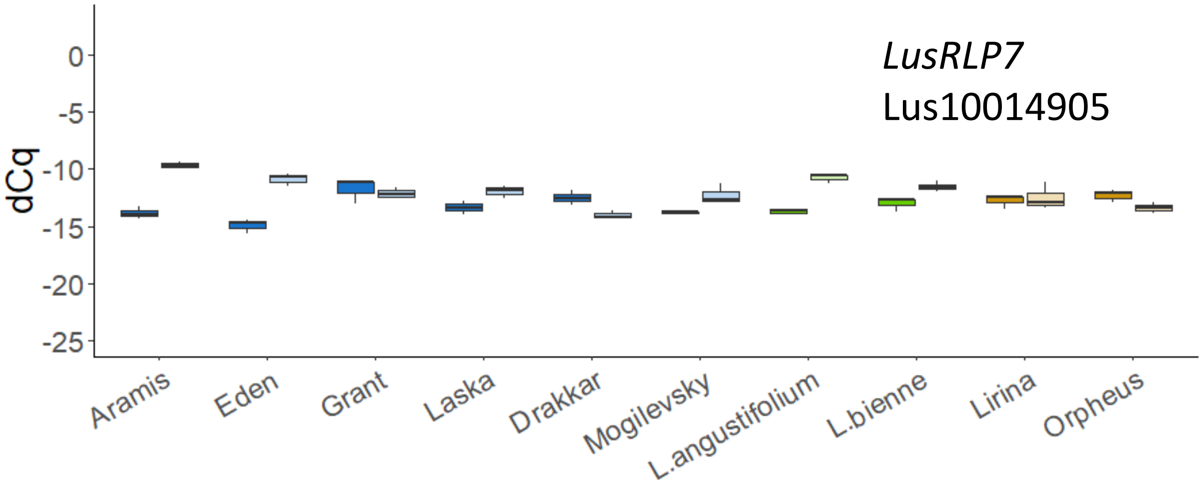
dCq
0
-5
-10
-15
-20
-25
LusRLP7
Lus10014905
Aramis
Eden
Grant
Laska
Drakkar
Mogilevsky
L.angustifolium
L.bienne
Lirina
Orpheus

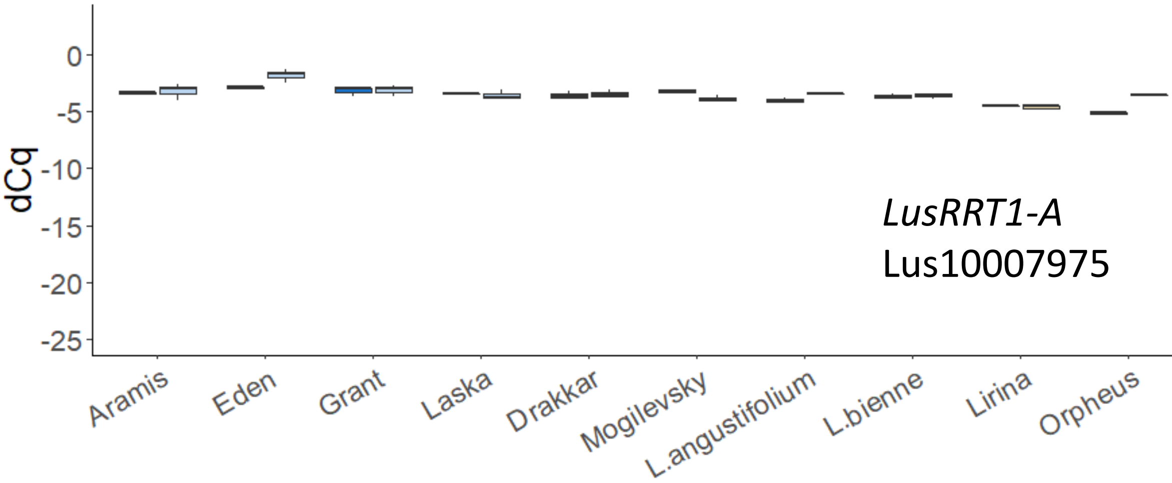
dCq
0
-5
-10
-15
-20
-25
LusRRT1-A
Lus10007975
Aramis
Eden
Grant
Laska
Drakkar
Mogilevsky
L.angustifolium
L.bienne
Lirina
Orpheus

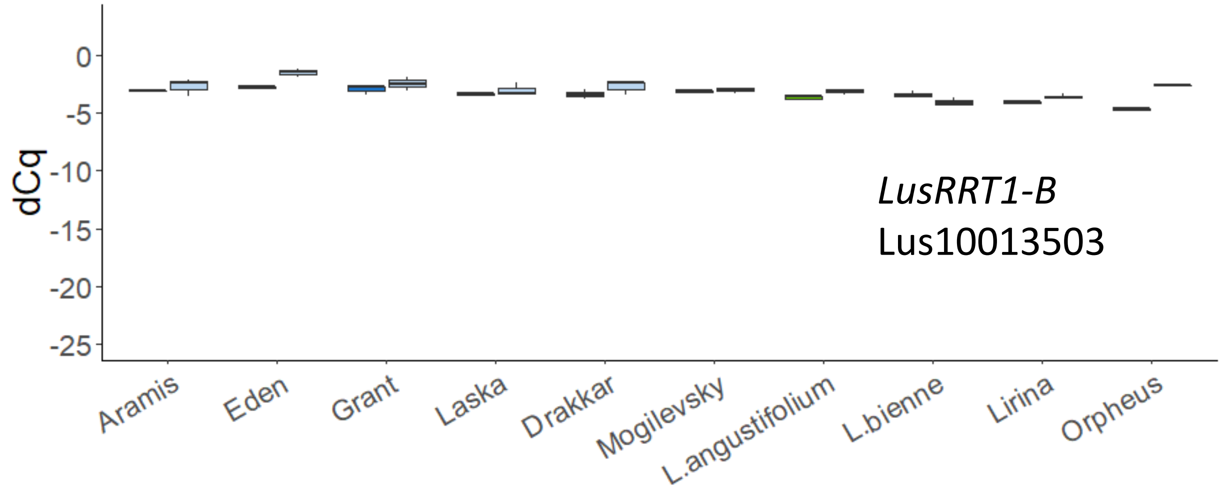

0
-5
-10
-15
-20
-25
dCq
LusRRT1-B
Lus10013503
Aramis
Eden
Grant
Laska
Drakkar
Mogilevsky
L.angustifolium
L.bienne
Lirina
Orpheus

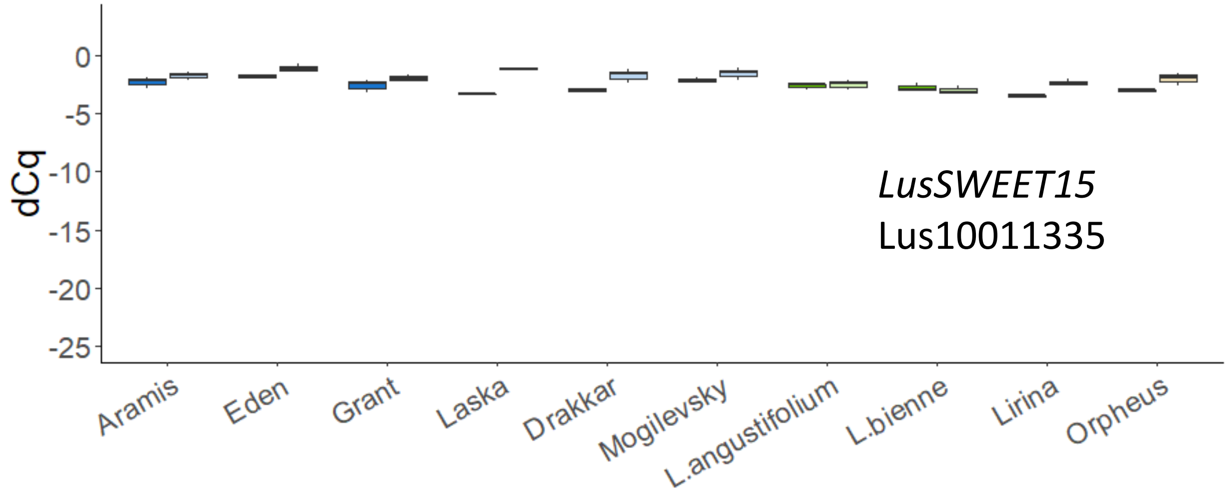

0
-5
-10
-15
-20
-25
dCq
LusSWEET15
Lus10011335
Aramis
Eden
Grant
Laska
Drakkar
Mogilevsky
L.angustifolium
L.bienne
Lirina
Orpheus

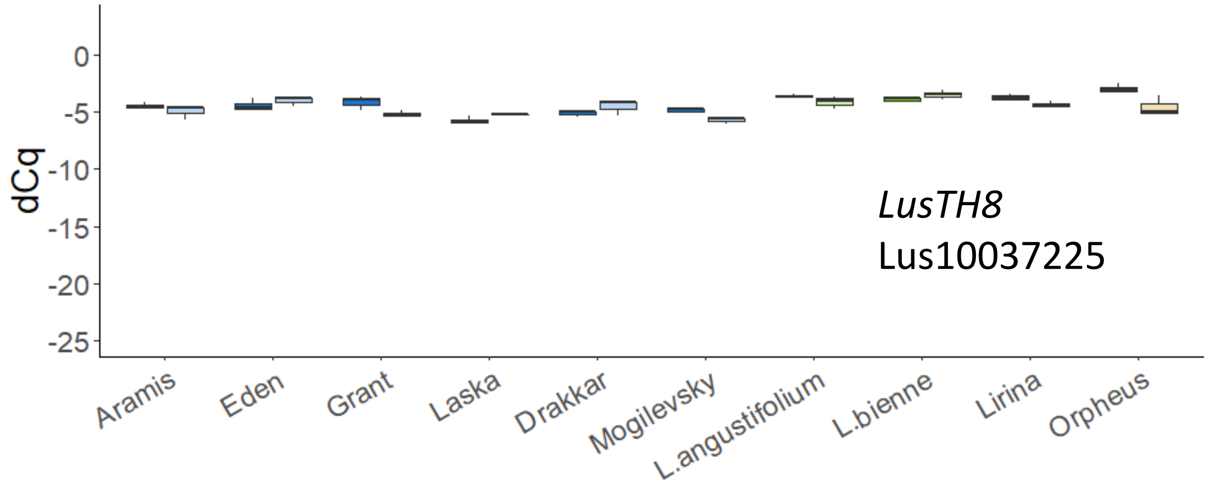

0
-5
-10
-15
-20
-25
dCq
LusTH8
Lus10037225
Aramis
Eden
Grant
Laska
Drakkar
Mogilevsky
L.angustifolium
L.bienne
Lirina
Orpheus

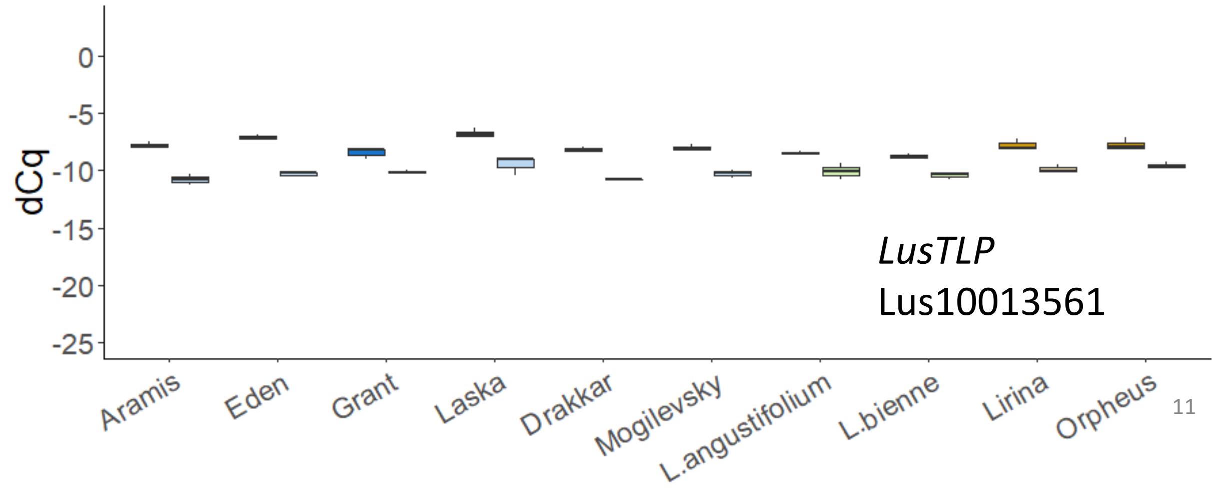

0
-5
-10
-15
-20
-25
dCq
LusTLP
Lus10013561
Aramis
Eden
Grant
Laska
Drakkar
Mogilevsky
L.angustifolium
L.bienne
Lirina
Orpheus

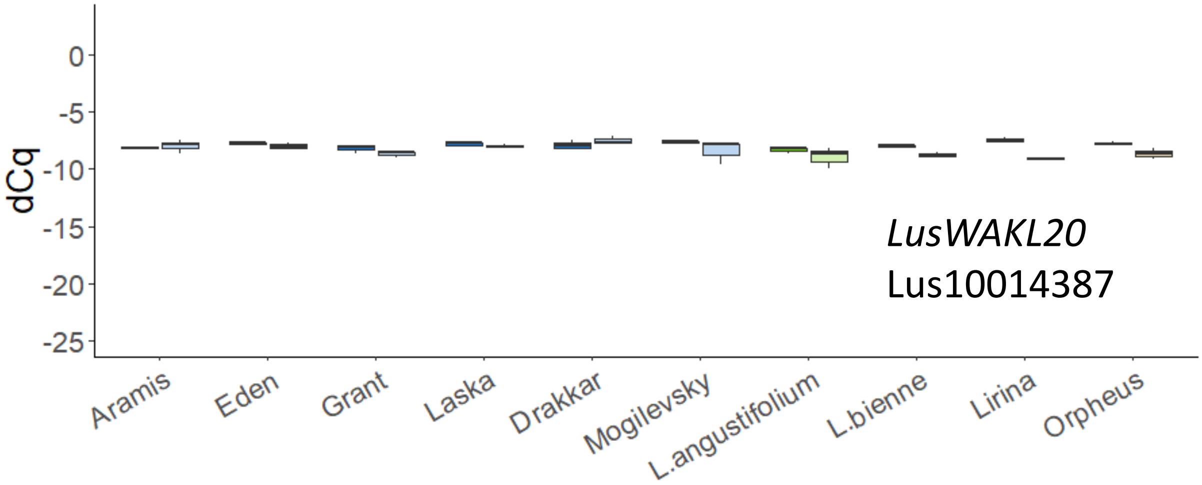

0
-5
-10
-15
-20
-25
dCq
LusWAKL20
Lus10014387
Aramis
Eden
Grant
Laska
Drakkar
Mogilevsky
L.angustifolium
L.bienne
Lirina
Orpheus

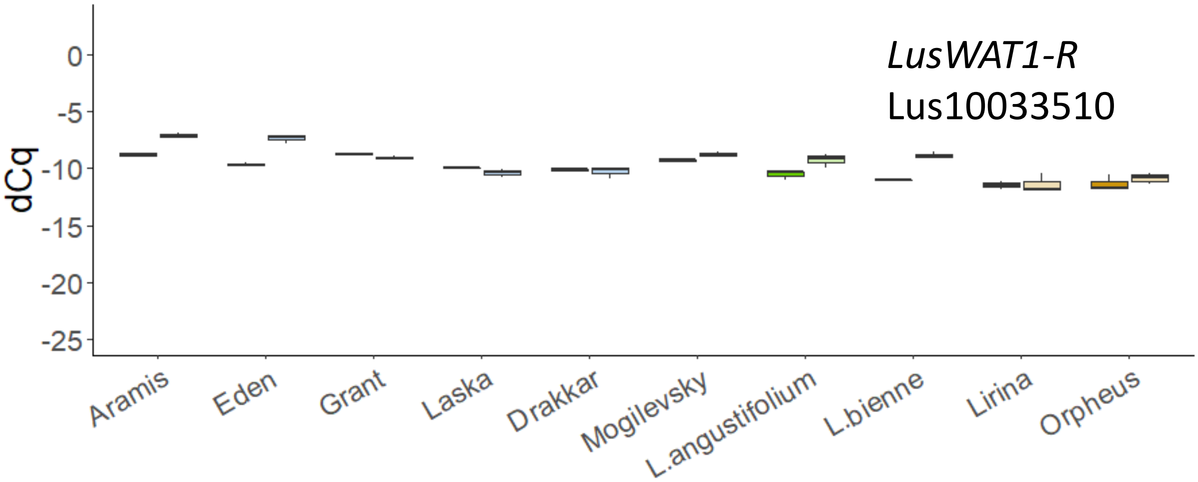

0
-5
-10
-15
-20
-25
dCq
LusWAT1-R
Lus10033510
Aramis
Eden
Grant
Laska
Drakkar
Mogilevsky
L.angustifolium
L.bienne
Lirina
Orpheus

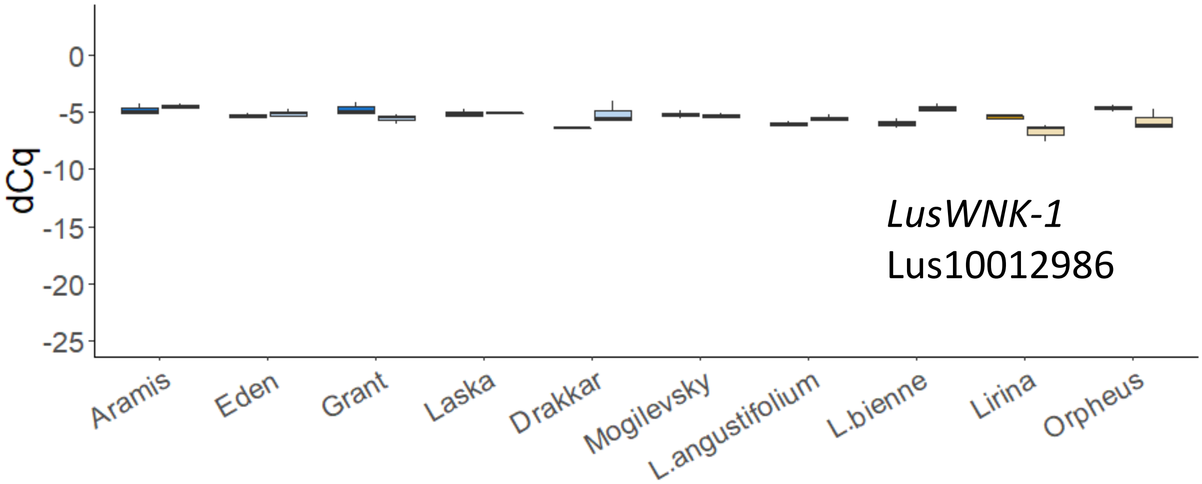

0
-5
-10
-15
-20
-25
dCq
LusWNK-1
Lus10012986
Aramis
Eden
Grant
Laska
Drakkar
Mogilevsky
L.angustifolium
L.bienne
Lirina
Orpheus

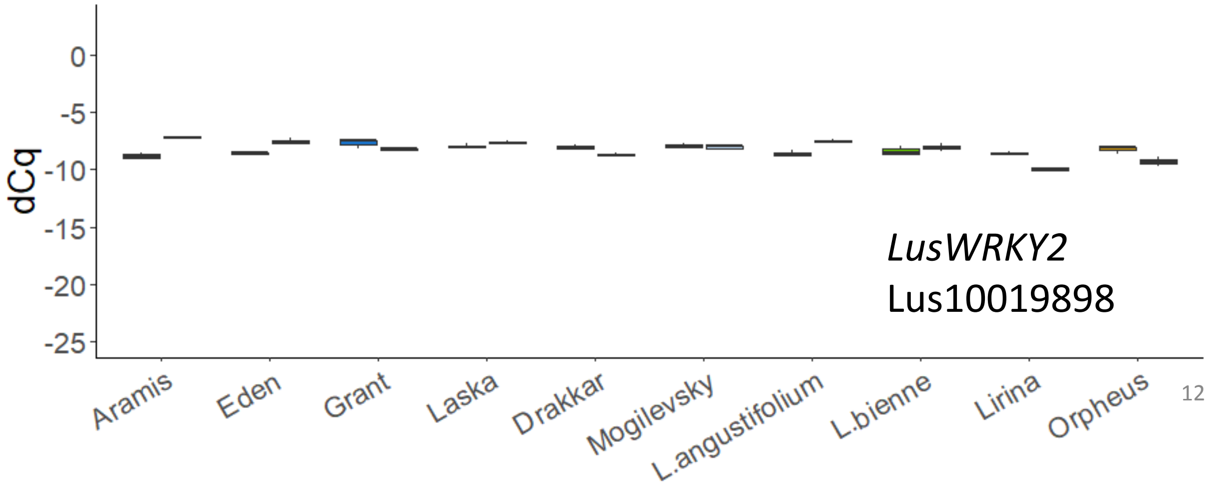

0
-5
-10
-15
-20
-25
dCq
LusWRKY2
Lus10019898
Aramis
Eden
Grant
Laska
Drakkar
Mogilevsky
L.angustifolium
L.bienne
Lirina
Orpheus

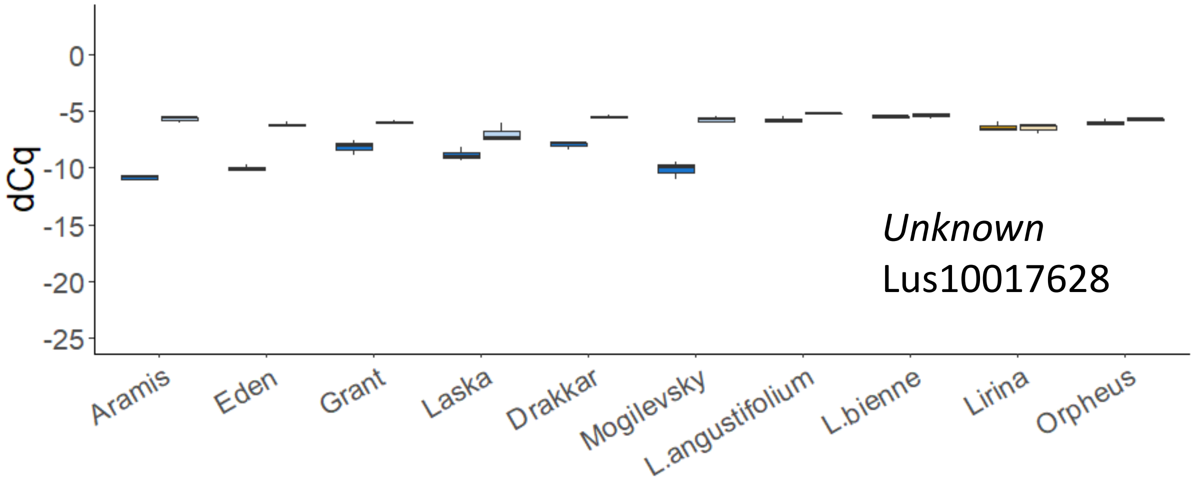

Figure S3. The relative expression levels (qPCR) of each studied gene (in alphabetical order) in all analyzed flax genotypes. The cultivars (species) relative expression level (log_2 – scale) of studied genes analyzed in 2018 and 2019 yy. Fiber flax cultivars are marked by blue color, linseeds - yellow, and wild species - green. Bright colors indicate samples of 2018, pale colors - 2019. The bottom and top of the box are the first and third quartiles, respectively; the bold line within the box is the median. The upper whisker extends from the third quartile to the largest value, but no further than 1.5 * IQR where IQR is the interquartile range. The lower whisker extends from the first quartile to the smallest value at most 1.5 * IQR. "Outlying" points are plotted individually.
