# Supplementary material for: The Toolbox for Fiber Flax Breeding: A Pipeline From Gene Expression to Fiber Quality
Source: Front Genet. 2020 Nov 12;11:589881. doi: 10.3389/fgene.2020.589881 (PMC7690631; doi:10.3389/fgene.2020.589881)
Supplement: Supplementary Figure 4 — The relative expression level of each studied gene (qPCR), averaged for three groups of flax genotypes (fiber cultivars, linseed cultivars, wild species). [file Data_Sheet_4.PDF]

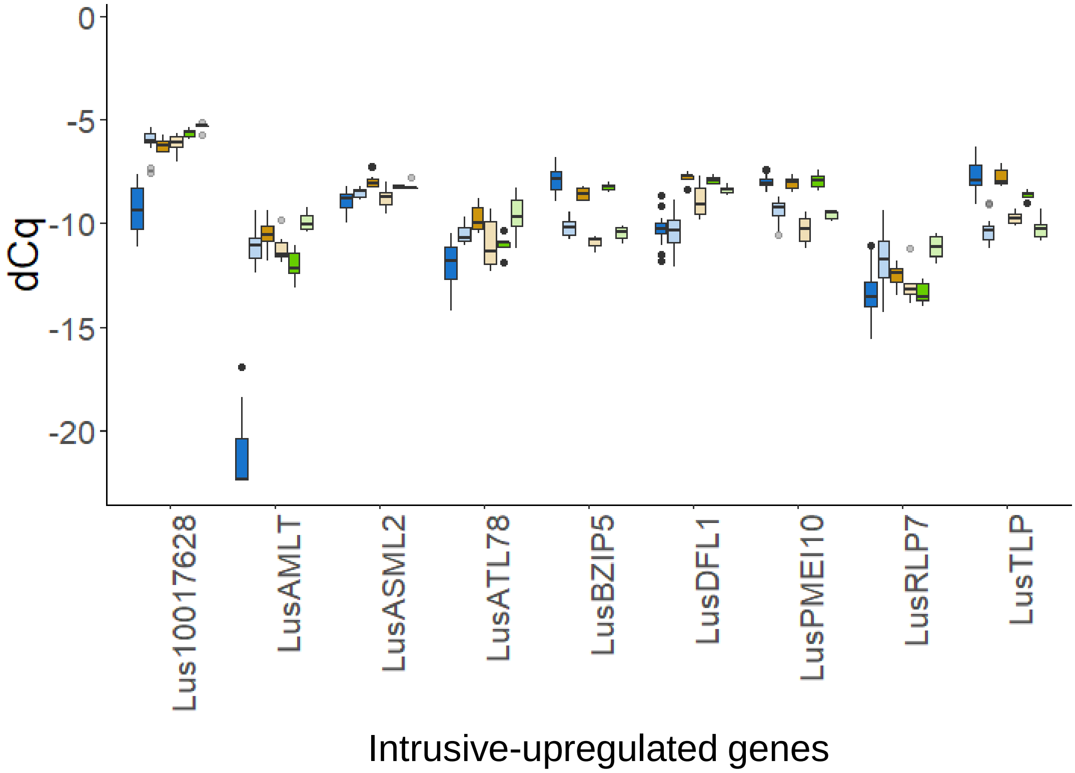
0
-5
-10
-15
-20
dCq
Lus10017628
LusAMLT
LusASML2
LusATL78
LusBZIP5
LusDFL1
LusPMEI10
LusRLP7
LusTLP
Intrusive-upregulated genes

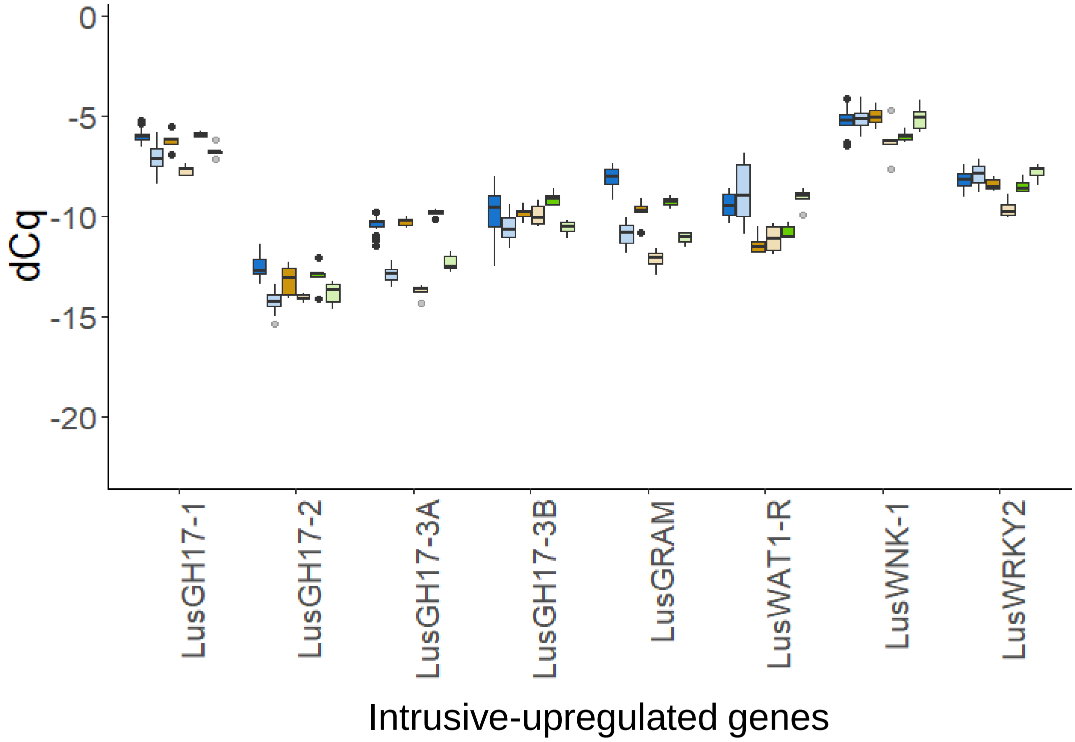
0
-5
-10
-15
-20
dCq
LusGH17-1
LusGH17-2
LusGH17-3A
LusGH17-3B
LusGRAM
LusWAT1-R
LusWNK-1
LusWRKY2
Intrusive-upregulated genes

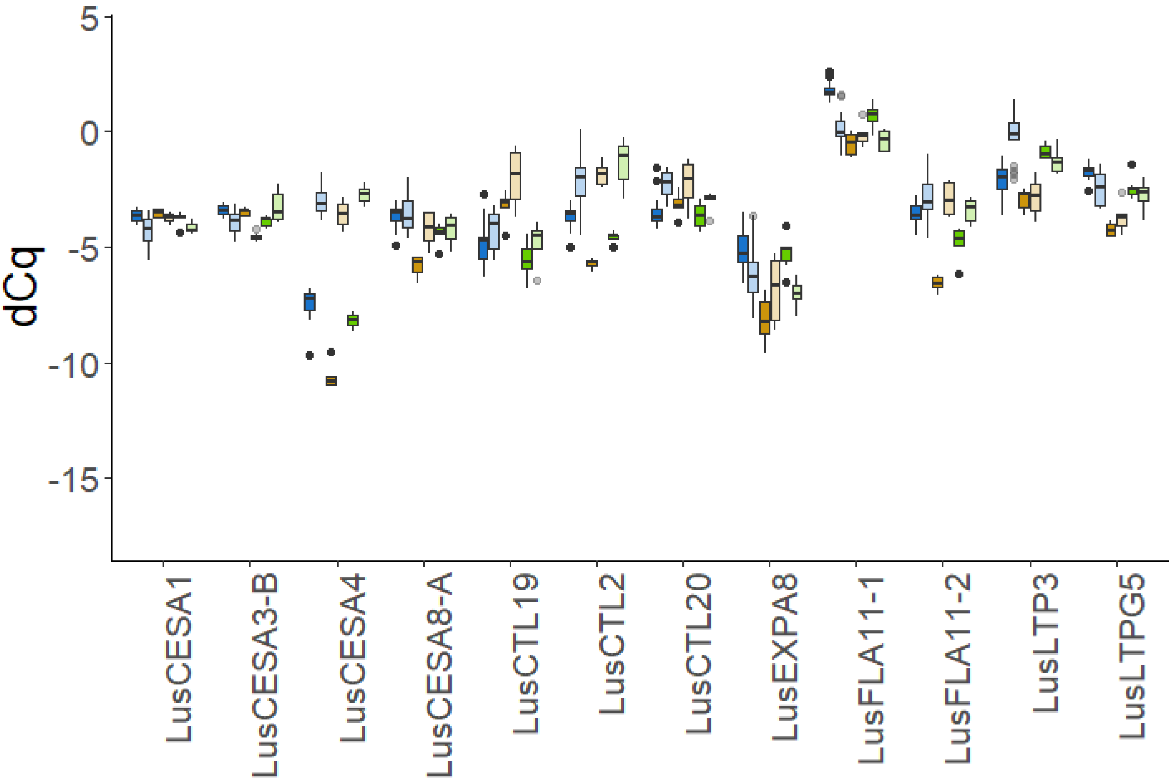

Tertiary Cell Wall upregulated genes. Genes related to cellulose biosynthesis

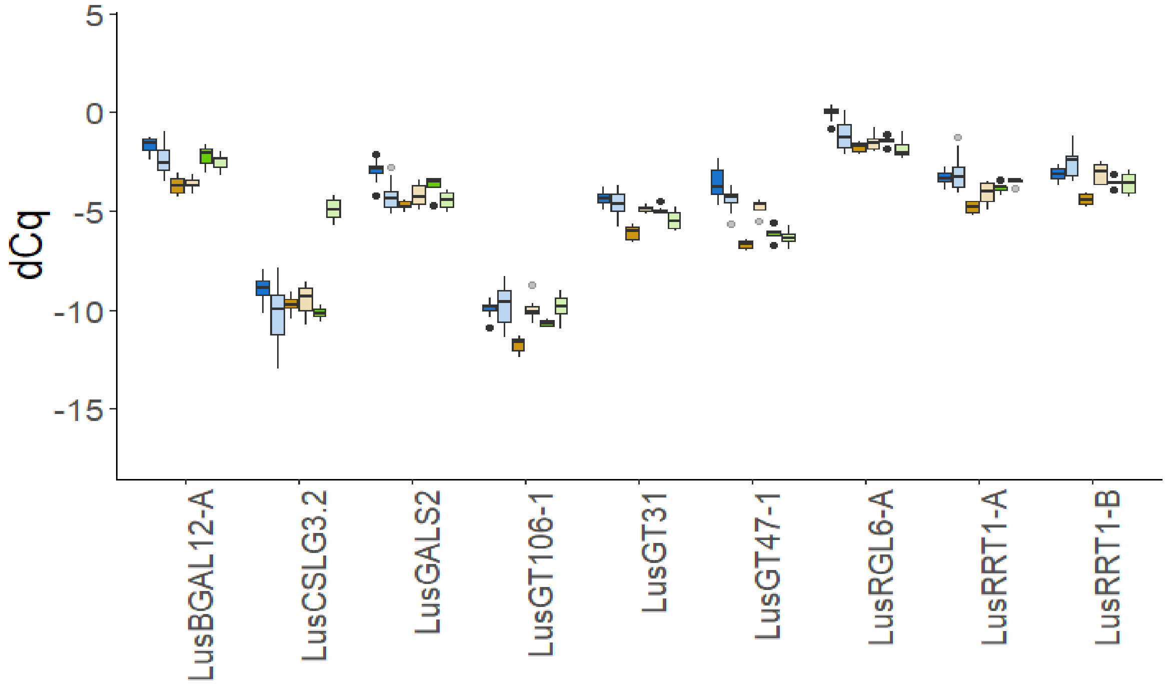

Tertiary Cell Wall upregulated genes. Genes related to rhamnogalacturonan-I biosynthesis and modification

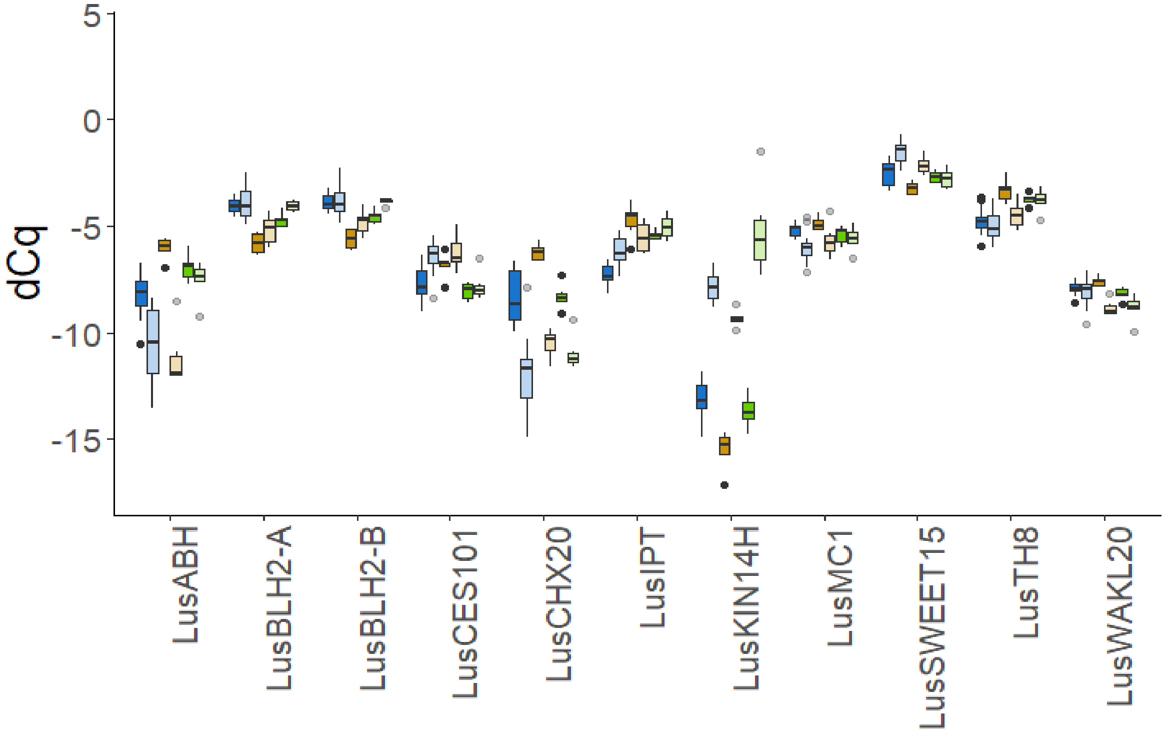

Figure S4. The relative expression level of each studied gene (qPCR), averaged for three groups of flax genotypes (fiber cultivars, linseed cultivars, wild species). Relative expression level (log2 – scale) of selected genes that were up-regulated at intrusive growth and tertiary cell wall biosynthesis stages. The plot presents summarized data among FLW groups. Blue color marks fiber flax cultivar group, yellow – linseed, and green – wild flax group. Bright colors indicate samples of 2018, pale colors - 2019. The bottom and top of the box are the first and third quartiles, respectively; the bold line within the box is the median. The upper whisker extends from the third quartile to the largest value, but no further than 1.5 * IQR where IQR is the interquartile range. The lower whisker extends from the first quartile to the smallest value at most 1.5 * IQR. "Outlying" points are plotted individually.
