# Supplementary material for: The Toolbox for Fiber Flax Breeding: A Pipeline From Gene Expression to Fiber Quality
Source: Front Genet. 2020 Nov 12;11:589881. doi: 10.3389/fgene.2020.589881 (PMC7690631; doi:10.3389/fgene.2020.589881)
Supplement: Supplementary Figure 5 — The relation between the technical stem length, the technical fiber strength, and flexibility and the expression level (ΔCq-value) of the selected genes in members of FLW groups. [file Data_Sheet_5.PDF]

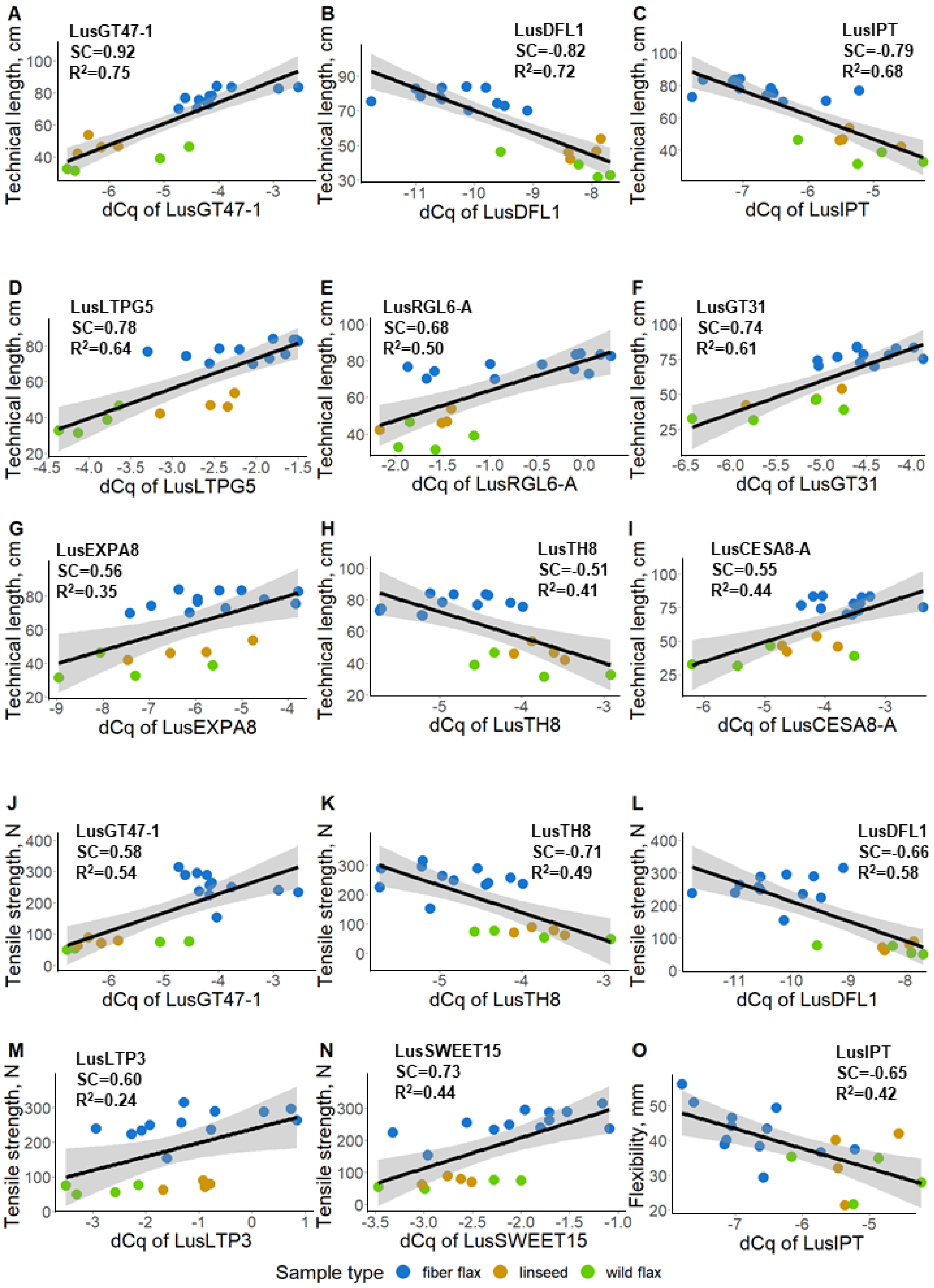

Figure S5. The relation between the technical stem length, the technical fiber strength, and flexibility and the expression level (ΔCq-value) of the selected genes in members of FLW groups. Blue dots – fiber flax, yellow – linseed flax, and green – wild flax. The regression line is plotted with a black line, and a shadow near the line indicates the range of 95% confidence interval.
