# Supplementary material for: The Toolbox for Fiber Flax Breeding: A Pipeline From Gene Expression to Fiber Quality
Source: Front Genet. 2020 Nov 12;11:589881. doi: 10.3389/fgene.2020.589881 (PMC7690631; doi:10.3389/fgene.2020.589881)
Supplement: Supplementary Figure 6 — Dependence modeling between tensile strength, flexibility, and technical length (used as dependent variable) and relative expression level (ΔCq-value) of Intrusive-upregulated and TCW-upregulated genes (used as independent variables) using LASSO method. [file Data_Sheet_6.PDF]

Figure S6. Dependence modeling between tensile, strength, flexibility, and technical length (used as dependent variable) and relative expression level (ΔCq-value) of Intrusive-upregulated and TCW-upregulated genes (used as independent variables) using LASSO method. Each line on the plots represents a path of one random variable. The upper plot on each figure aka Penalization path: every line corresponds to one random variable and shows the values taken by the regression coefficients of our model (Y-axis) given the values (log of) of the regularization parameter lambda (X-axis). Lambda is the weight given to the regularization term (the L1 norm) of LASSO regression, so as lambda approaches zero, the loss function of our model approaches the ordinary least squares (OLS) loss function. Therefore, when lambda is very small, the LASSO solution should be very close to the OLS solution, and all of your coefficients are in the model. As lambda grows, the regularization term has a greater effect and you will see fewer variables in the model (because more and more coefficients will be zero-valued).

The bottom plot on each figure aka Stability path: every line corresponds to one random variable and shows the stability scores given the values (log of) of the regularization parameter Lambda (X-axis). So the X-axis is identical to the previous plot, while Y-axis shows the stability score aka selection probability of the random variable: how probable is a given variable to be selected as a predictor. 2 cut-off lines set thresholds for selecting a set of stable predictors: Horizontal line set a stability threshold and the vertical line set a threshold on the regularisation parameter Lambda, which ensures the desired per-family type I error rate (set to 0.1 in our case), which is used for controlling the number of falsely selected variables.

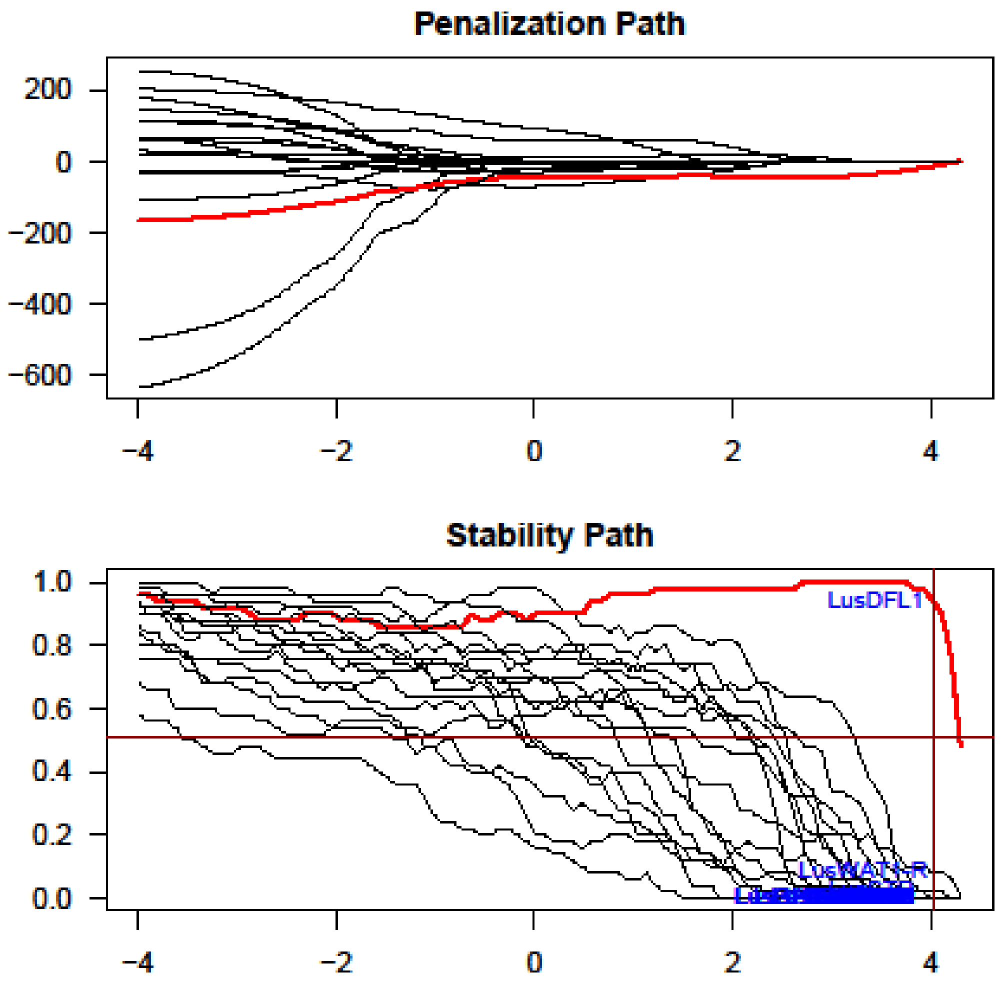

Tensile strength ~ Intrusive-upregulated genes

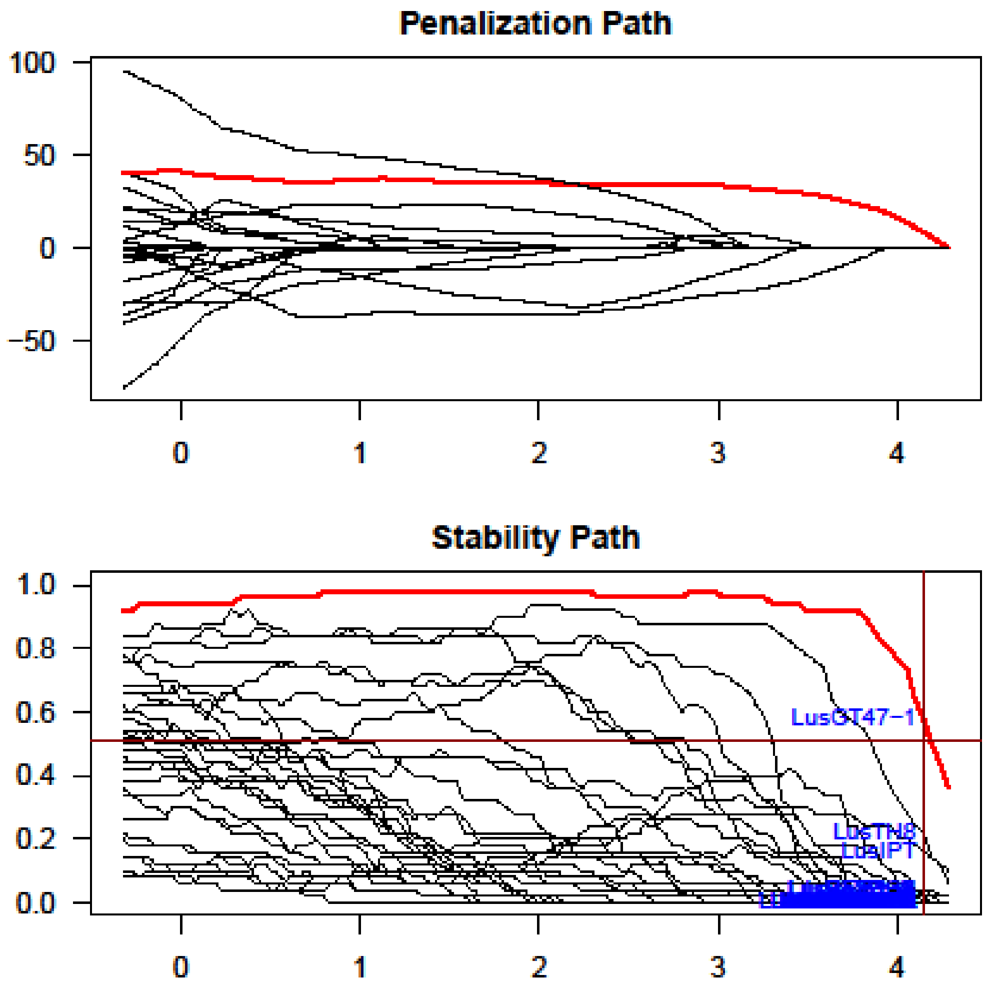

Tensile strength ~ TCW-upregulated genes

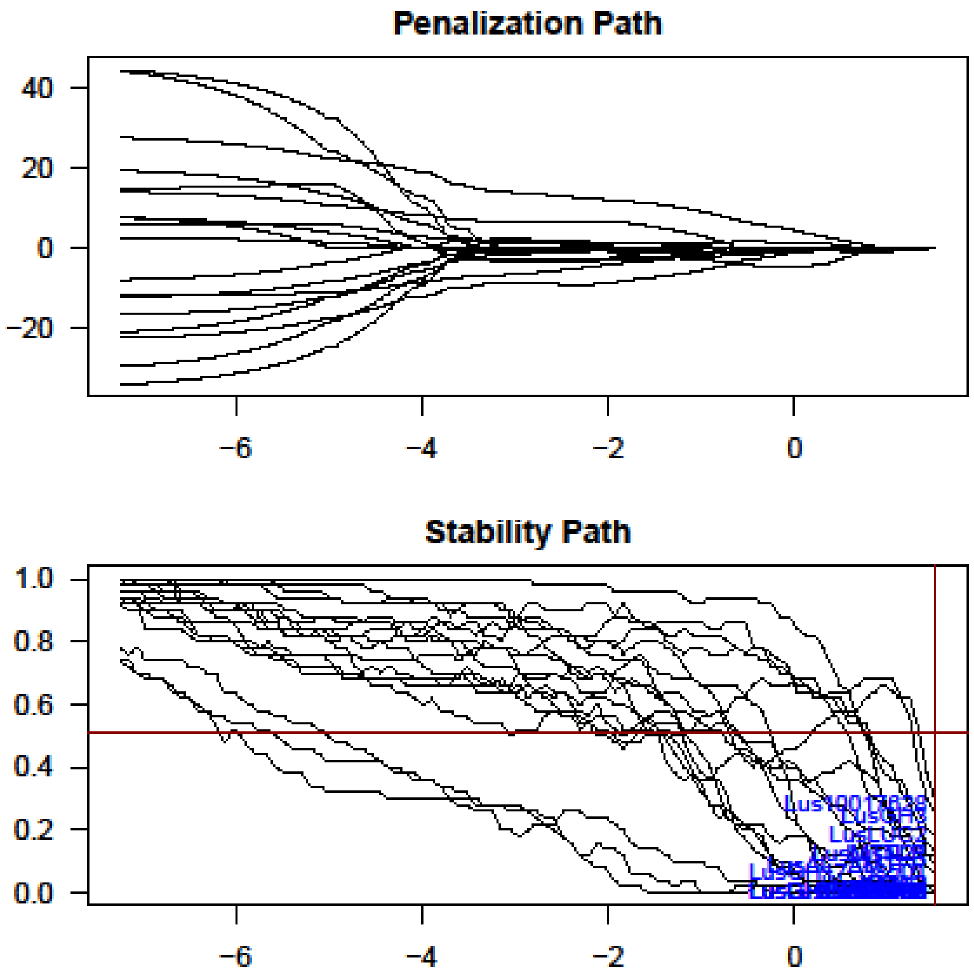

Flexibility ~ Intrusive-upregulated genes

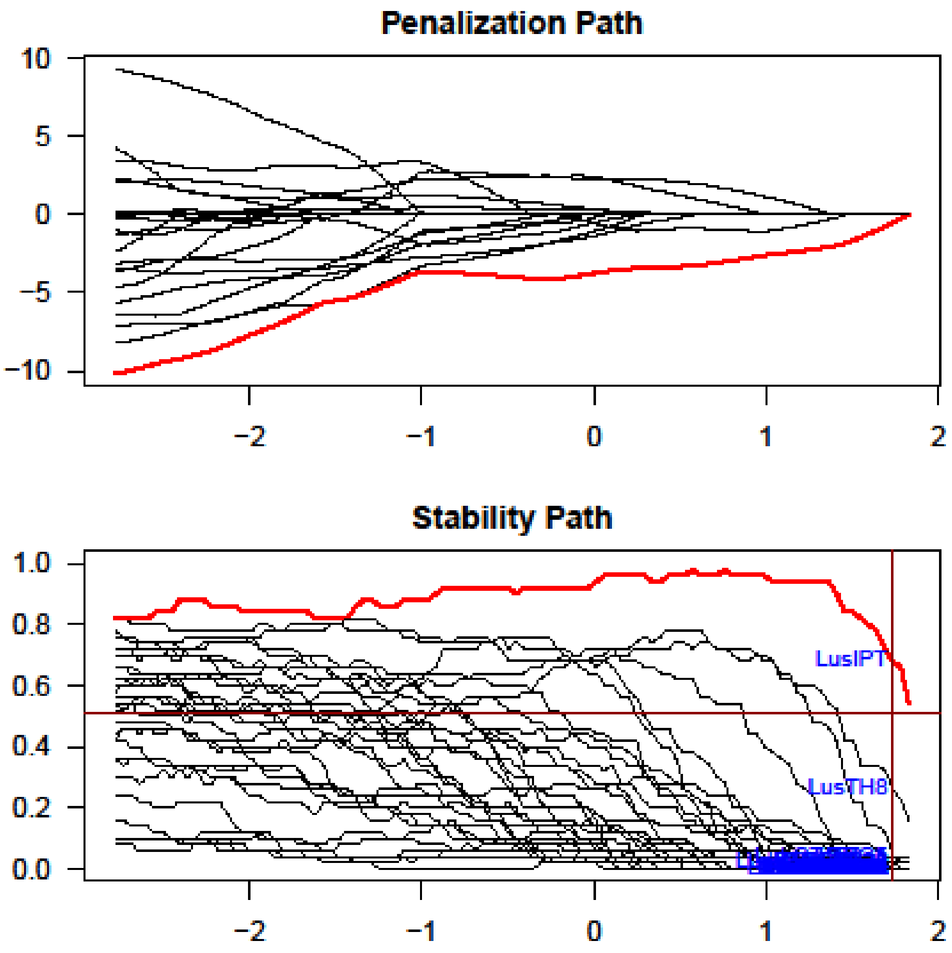

Flexibility ~ TCW-upregulated genes

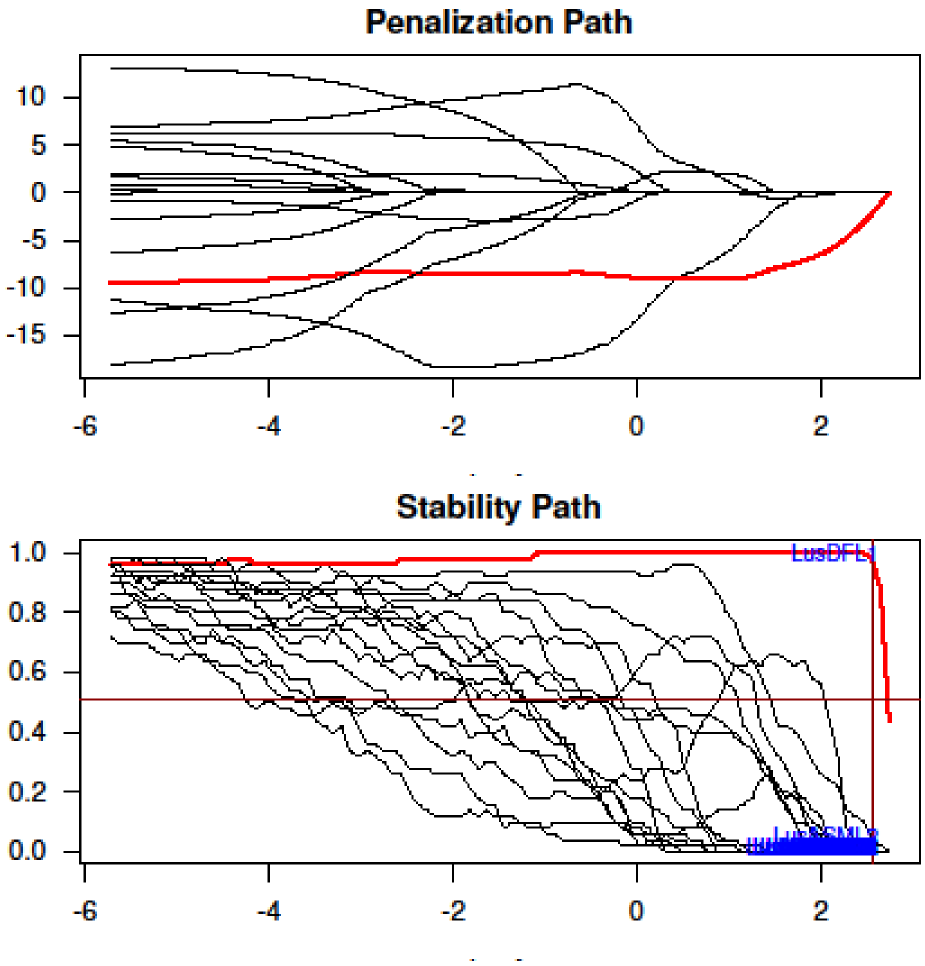

Technical length ~ Intrusive-upregulated genes

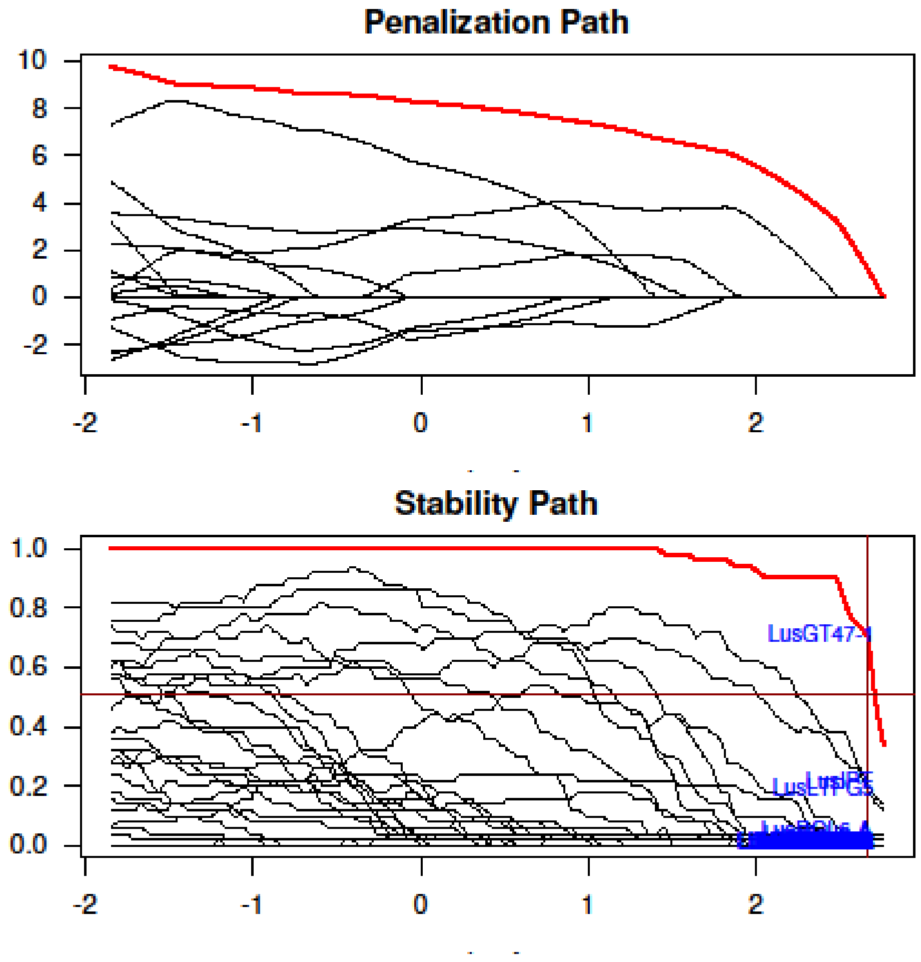

Technical length ~ TCW-upregulated genes
