# Supplementary material for: The Toolbox for Fiber Flax Breeding: A Pipeline From Gene Expression to Fiber Quality
Source: Front Genet. 2020 Nov 12;11:589881. doi: 10.3389/fgene.2020.589881 (PMC7690631; doi:10.3389/fgene.2020.589881)
Supplement: Supplementary Figure 7 — Linear regression of flax phenotypic traits on relative expression level (ΔCq-value). [file Data_Sheet_7.PDF]

Figure S7. Linear regression of flax phenotypic traits on relative expression level (ΔCq-value).

Tensile strength of scutched fibers:

(1) LASSO: Tensile strength ~ *LusDFL1 + LusGT47-1*

	Dependent variable:
	Tensile Strength
LusDFL1	-38.712*
	(19.606)
LusGT47.1	28.195
	(19.918)
Constant	-51.135
	(268.769)
Observations	20
R2	0.627
Adjusted R2	0.583
Residual Std. Error	63.566 (df = 17)
F Statistic	14.269*** (df = 2; 17)
Note:	*p<0.1; **p<0.05; ***p<0.01

(2) Stepwise: Tensile strength ~ *LusDFL1 + LusTH8 + LusWNK-1 + LusSWEET15 + LusCESA4 + LusLTP3 + LusCTL2 + LusABH + LusCTL19 + LusCESA8-A + LusFLA11-2 + LusCESA1*

	Dependent variable:
	Tensile Strength
LusDFL1	-14.378
	(8.372)
LusTH8	-37.504**
	(11.483)
LusWNK-1	78.981***
	(12.155)
LusSWEET15	27.593*
	(11.863)
LusCESA4	-47.929***
	(13.685)
LusLTP3	31.724***

```
                         (8.041)

LusCTL2              27.249**
                        (11.101)

LusABH              -30.027***
                         (6.422)

LusCTL19             -18.721*
                         (8.506)

LusCESA8-A      -58.141***
                        (14.118)

LusFLA11-2        59.913*
                        (26.847)

LusCESA1          -34.553
                        (35.426)

Constant             -270.324
                        (153.826)

-----------------------------------------------
Observations                   20
R2                          0.990
Adjusted R2                 0.972
Residual Std. Error       16.381 (df = 7)
F Statistic           56.557*** (df = 12; 7)
================================================
Note:               *p<0.1; **p<0.05; ***p<0.01
```

Flexibility of scutched fibers:

(1) LASSO: Flexibility ~ *LusIPT*

```
================================================
                    Dependent variable:
                 ---------------------------
                         Flexibility
-----------------------------------------------
LusIPT                 -5.673***
                         (1.581)

Constant                3.675
                         (9.798)

-----------------------------------------------
Observations                   20
R2                          0.417
Adjusted R2                 0.385
Residual Std. Error       7.112 (df = 18)
F Statistic           12.878*** (df = 1; 18)
```

```
=============================================
Note:          *p<0.1; **p<0.05; ***p<0.01
```

(2) Stepwise: Flexibility ~ *LusIPT + LusKIN14H + LusAMLT + LusDFL1*

```
=============================================
                Dependent variable:
             ---------------------------
                   Flexibility
---------------------------------------------
LusIPT              -3.531
                    (2.557)

LusKIN14H            1.973***
                    (0.595)

LusAMLT             -1.702**
                    (0.697)

LusDFL1              2.345
                    (1.733)

Constant            37.053**
                   (14.883)

---------------------------------------------
Observations              20
R2                      0.667
Adjusted R2                0.579
Residual Std. Error     5.886 (df = 15)
F Statistic         7.522*** (df = 4; 15)
=============================================
Note:          *p<0.1; **p<0.05; ***p<0.01
```

Technical length of stem:

(1) LASSO: Technical length of stem ~ *LusDFL1 + LusGT47-1*

```
=============================================
                Dependent variable:
             ---------------------------
             Technical stem length
---------------------------------------------
LusDFL1             -6.572**
                    (2.568)

LusGT47-1            8.010***
                    (2.609)

Constant            39.620
                   (35.201)

---------------------------------------------
```

Observations	20
R2	0.822
Adjusted R2	0.801
Residual Std. Error	8.325 (df = 17)
F Statistic	39.276*** (df = 2; 17)

===

Note:	*p<0.1; **p<0.05; ***p<0.01

(2) Stepwise: Technical length of stem ~ *LusGT47-1* + *LusWRKY2* + *LusLTP3* + *LusWAKL20* + *LusLTPG5*

	Dependent variable: Technical stem length
LusGT47-1	10.634***
	(1.335)
LusWRKY2	3.785
	(2.388)
LusLTP3	3.600***
	(1.011)
LusWAKL20	7.555***
	(2.452)
LusLTPG5	4.105*
	(2.010)
Constant	223.091***
	(22.566)
Observations	20
R2	0.955
Adjusted R2	0.939
Residual Std. Error	4.593 (df = 14)
F Statistic	59.996*** (df = 5; 14)

Note:	*p<0.1; **p<0.05; ***p<0.01
